# Sleep Disorders in Connective Tissue Diseases—Coexisting Diseases or Disease Components?

**DOI:** 10.3390/jcm13133656

**Published:** 2024-06-22

**Authors:** Hanna Cholerzyńska, Wiktoria Zasada, Konstantinos Tselios, Bogna Grygiel-Górniak

**Affiliations:** 1 Department of Rheumatology, Rehabilitation and Internal Diseases, Poznan University of Medical Sciences, 61-701 Poznan, Poland; 2 Department of Medicine, McMaster University, Hamilton, ON L8N 3Z5, Canada

**Keywords:** sleep disorders, quality of life, systemic lupus erythematosus, rheumatoid arthritis, connective tissue disease

## Abstract

This comprehensive review examines the complex relationship between sleep disorders and rheumatic diseases, supported by findings from the latest research articles. It encompasses various rheumatic conditions, including rheumatoid arthritis, systemic lupus erythematosus, and systemic sclerosis. The review reveals the bidirectional relationship between sleep disorders and these diseases, emphasizing their impact on disease progression and quality of life. Conventional and alternative therapeutic interventions for connective tissue diseases are presented, focusing on improving sleep quality and alleviating rheumatic symptoms. The role of pro-inflammatory cytokines and their potential modulation through pharmacological agents is also discussed. In the treatment of sleep disorders, various options are proposed, such as cognitive behavioral therapy for insomnia, physical activity, dietary modifications, and alternative approaches like reflexology and acupuncture. Thus, this review offers a nuanced understanding of the connection between sleep disorders and rheumatic diseases, supported by evidence from diverse studies. Such an approach is particularly important because it enhances sleep quality for overall patient well-being in the holistic management of rheumatic conditions.

## 1. Introduction

Connective tissue diseases (CTDs) are characterized by chronic immune-mediated inflammation in various organs, manifesting as systemic symptoms [1]. This involvement of multiple organs correlates with chronic fatigue and weakness, which are commonly associated with sleep problems. Sleep disorders in the general population pose a societal challenge, but their prevalence is notably higher in individuals with CTDs, constituting a substantial burden for the healthcare system. The types of sleep disorders and their occurrence vary between different rheumatic diseases; however, the most prevalent are insomnia, excessive daytime sleepiness (EDS), obstructive sleep apnea (OSA), and restless leg syndrome (RLS) [2].

Despite intensive research, many questions about the molecular basis of sleep disorders in connective tissue diseases still remain unanswered. Our current understanding suggests that prolonged inflammatory states, increased cytokine levels, chronic pain causing sleep deprivation, and the adverse effects of medications contribute to sleep disturbances [2]. However, advancements in treatment options and ongoing research focused on the pathologic background of autoimmune diseases offer hope for timely diagnosis and more effective management of sleep disorders.

Regrettably, the occurrence of sleep problems and declines in overall health status, including pain, fatigue, depression, and anxiety, are underestimated in patients with rheumatic diseases [3]. Thus, this review aims to describe the pathologic background of sleep disorders within the most common CTDs, such as rheumatoid arthritis (RA), systemic sclerosis (SSc), systemic lupus erythematosus (SLE), Sjögren’s syndrome (SS), and polymyositis/dermatomyositis (PM/DM). This manuscript comprehensively explores the intricate relationships between rheumatic diseases and sleep problems, providing an overview of this critical aspect of patient care

## 2. Materials and Methods

**Figure 1:**
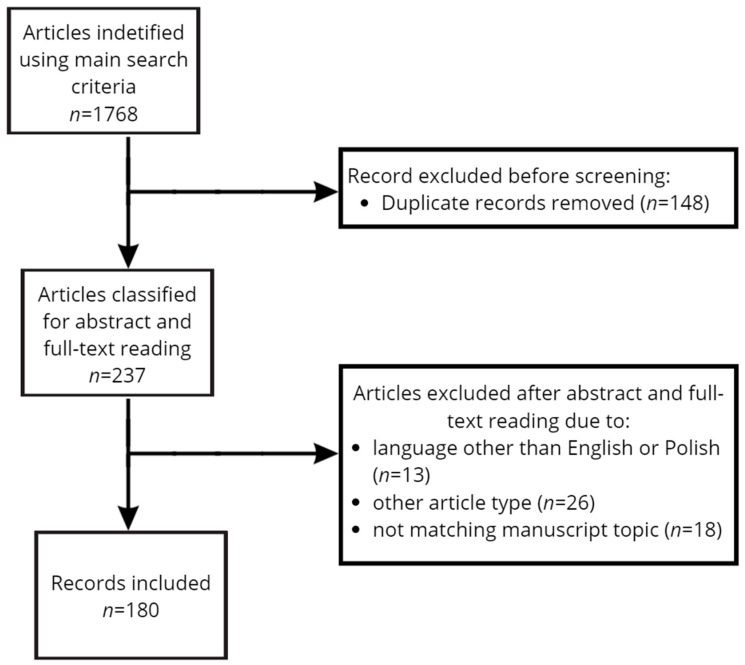
Flow diagram of the search and inclusion of references.

An electronic literature search was carried out using PubMed, Google Scholar, Scopus, and Medline databases to analyze data published between January 1998 and December 2023 on the pathogenesis, clinical symptoms, diagnosis, and therapeutic options for certain CTDs (RA, SSc, SLE, PM/DM). Published articles were in English or Polish. Data on the etiology, clinical manifestations, and treatment of sleep disorders in the context of CTDs were collected and analyzed. MeSH (medical subject heading) terms included, for the disease category, the following: “rheumatoid arthritis”, “systemic sclerosis”, “systemic lupus erythematosus”, and “polymyositis/dermatomyositis”. And those in the sleep disorder category included the following: “insomnia”, “excessive daytime sleepiness”, “obstructive sleep apnea”, and “restless leg syndrome”. Conjunction words, like AND and OR, were used to refine the search, systematically pairing each term from the disease category with each term from the sleep disorder category. Original studies, meta-analyses, randomized controlled trials (RCTs), case reports, and systematic review articles were selected for this review. Initially, articles’ titles were screened for accuracy. Papers that met the criteria for evaluation were further analyzed through their abstracts and full texts. This process was conducted independently by two authors, with discrepancies resolved through discussion or a third author. Manuscripts in both English and Polish were included in this analysis. The search strategy is presented in Figure 1. Quality assessment was performed using established criteria to evaluate the research question, the utilized methodology, the performed data analysis, the reported results, and the limitations (Supplementary Table S1). Exclusion criteria included papers that did not report the complications of rheumatic diseases and related sleep problems or were written in a language other than English.

## 3. Epidemiology of Sleep Disorders in CTDs

The most common sleep disorders in patients with CTDs are similar to those prevalent in the general population. They include insomnia, EDS, OSA, and RLS [2]. However, the prevalence of these disorders varies between different rheumatic diseases. This variation depends on factors such as the methodology and definitions used in studies, whether the study is prospective or retrospective, the specificity of a study group (i.e., related to a specific rheumatic disease), and the number of patients [4]. 

Although sleep disorders are not exclusive to individuals with CTDs, recent epidemiological findings suggest a higher prevalence of certain sleep disorders within this patient population (Table 1). For instance, Santilli et al.’s comparative analysis showed that obstructive sleep apnea is more prevalent in patients with CTDs than in the general population [5]. Moreover, insomnia and OSA are primarily observed in patients with systemic lupus erythematosus, whereas RLS is more common in those with systemic sclerosis. 

Unfortunately, data on specific sleep disorders in patients with polymyositis or dermatomyositis are lacking. Only one study has reported the general prevalence of sleeping disorders in idiopathic inflammatory myopathies (IIMs), indicating a prevalence of around 51% [6]. The scarcity of data can be attributed to the low prevalence of IIM in the general population. Similarly, detailed data on the prevalence of insomnia and excessive daytime sleepiness in systemic sclerosis patients are not available. However, numerous studies report the occurrence of “poor sleep quality”—68%, “difficulty sleeping”—76%, “fatigue”—89%, and “reduced sleep efficiency”—82% among these patients [7,8,9].

**Table 1:** Prevalence of sleep disorders in specific rheumatologic disorders.

Disorder	Prevalence of			
	Insomnia (%)	EDS (%)	OSA (%)	RLS (%)
RA	28.6 [10]	NA	12.7–21 [11,12]	30 [11]
SSc	NA	NA	32.1–58 [13,14]	40.7 [15]
SLE	33.3 [16]	28.5–35.8 [17,18]	50.0 [17]	34.2 [19]
Sjogren	71.0 [20]	15.3–55.0 [20,21]	45.0 [20]	15.3 [21]
General population	22.1 [22]	11.9 [21]	0.3 [5]	14.3 [23]

EDS—excessive daytime sleepiness; OSA—obstructive sleep apnea; RLS—restless leg syndrome; RA—rheumatoid arthritis; SSc—systemic sclerosis; SLE—systemic lupus erythematosus; NA—no available data.

## 4. Etiology of Sleep Disorders in Connective Tissue Diseases

Connective tissue diseases have a complex and often not well-understood etiology. Individual and genetic predispositions, environmental risk factors, and pro-inflammatory states partially constitute their background [24]. Notably, many pathological pathways demonstrate similarities between CTDs and sleep disorders. A key pathogenetic mechanism in CTD development involves impaired dendritic cell (DC) function, leading to the overproduction of inflammatory cytokines and joint tissue destruction mediated by T-cells and B-cells [25]. Recent data emphasize the role of various cytokines and autoantibodies in developing various rheumatic diseases [25,26]. Contributing factors to sleep disorders in CTD include the dysfunction of the hypothalamus–pituitary–adrenal (HPA) axis, increased fat tissue percentage, specific dietary habits, smoking, and genetic factors, all of which are explored below. The particular impact of depression and anxiety on the sleep quality of rheumatic patients also deserves emphasis.

### 4.1. Cytokines

Inflammatory cytokines and their interactions play a crucial role in the emergence of sleep disorders by inducing a pro-inflammatory response. Irwin et al. described a reciprocal relationship between increased levels of pro-inflammatory cytokines and the onset and depth of sleep [27]. Higher levels of IL-6, TNF-α, and IL-1β are linked to poorer sleep quality [27,28], and sleep deprivation itself causes an elevation in the nocturnal concentrations of these cytokines [27]. This reciprocal relationship helps explain why patients with CTDs often experience sleep disorders and why their rheumatic symptoms tend to worsen in the morning. 

Similar associations between sleep disorders and pro-inflammatory cytokines were described by Alt et al., who noted that increased IL-4 and TGF-β levels correlate with worsening sleep quality [29]. Moreover, patients with chronic insomnia exhibit elevated serum levels of inflammatory markers, including serum amyloid protein A (SAA), TNF-α, and granulocyte–macrophage colony-stimulating factor (GM-CSF). These levels correlate with symptom severity, supporting the theory that insomnia is related to increased inflammation [30]. 

**Figure 2:**
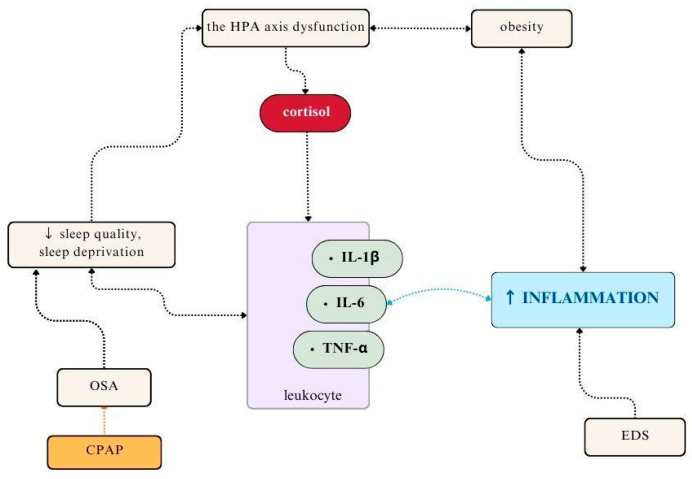
The role of inflammatory cytokines in sleep disorders. CPAP—continuous positive airway pressure; EDS—excessive daytime sleepiness; OSA—obstructive sleep apnea; the HPA axis—the hypothalamus–pituitary–adrenal axis.

Moreover, increased levels of pro-inflammatory cytokines (TNF-a, IL-6, IL-1b) are also observed in OSA and correlate with the severity of rheumatic symptoms, particularly pain and fatigue [31] (Figure 2). IL-6 levels independently correlate with a higher prevalence and severity of depression in this group of patients [32]. Interestingly, continuous positive airway pressure (CPAP) therapy in patients with OSA lowers the levels of pro-inflammatory and oxidative stress markers, including lipid, protein, and DNA oxidation products, C-reactive protein (CRP), IL-6, IL-8, TNF-α, nitric oxide (NO), nitrosative stress compounds, and markers of endothelial dysfunction [33]. Wali et al. found no alterations in inflammatory markers following one month of CPAP therapy, possibly due to the short duration of the treatment [34]. Recently, OSA has been recognized as a significant factor exacerbating inflammatory processes in RA patients [35]. Kang et al. showed that the risk of RA is higher in patients with sleep apnea (2.91%) compared to healthy subjects (1.53%), with a hazard ratio of 1.91 (95% CI = 1.32–2.77, *p* < 0.001) [36]. 

Another important aspect is excessive daytime sleepiness (EDS). It is a common condition that can occur independently or as a secondary symptom of a rheumatic disease [37]. Various pro-inflammatory cytokines, such as increased TNF-a and IL-6, can mediate sleepiness in disorders that coexist with EDS [38]. Elevated IL-6 levels are also found in patients with narcolepsy and OSA, both of which are conditions frequently accompanied by EDS [39,40,41]. However, there are conflicting data regarding this relationship. Alexopoulos et al. reported no association between levels of TNF-α and EDS development [42]. Similarly, de la Peña Bravo et al. found no significant differences in TNF-α, IL-6, and ICAM-1 in OSA patients with or without coexisting EDS [43]. Nonetheless, a study assessing objective daytime sleepiness in American patients using Multiple Sleep Latency Testing demonstrated increased IL-6 levels [44]. This association was not observed in subjective daytime sleepiness, assessed using the Epworth Sleepiness Scale [44]. Such disparities in the findings may be due to different methodological assessments, explaining variations in the correlation between daytime sleepiness and pro-inflammatory cytokine levels.

Interestingly, certain genetic factors can also influence the development of OSA. It is suggested that the high prevalence of EDS in patients with OSA might be associated with TNF-α (-308G) gene polymorphism [45]. However, recent data show an inverse association of the TNF-α-308A allele with EDS risk in patients with OSA [46]. 

Restless leg syndrome is another disorder reported by patients with connective tissue diseases [11,15,19,21]. However, the correlation of RLS with inflammation and pro-inflammatory cytokines remains controversial. While some data challenge this association, several studies confirm the involvement of inflammation in RLS, as evidenced by elevated levels of IL-1β, IL-6, and TNF-α [47,48]. Elevated CRP levels are linked to severe periodic leg movements (PLMs), suggesting that RLS patients with a high number of PLMs are prone to an excessive inflammatory state [49]. Additionally, increased oxidative stress (indicated by markers such as 8-hydroxy-2′-deoxyguanosine) and chronic inflammation, marked by high-sensitivity C-reactive protein, IL-6, and ferritin, predict RLS severity [50]. Interestingly, RLS is associated with elevated levels of TNF-α in patients exhibiting depressive symptoms, suggesting that this cytokine is a link between RLS and other comorbidities in rheumatic subjects [51]. Genetic studies further support these associations, confirming increased concentrations of IL-1 through IL-1B polymorphisms (rs1143643, rs1143634, or rs1143633) correlating with an elevated risk of RLS development [52]. Although definitive conclusions between sleep disorders and the immune mechanisms of rheumatic diseases remain elusive, the involvement of typical inflammatory pathways is clear.

### 4.2. Melatonin

A comprehensive discourse on sleep and sleep disorders inherently necessitates an exploration of melatonin, given its pivotal role as one of the substances intricately involved in the regulation of sleep patterns [53]. As discussed above, the relationship between sleep and inflammation is well described. However, it is crucial to note that the nocturnal melatonin secretion profile is unrelated to increased pro-inflammatory cytokines. Cytokine regulation depends mainly on the quantity and depth of sleep [54]. Despite this, the role of melatonin in developing autoimmune conditions has been a subject of discussion in recent years [55]. Melatonin can, in fact, induce increased cytokine production and disease progression, such as articular cartilage bone destruction and synovial hyperplasia [56,57]. 

Surprisingly, recent data showed the opposite effects of melatonin, revealing its antioxidant and anti-inflammatory properties, which have a beneficial impact on CTDs [58,59]. For instance, Liu et al. discovered an improvement in the function of salivary glands and reduced inflammation in an animal model of primary Sjögren’s syndrome after melatonin administration [60]. Similarly, in SLE, melatonin was associated with decreased oxidative stress parameters, specifically serum malondialdehyde levels; however, no beneficial effect on disease activity was described [61]. Thus, further randomized control trials are required to prove the beneficial effect of melatonin on autoimmune disorders and related sleep issues.

### 4.3. Hypothalamic–Pituitary–Adrenal Axis

The hypothalamic–pituitary–adrenal axis is an excellent model of circadian rhythms, as it closely aligns with the sleep–wake cycle [62,63]. Most rheumatic diseases exhibit a degree of baseline HPA glucocorticoid axis dysfunction before GC treatment [64,65,66]. Chronic inflammation, a hallmark of CTD, is also associated with sleep regulation through the HPA axis [67]. This axis is regulated by IL-6, with an increased level of IL-6 being related to more secretion of ACTH and cortisol [68]. Furthermore, elevated cortisol is related to sleep abnormalities. Interestingly, sleep deprivation negatively impacts the cortisol regulation of circadian rhythm by influencing the HPA axis, as observed in healthy patients [69].

### 4.4. Obesity

Recently, obesity has emerged as a significant global concern, imposing a substantial burden on healthcare systems and correlating with sleep disorders. Anthropometric data indicate that short sleep increases body mass and waist circumference [70]. Moreover, obesity itself has been associated with chronic inflammation [71,72], and sleep deprivation further correlates with abdominal visceral obesity and excessive energy intake [73]. Since rheumatic diseases are also related to sleep problems, obese patients with CTDs are at particular risk of developing sleep disorders. Epidemiological data underline the high prevalence of metabolic syndrome in rheumatic patients [74], and sleep impairment is independently linked to obesity in patients with rheumatic diseases such as SLE [75].

Multiple studies have shown that dietary interventions and exercise improve sleep quality in obese patients [76,77]. These improvements may be attributed to hormonal changes and reduced inflammatory cytokines [76]. An elimination diet (excluding meat, gluten, and dairy products) has been shown to reduce body mass, inflammation, and pain in patients with RA [78]. However, questions remain about the extent to which caloric restriction alone can decrease a pro-inflammatory state. Various studies have demonstrated that reducing daily caloric intake decreases inflammation associated with excessive adiposity [79,80]. Those findings suggest a complex but well-established relationship between obesity and CTDs. 

Obese patients experience higher snoring and motor activity levels at night, coupled with less quiet sleep [81]. However, observed sleep disorders result from snoring, disrupted breathing, and other sleep physiology disturbances [81]. Koh et al. showed that OSA in obese patients worsens tissue response to insulin, suggesting potential benefits from treatment targeting peripheral insulin resistance in individuals with OSA [82]. Interestingly, metformin, a first-line therapy for insulin resistance, has been found to improve salivary gland function and regulate lymphocyte activity in patients with Sjögren’s syndrome, presenting a possible treatment opportunity for sleep disorders [83]. Metformin also attenuates RA activity and inflammation, as evidenced by decreased CRP levels and a diminished disease activity score (DAS-28-CRP) [84]. Thus, the regulation of body mass has a positive influence on both sleep quality and the activity of rheumatic diseases.

### 4.5. Diet

Given the established connection between obesity and sleep problems, researchers have explored the impact of a low-calorie diet on sleep-related issues. Notably, both obesity and glucose metabolism can impact disease progression and sleep quality in patients with CTDs [85,86]. Furthermore, specific dietary constituents, such as red meat or potatoes, have been implicated in exacerbating sleep disturbances [85,86]. For instance, more meat consumption has been associated with lower sleep quality and sleep disturbances [87]. However, it is not solely products of animal origin that impact sleep quality. Recent findings indicate that an imbalanced diet, assessed using the alternative healthy eating index—2010 (AHEI-10) food group component and matrix score, is associated with sleep apnea [88,89]. Moreover, insomnia has been linked to elevated energy, trans fat, and sodium intake, coupled with reduced vegetable consumption [88,89]. A recent study demonstrated that a higher intake of dietary omega polyunsaturated fatty acids improves sleep quality in patients with SLE [90].

### 4.6. Smoking

Cigarette smoking emerges as a notable risk factor significantly impacting both sleep quality and disease progression among individuals with connective tissue diseases. Studies have consistently highlighted its detrimental effects on sleep quality, with smoking being associated with increased inflammation, a higher risk of SLE, accelerated radiographic progression in RA, and a worse response to MTX-based treatment [91,92,93,94]. Moreover, smoking is a common habit among RA patients, with nearly 26% being former smokers and around 14% current smokers [95]. Nicotine, a primary constituent of cigarettes, is strictly associated with sleep disturbances, with smokers generally exhibiting poorer sleep quality compared to non-smokers; in particular, night smokers experience pronounced sleep problems [96,97,98]. Concurrently, cigarette smoking also influences disease progression, with its association with obstructive sleep apnea (OSA) contributing to a higher risk of OSA in smoking patients, while OSA itself serves as a risk factor for smoking [99]. Although earlier data suggested a higher prevalence of OSA among smokers, recent findings indicate no significant difference in OSA prevalence between smokers and non-smokers [100]. Thus, the relationship between cigarette smoking and sleep disorders in CTD patients is intricate, with smoking exacerbating sleep quality issues while concurrently exerting negative effects on disease progression [101]. Large-scale randomized controlled trials are warranted to elucidate these complex interactions comprehensively.

### 4.7. Genetics

Numerous studies have underscored the significant role of genetic factors in the pathogenesis of rheumatic diseases. In particular, systemic lupus erythematosus (SLE) and rheumatoid arthritis (RA) have specific human leukocyte antigen (HLA) alleles implicated in disease development [102,103]. Interestingly, the prevalence of these alleles varies among ethnic groups, suggesting a potentially higher prevalence of HLA-dependent CTDs in Hispanic and African–American populations [102]. For instance, the presence of the HLA-DRB1*03:01 allele has been associated with a series of intracellular events that trigger and exacerbate SLE symptoms, including endoplasmic reticulum stress and malfunction in mitochondria, necroptosis, and pro-inflammatory cytokine production [104].

Moreover, genetic factors also play a crucial role in various aspects of sleep regulation [102,105]. Polymorphisms in HLA genes, such as HLA II DQB1*06:02, have been linked to narcolepsy, while a higher prevalence of HLA-B39 is associated with obstructive sleep apnea (OSA) [106,107]. Additionally, the occurrence of REM sleep behavior disorder, characterized by violent, complex dream-enacting behaviors and polysomnographic changes, has been related to HLA class II genes [108]. 

Recent data highlight the role of pro-inflammatory cytokine polymorphisms, which modulate sleep quality. Interleukin-6 and interleukin 1β are implicated in various pathological processes in CTDs, and polymorphisms in genes encoding these cytokines have been associated with sleep disorders. For instance, polymorphisms in IL-6 (rs1800796) and 5-hydroxytryptamine receptor 2A (rs6311) correlate with the severity of obstructive sleep apnea/hypopnea syndrome (OSAHS) [109]. Furthermore, genetic polymorphisms of IL-1β (rs1143643, rs1143634, and rs1143633) are associated with an increased risk of experiencing RLS symptoms [52]. These findings underscore the intricate interplay between genetic factors, inflammatory pathways, and sleep disturbances in the context of connective tissue diseases.

### 4.8. Orexins/Hypocretins

Orexins, also referred to as hypocretins, are neuropeptides with multifaceted roles encompassing regulating the sleep–wake cycle, feeding behaviors, stress response, mood regulation, and many other functions [110]. While the discussion on orexin/hypocretin is intriguing, its integration into the broader discourse on sleep regulation in connective tissue diseases (CTDs) warrants attention for enhanced cohesion. Although limited studies directly investigate the impact of orexins on sleep quality in CTD patients, available evidence permits speculation regarding their potential influence on both disease progression and sleep disturbances. 

One well-established pathomechanism involving orexin pertains to narcolepsy, characterized by a reduced number of orexin-secreting neurons [106]. Epidemiological data indicate a higher prevalence of autoimmune conditions, including SLE, psoriasis, multiple sclerosis, autoimmune thyroid disease, inflammatory bowel disease, and idiopathic thrombocytopenic purpura, among patients with narcolepsy [111]. Moreover, orexins participate in the modulation and perception of inflammatory pain, with elevated levels influencing body mass and inflammatory states [112,113,114]. Notably, orexins exhibit anti-inflammatory properties, as evidenced by their ability to reduce levels of IL-1β, IL-6, and IL-8 and suppress the production of reactive oxygen species in fibroblast-like synoviocytes [115]. Interestingly, the orexinergic response could be involved in the development of EDS in patients with SLE, while low orexin levels, coupled with elevated pro-inflammatory cytokine levels in cerebrospinal fluid (CSF), are implicated in fatigue generation in individuals with Sjögren’s syndrome [116,117]. Integrating the discussion of orexin/hypocretin into the broader framework of sleep regulation in CTDs not only enhances the overall coherence of the discourse but also facilitates a more comprehensive understanding of the intricate interplay between neurophysiological pathways and autoimmune processes underlying sleep disturbances in these conditions.

### 4.9. Depression and Anxiety

The well-documented relationship between sleep disorders, depression, and anxiety in the general population extends to individuals with connective tissue diseases (CTDs), necessitating a more direct connection with specific examples and pertinent studies [118,119]. Indeed, the prevalence of these comorbidities is notably elevated in CTDs, often intertwined with the complex symptomatology of these conditions. 

Chronic pain, a hallmark feature of many rheumatic disorders such as rheumatoid arthritis (RA) and systemic lupus erythematosus (SLE), is intricately linked to heightened rates of depression and anxiety [120,121]. For instance, SLE patients frequently report poor sleep quality, particularly if the disease is associated with reduced muscle strength and lower resting lung function [121]. The burden of fatigue is strikingly prevalent in SLE, affecting up to 90% of patients, while anxiety is reported in 35.9% of individuals with lupus [122]. 

Moreover, depression is prevalent among patients with Sjögren’s syndrome, especially in the presence of heightened disease activity and increased total pain burden [123]. Individuals with this condition often endure multiple nocturnal awakenings attributed to symptoms such as dry mouth and eyes, a phenomenon correlated with anxiety, depression, and increased fatigue [21]. By forging a more direct linkage between sleep disorders, depression, and anxiety with specific instances and relevant research in the context of CTDs, we can deepen our comprehension of the interplay between these interconnected domains and their collective impact on disease manifestation and patient well-being.

### 4.10. Pain

Pain is one of the most common symptoms reported by rheumatic patients, with its intensity varying depending on the specific disease and its activity [122,124]. The relationship between pain and sleep disturbances is bidirectional—insomnia or sleep restriction correlates with pain intensity and is related to an excessive inflammatory process [125,126]. Higher levels of IL-6 and TNF-α are directly associated with sleep cessation and the increased pain intensity reported by patients. This dual relationship is confirmed by observational studies [127]. Additionally, pain is widely associated with sleep disorders, a frequent intersection in rheumatic diseases such as RA, SLE, and Sjögren’s syndrome [128,129,130,131]. Nighttime awakening, due to sleep-related issues, is also found to be the only independent factor for predicting fatigue in Sjögren’s syndrome, aside from anxiety [21].

## 5. Drug-Induced Sleep Disorders

The sleep quality in patients with CTDs can be influenced by an adequate dose of glucocorticoids (GCs) and disease-modifying antirheumatic drugs (DMARDs), both conventional and biological. The anti-inflammatory effect of these drugs is the most crucial mechanism for decreasing the activity of connective tissue diseases and the risk of sleep disorder development. 

### 5.1. Glucocorticoids

Glucocorticosteroids are widely used in numerous rheumatic diseases for their immediate ability to reduce disease activity. However, they can induce insomnia, which is the most common complication, particularly potentiated by GC-induced obesity [132,133]. The adverse effects of glucocorticoids are dose-dependent, with an increased risk of sleep problems usually observed in patients subjected to high-dose GC use. Some events are observed more frequently beyond a defined “dose threshold” (exceeding 7.5 mg/day of prednisone), and an increased GC dose demonstrates a stronger correlation with sleep disturbances than a longer duration of use [133,134]. Studies on animals reveal that prolonged prednisolone administration causes a decrease in melatonin secretion and consequentially shortened sleep [135]. Therefore, the lowest effective dose of steroids should be used to avoid complications. 

Synthetic GCs disturb the physiological release of cortisol during the circadian rhythm [62]. Consequently, they should be administered chronologically—with a larger dose given in the morning during the highest physiological cortisol synthesis rate [136]. Interestingly, administering low-dose prednisolone before the physiological circadian increase (at 02:00 a.m.) improves RA symptom control [137]. Modified-release prednisone pills designed for nighttime release meet this demand, releasing prednisone around 2 a.m. when taken at 10 p.m. [138]. Therefore, appropriate education and conscious GC chronotherapy, which involves administering these drugs early in the morning or using modified-release forms in the evening, are critical to increasing effectiveness and minimizing the risk of side effects.

### 5.2. Disease-Modifying Antirheumatic Drugs

Conventional DMARDs, such as methotrexate, sulfasalazine, hydroxychloroquine, mycophenolate mofetil, and azathioprine, conversely to GCs, do not generally cause sleep disorders [139,140,141,142,143]. However, there are some exceptions. A solitary case report mentions the unfavorable indirect effect of sulfasalazine, linked to facial puffiness, that may contribute to sleep apnea syndrome [144]. Insomnia can also develop after chloroquine use in SLE patients [145]. Nevertheless, conventional and biological DMARD treatment usually improves sleep quality by diminishing nocturnal pain through appropriate control of disease activity. For instance, methotrexate may improve sleep disorders in RA patients but not as effectively as etanercept (an anti-TNF-alpha inhibitor) [146]. No adverse effects of JAK-STAT inhibitors, anti-TNF-alpha, IL-6 inhibitors, or rituximab (anti-CD20) on sleep quality were reported [147,148,149,150].

## 6. Prevention and Treatment of Sleep Disorders in Connective Tissue Diseases

Chronic insomnia requiring sedatives is more prevalent in patients with autoimmune conditions than in the general population [151]. Conversely, the use of DMARDs is generally associated with disease control and improved sleep quality in patients with CTDs (Table 2). 

Psychological treatment is one of the methods that can be used to address sleep disorders, applicable not only to patients with rheumatic diseases but also to individuals without such conditions (Table 2). Cognitive behavioral therapy (CBT) has proven highly effective in managing insomnia [152]. The European Sleep Research Society recommends this type of therapy as a first-line treatment, followed by pharmacologic intervention [153]. 

**Table 2:** Treatment of sleep disorders in connective tissue diseases.

Disease (Number of Patients)	Medication/Medical Intervention	Influence on Sleep Disorders	Ref.
Healthy (*n* = 41)	monitored program of ≥150 min of moderate-to-vigorous-intensity physical activity per week for six months	↓ insomnia symptom severity (evaluated using ISI)	[154]
Healthy (*n* = 110)	pilates-based exercise program	↓ depression and anxiety scores	[155]
RA (*n* = 26)	relaxation-based yoga intervention	↑ sleep quality (measured by PSQI score) and FSS questionnaire	[156]
SLE (*n* = 58)	progressive aerobic exercise	↓ the severity of depression and anxiety	[157]
SLE (*n* = 76)	physical activity counseling	safe and feasible for further investigation	[158]
SS (*n* = 59)	16-week resistance exercise program	↓ general fatigue	[159]
Chronic primary insomnia (*n* = 60)	aerobic and resistance exercise trial	no effect on psychological stress, sleep quality, depressive symptoms, or QoL	[160]
SLE (*n* = 50)	16-week digital therapeutic intervention (focused on dietary, environmental, and lifestyle-triggering factors)	↑ sleep quality, vitality, and mental health	[161]
RA (*n* = 50)	a 10-week trial of an anti-inflammatory diet	improved fatigue, pain, mental health, vitality, and subjective perception of disease activity	[162]
SLE (*n* = 23)	A 6-week trial of a low-glycemic-index diet	improved sleep quality	[163]
Chronic inflammatory arthritis (*n* = 121)	1-week whole-body cold mist shower therapy	↓ pro-inflammatory cytokine concentrations	[164]
RA (*n* = 48)	trial of curcumin supplementation for eight weeks	(the aerobic program potentiates both effects)	[165]
RA (*n* = 60)	6-month foot reflexology trial	↑ QoL and ↓ pain and fatigue (assessed FACIT-F, BPI-SF, and LupusQoL)	[166]
Patients with mild knee pain (*n* = 50)	6-month krill oil supplementation	↓ activity and symptoms (DAS28-ESR)	[167]

ISI—insomnia severity index; FSS—fatigue severity scale; QoL—quality of life; PSQI—Pittsburgh Sleep Quality Index; RA—rheumatoid arthritis; SLE—systemic lupus erythematosus; SS—Sjorgen Syndrome; FACIT-F—Functional Assessment of Chronic Illness Therapy—Fatigue; BPI-SF—Brief Pain Inventory—Short Form; LupusQoL—Lupus Quality of Life.

Adequate pharmacologic treatment requires a comprehensive understanding of the various causes of sleep disorders in patients with CTDs. Crucial steps involve diagnosing the specific type of sleep disorder and excluding factors that could impact sleep quality, such as pain. Given the shared pathogenetic pathways between CTDs and sleep disorders, prioritizing the treatment of the underlying CTD is paramount. Table 3 presents the impact of treatment on these disorders.

**Table 3:** The influence of conventional and biological DMARDs on sleep disorders.

Disease (Number of Patients)	DMARD	Influence on Sleep Disorders	Ref.
RA (*n* = 288)	tocilizumab	↓ the severity of sleep disorders ↑ sleep quality (evaluated with PSQI)	[168]
Healthy individuals (*n* = 79)	tocilizumab	Prevention of sleep problem development in healthy individuals	[169]
Ankylosing spondylitis (*n* = 60)	anti-TNF-alpha therapy	↓ in disease activity and fatigue ↑ sleep quality (evaluated with PSQI)	[170]
RA (*n* = 35)	anti-TNF-alpha therapy	↑ sleep quality (PSQI)	[171]
Treatment-resistant major depression with high inflammation (*n* = 36)	infliximab	↑ sleep efficiency	[172]
Healthy (*n* = 16)	anakinra (IL-1 receptor antagonist)	↓ postprandial fatigue (evaluated by Stanford Sleepiness Scale)	[173]

RA—rheumatoid arthritis; PSQI—Pittsburgh Sleep Quality Index; TNF—tumor necrosis factor; IL-1—interleukin 1; IL-6—interleukin 6.

Important cytokines influencing sleep quality include IL-10 and IL-4, which are known to inhibit sleep in the phase of non-rapid eye movement (NREM). Lowering their concentrations could improve sleep quality [174,175]. However, the development of IL-10 signaling inhibitors is still ongoing, posing a challenge to implementing this approach [176]. 

Recent data highlight the potential role of cannabinoids in reducing rheumatic pain, particularly in conditions like fibromyalgia syndrome and rheumatoid arthritis [177]. Some cannabinoids, such as cannabidiol (CBD), cannabigerol (CBG), and delta 9-tetrahydrocannabinol (THC), have demonstrated the ability to reduce TNF, IL-1β, IL-6, and interferon-gamma, thereby improving sleep quality [178]. Although there is insufficient evidence to make recommendations for the use of these drugs in the treatment of sleep disorders, the preliminary results are promising, and their use appears safe if adequately implemented.

Melatonin supplementation holds potential as a beneficial intervention for treating sleep disorders in patients with CTDs without influencing disease activity [58,60,61]. This approach stands out for its favorable safety profile, characterized by markedly low adverse effects [179,180].

### 6.1. Treatment of Coexisting Diseases with Sleep Disorders

As previously acknowledged, adjusting dietary intake to meet energy requirements could have therapeutic effects on sleep [77]. Sleep extension has been shown to reduce energy intake among overweight adults, which can be applied to obesity prevention and weight loss strategies [181]. In addition to lifestyle changes, pharmacological treatment is used in obesity management. Liraglutide, a glucagon-like peptide-1 agonist—GLP-1—may achieve significant weight loss in a relatively short time, causing anti-inflammatory and analgesic effects while treating non-rheumatic diseases [182,183,184]. However, future studies are needed to confirm this hypothesis.

### 6.2. Prevention

**Figure 3:**
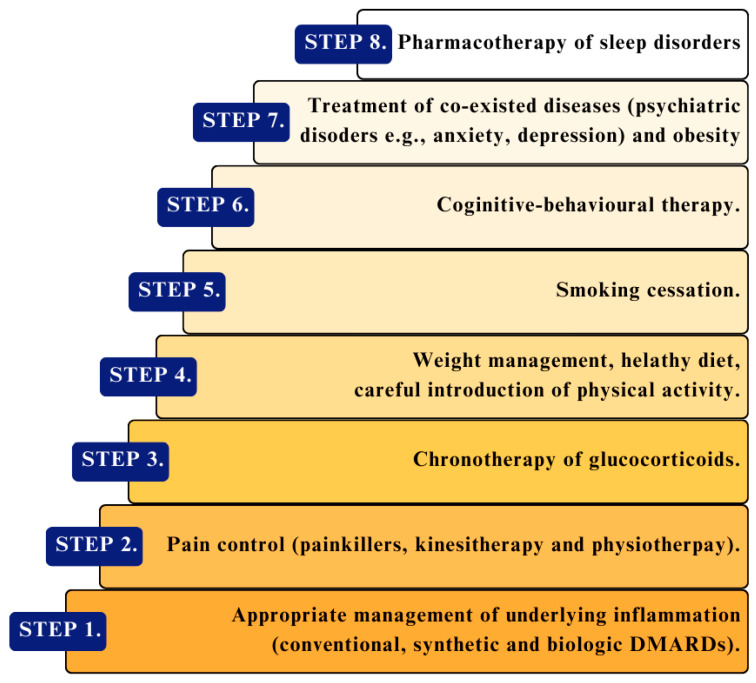
Proposed constitutive steps in the treatment of sleep disorders related to CTDs. DMARDs—disease-modifying antirheumatic drugs.

**Figure 4:**
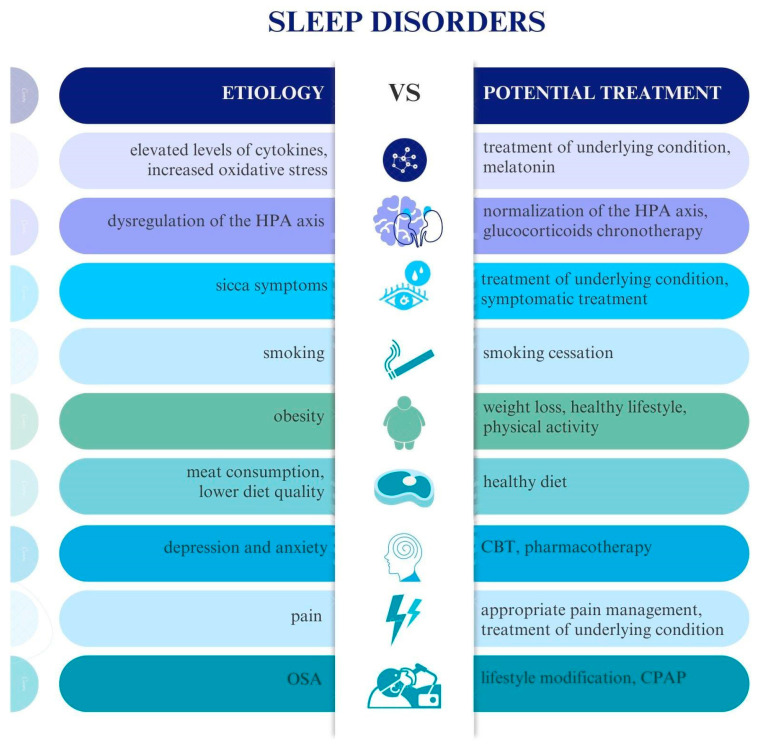
Etiology and treatment strategies for sleep disorders in CTDs. HPA axis—hypothalamus–pituitary–adrenal axis; CBT—cognitive behavioral therapy; OSA—obstructive sleep apnea; CPAP—continuous positive airway pressure.

To summarize, specific steps should be taken to prevent and treat sleep disorders in patients with CTDs (Figure 3 and Figure 4). Preventive measures should include weight control, smoking cessation, engagement in physical activity, early and appropriate treatment of underlying disease (with a focus on anti-inflammatory and pain management), and avoiding high glucocorticoid doses. However, the primary treatment always includes conventional therapy for rheumatic diseases, which decreases pro-inflammatory cytokines participating in the pathophysiological process of disease activity and reduces the severity of sleep disorders. It allows for better pain control, decreases the activity of connective tissue diseases, and prevents sleep problems. If health issues are associated with anxiety or depression, adequate psychotherapy and pharmacotherapy are often necessary. 

## 7. Conclusions

Sleep disorders in patients with rheumatic diseases represent a crucial disease component rather than a separate entity, emphasizing their integral role in the overall well-being and quality of life of affected individuals. These sleep disorders are complex and multifactorial, with causes including pro-inflammatory cytokines (e.g., IL-6, IL-1β, TNF-α, IFN-γ), hormonal imbalances, obesity-related cytokines, dietary factors, melatonin, depression, anxiety, chronic pain, and smoking, along with medications like high-dose glucocorticosteroids. Managing these sleep disorders involves treating the underlying rheumatic condition with new-generation DMARDs, addressing pain, managing glucocorticoid use, addressing mental health issues, promoting weight loss in obese patients, and considering melatonin and non-pharmacologic therapies. Specific sleep disorders may require treatments like CPAP for OSA and dopaminergic drugs for RLS. Severe or chronic sleep disorders should be managed by specialized physicians in clinical centers. This comprehensive and personalized approach aims not only to manage sleep disorders but also to enhance overall well-being and quality of life for patients with rheumatic diseases.

## Supplementary Materials

The following supporting information can be downloaded at: https://www.mdpi.com/article/10.3390/jcm13133656/s1, Table S1: Quality assessment of gathered research.

## Abbreviations

ACTH	adrenocorticotropic hormone
AHEI-10	alternative healthy eating index—2010
CBT	cognitive behavioral therapy
CD20	cluster of differentiation 20
CPAP	continuous positive airway pressure
CRP	C-reactive protein
CSF	cerebrospinal fluid
CTD	connective tissue diseases
DC	dendritic cell
DMARD	disease-modifying antirheumatic drugs
EDS	excessive daytime sleepiness
FACIT-F	functional assessment of chronic illness therapy-fatigue
FSS	fatigue severity scale
GC	glucocorticoids
GLP-1	glucagon-like peptide-1
GM-CSF	granulocyte–macrophage colony-stimulating factor
HLA	human leukocyte antigen
HPA	axis hypothalamic–pituitary–adrenal axis
ICAM-1	intercellular adhesion molecule 1
IFN-γ	interferon gamma
IL	interleukin
IIM	idiopathic inflammatory myopathies
ISI	insomnia severity index
JAK-STAT	janus kinase–signal transducer and activator of transcription
LupusQoL	lupus quality of life
MTX	methotrexate
NO	nitric oxide
NREM	non-rapid eye movement
OSA	obstructive sleep apnea
OSAHS	obstructive sleep apnea/hypopnea syndrome
PM/DM	polymyositis/dermatomyositis
PSQI	Pittsburgh Sleep Quality Index
QoL	quality of life
RA	rheumatoid arthritis
RLS	restless leg syndrome
SAA	serum amyloid protein a
SLE	systemic lupus erythematosus
SSc	systemic sclerosis
SS	Sjögren’s syndrome
TNF-α	tumor necrosis factor alpha
