# Supplementary material for: Sleep Disorders in Connective Tissue Diseases—Coexisting Diseases or Disease Components?
Source: J Clin Med. 2024 Jun 22;13(13):3656. doi: 10.3390/jcm13133656 (PMC11242285; doi:10.3390/jcm13133656)
Supplement: Supplementary file 1 [file jcm-13-03656-s001.zip › jcm-3033351-supplementary.pdf]

Supplementary Table S1. Quality assessment of studies included in the article.

	Research Question	Methodology	Data Analysis	Results	Limitations
Al-Jiffri et Abd El-Kader (2021)	The research question addresses an important gap in understanding the effects of exercise on sleep quality and inflammatory markers in individuals with chronic primary insomnia. It aims to compare the impact of aerobic and resistance exercise training on sleep quality and inflammatory markers, providing valuable insights into non-pharmacological interventions for insomnia management. The question is clear, relevant, and contributes to the knowledge base in the field of sleep medicine and exercise science.	The study employed a randomized controlled trial design, randomly assigning previously sedentary subjects with chronic primary insomnia to either an aerobic exercise intervention group or a resistance exercise intervention group. Polysomnographic recordings and measurements of inflammatory markers (IL-6, IL-10, TNF-α) were conducted before and after six months of exercise training. The methodology appears robust, utilizing appropriate measures and randomization to compare the effects of different exercise modalities on sleep quality and inflammatory markers.	Data analysis involved comparing sleep parameters and inflammatory marker levels before and after the exercise intervention in both groups. Statistical analysis was conducted to determine significant changes within each group and differences between groups. The statistical methods used appear appropriate, providing reliable insights into the effects of aerobic and resistance exercise training on sleep quality and inflammatory markers in individuals with chronic primary insomnia.	The results indicate that both aerobic and resistance exercise training led to improvements in sleep quality and reductions in inflammatory markers among subjects with chronic primary insomnia. However, aerobic exercise training was found to be more effective than resistance exercise training in modulating inflammatory markers and improving sleep quality. The findings are clearly presented and supported by statistical analysis, contributing to our understanding of the benefits of exercise for insomnia management.	While the study provides valuable insights, several limitations should be considered. These include the relatively small sample size, which may limit the generalizability of the findings, and the lack of a control group receiving no exercise intervention. Additionally, the study's duration of six months may not capture long-term effects of exercise on sleep quality and inflammatory markers. Future research with larger sample sizes, longer follow-up periods, and inclusion of control groups is needed to confirm and extend these findings.

Aibar-Almazán et al. (2019)	The research question aims to analyze the effects of a Pilates-based exercise program on sleep quality, anxiety, depression, and fatigue in community-dwelling Spanish postmenopausal women aged 60 and over. It is well-defined and aligns with the objectives of the study. The question addresses important aspects of well-being in this demographic group and provides valuable insights into the potential benefits of Pilates exercise for improving sleep quality and psychological well-being.	The study employs a randomized controlled trial design, which is appropriate for assessing the effects of the Pilates-based exercise program. A total of 110 postmenopausal women were randomly allocated to either a control group or a Pilates group. Outcome measures include sleep quality, anxiety, depression, and fatigue, assessed using validated scales. The methodology appears robust, with clear allocation procedures and appropriate outcome measures to evaluate the impact of the intervention.	Data analysis involves assessing changes in sleep quality, anxiety, depression, and fatigue before and after the Pilates intervention, as well as comparing outcomes between the Pilates and control groups. The results are presented clearly, indicating significant improvements in all domains of sleep quality and reductions in anxiety, depression, and fatigue in the Pilates group. Effect sizes are provided to demonstrate the magnitude of changes. Statistical differences between groups are reported, enhancing the rigor of the analysis. Overall, the data analysis appears thorough and well-	The results demonstrate significant improvements in sleep quality, anxiety, depression, and fatigue following the twelve-week Pilates exercise intervention for community-dwelling Spanish postmenopausal women aged 60 and over. The findings suggest that Pilates exercise has beneficial effects on various aspects of well-being in this demographic group. The results are presented clearly and supported by statistical analyses, contributing to the understanding of the potential benefits of Pilates for older women's health.	Some limitations should be considered, such as the lack of long-term follow-up to assess the sustainability of the observed improvements. Additionally, the study may have benefited from a larger sample size to enhance generalizability. The reliance on self-reported measures could introduce bias, and potential confounding variables may not have been fully controlled. Despite these limitations, the study provides valuable insights into the potential benefits of Pilates exercise for improving sleep quality, anxiety,

			conducted.		depression, and fatigue in postmenopausal women, highlighting areas for future research.
Albrecht et al. (2020)	The research question is clear and relevant, focusing on the long-term trends in outcomes for patients with idiopathic inflammatory myopathies (IIM). This is important for understanding how treatments and patient experiences have evolved over time.	The methodology is robust, utilizing cross-sectional data from a large national database over a substantial period (1997-2017). This provides a comprehensive overview of trends. However, the methodology may be limited by its reliance on annual data reporting and potential changes in data collection methods over the years.	The use of descriptive statistics and the Cochrane-Armitage test for trend analysis is appropriate for identifying trends over time. Comparisons across years and the inclusion of both patient-reported and physician-reported outcomes provide a well-rounded analysis. Some advanced statistical methods could enhance the analysis further.	The results indicate significant improvements in various patient-reported outcomes, which is a positive finding. The decrease in glucocorticoid use and other clinical improvements are well-documented. However, it is important to consider potential biases and changes in disease duration when interpreting these results.	The study acknowledges key limitations, such as the decrease in patient numbers due to a switch to electronic documentation and potential biases from changes in disease duration. These limitations are significant but well-acknowledged, showing transparency in the research. Limited data on specific autoantibodies and organ manifestations could affect the comprehensiveness of

					the study.
Alexopoulos et al. (2013)	The research question aims to investigate the association between obstructive sleep apnea (OSA) severity, excessive daytime sleepiness (EDS), and plasma TNF-α levels in children. Specifically, the study hypothesizes that higher OSA severity is associated with increased frequency of EDS and elevated TNF-α levels, and that high TNF-α levels predict the presence of EDS. These hypotheses are clearly articulated and relevant to understanding the pathophysiology of EDS in pediatric OSA.	The methodology involves conducting polysomnography in children without and with snoring to assess OSA severity, determining EDS through parental responses to specific questions, and measuring plasma TNF-α levels. The inclusion of age, gender, and obesity as covariates in the analysis strengthens the study design. However, the study's reliance on parental reports for EDS assessment may introduce subjectivity and potential bias.	The data analysis appears robust, with statistical comparisons made between different groups to assess the associations between OSA severity, EDS frequency, and TNF-α levels. Adjustments for potential confounders, such as age, gender, and obesity, enhance the reliability of the results. However, the lack of significant differences in TNF-α levels between groups suggests the need for further exploration of potential mediators of the OSA-EDS association in children.	The results indicate that children with moderate-to-severe OSA are at a significantly higher risk for EDS compared to controls, but there are no significant differences in plasma TNF-α levels between groups. Additionally, high TNF-α levels do not predict the presence of EDS in children with sleep apnea. These findings suggest that while OSA severity is associated with EDS frequency in children, plasma TNF-α levels may not be a reliable predictor of EDS in this population.	One limitation of the study is the reliance on parental reports for EDS assessment, which may introduce subjectivity and potential bias. Additionally, the study's small sample size and cross-sectional design limit the generalizability of the findings and preclude the establishment of causality. Further longitudinal studies with larger sample sizes and objective measures of EDS are needed to validate the study's findings and elucidate the underlying mechanisms linking OSA severity and EDS in children.
Alfano et al. (2007)	The research question is clearly defined: to	The methodology involves examining	The data analysis appears appropriate for	The results are presented clearly,	The study acknowledges several

examine sleep-related problems (SRPs) among youth with anxiety disorders and explore associations with various factors such as age, gender, type of anxiety disorder, anxiety severity, and functional impairment. Additionally, the impact of pharmacological treatment (fluvoxamine versus pill placebo) in reducing SRPs is assessed. The question addresses an important gap in understanding the prevalence and correlates of SRPs in youth with anxiety disorders, with implications for treatment and clinical management.

clinician and parent reports of SRPs among a large sample of children and adolescents (ages 6 to 17 years) with anxiety disorders, before and after treatment, as part of a double-blind, randomized, controlled trial. The study design allows for the comprehensive assessment of SRPs and their associations with various factors, as well as the evaluation of treatment effects. However, reliance on clinician and parent reports may introduce bias, and the use of a placebo-controlled trial design enhances methodological rigor. Further details on participant recruitment and assessment procedures would enhance transparency.

the study objectives, utilizing descriptive statistics and inferential tests to examine the frequency of SRPs and their associations with demographic and clinical variables. Statistical tests are used to compare SRPs between treatment groups and assess changes over time. The findings demonstrate significant associations between SRPs, anxiety severity, and functional impairment, as well as treatment effects of fluvoxamine. However, more information on statistical assumptions and sensitivity analyses would strengthen the analysis.

indicating that a majority of youth with anxiety disorders experienced SRPs, with a positive association between total SRPs, anxiety severity, and interference in family functioning. Furthermore, children treated with fluvoxamine showed significantly greater reductions in SRPs compared to those receiving placebo. These findings highlight the importance of assessing and addressing SRPs in youth with anxiety disorders, both in research and clinical practice. However, further exploration of potential moderators and longitudinal effects would provide additional insights.

limitations, including the reliance on subjective reports of SRPs from clinicians and parents, potential biases associated with placebo-controlled trial designs, and the generalizability of findings to specific age groups and anxiety disorders. Additionally, the study does not address potential confounding variables comprehensively, and the impact of other treatment modalities (e.g., psychotherapy) on SRPs is not assessed. Further discussion on these limitations and their implications for interpretation would strengthen the discussion section.

Almendros et al. (2016)	The research question explores whether chronic intermittent hypoxia (IH), a characteristic feature of obstructive sleep apnea (OSA), leads to the release of tumor-promoting exosomes in the circulation. The question is clear, relevant, and addresses an important gap in understanding the link between OSA and cancer progression.	The methodology involves a well-designed experimental study using C57/B6 male mice exposed to 6 weeks of IH or room air (RA), with a subgroup injected with TC1 lung carcinoma cells. Exosomes from both mouse plasma and human patients with OSA before and after treatment were cocultured with tumor cells to assess malignant properties. Various assays were employed to evaluate proliferation, migration, invasion, endothelial monolayer disruption, and molecular effects on target genes. Overall, the methodology is robust and appropriate for investigating the research question.	The data analysis effectively compares the effects of IH-induced exosomes from both tumor-bearing and non-tumor-bearing mice on tumor cell properties, as well as the impact of OSA patient-derived exosomes on human adenocarcinoma cells. Molecular analyses identify distinct miRNAs and their gene targets, providing valuable insights into the mechanisms underlying the observed effects. The presentation of results is clear and supports the study's conclusions.	The results demonstrate that exosomes released under IH conditions enhance specific malignant properties of tumor cells in both mouse and human models. IH-induced exosomes promote proliferation and migration of tumor cells, while distinct miRNAs and their gene targets are identified. These findings suggest a mechanistic link between OSA-related hypoxia and tumor aggressiveness mediated by circulating exosomes.	While the study provides compelling evidence of the tumor-promoting effects of IH-induced exosomes, several limitations should be considered. The use of animal models may not fully reflect human pathophysiology, and the small sample size of human patients limits generalizability. Additionally, the study focuses on exosome-mediated effects on tumor cells in vitro, and the translation of these findings to clinical outcomes in OSA patients requires further investigation. Addressing these limitations could strengthen the study's findings and implications.

AlRyalat et al. (2021)	The research question is clearly defined and relevant, addressing an important gap in the literature.	The methodology is well-described, utilizing data from a randomized trial and including three distinct groups based on nicotine concentration levels.	The data analysis appears robust, comparing sleep outcomes among the three nicotine concentration groups at baseline and after 6 weeks of consumption.	The results are clearly presented, highlighting the significant association between higher nicotine doses and worsened sleep quality after 6 weeks of consumption.	While the study contributes valuable insights, limitations include reliance on self-reported sleep measures, potential for confounding, and limited generalizability.
Alt et al. (2014)	The study effectively addresses the research question by investigating the association between cytokine gene expression and sleep quality in patients with CRS, providing valuable insights into sickness behavior in CRS. The hypothesis is supported by the findings, demonstrating correlations between cytokine expression and sleep disturbances. Overall, the research question is clearly defined and relevant to understanding the	The methodology is comprehensive and well-described, utilizing a prospective, translational research cohort design. Inclusion criteria, data collection procedures, and analytical techniques are clearly outlined. The use of validated survey instruments and molecular analysis enhances the validity of the study. However, the small sample size and potential confounding variables related to prior medical treatment represent limitations. Overall, the methodology is robust but acknowledges	The data analysis employs appropriate statistical methods, including descriptive statistics and correlation analysis. The correlations between variables are explored to elucidate relationships between cytokine gene expression, sleep quality, QOL measures, and clinical disease severity. However, the small sample size and lack of correction for multiple comparisons are potential limitations that could affect the robustness of the results. Despite these	The results section presents key findings related to sleep quality, QOL measures, clinical disease severity, and cytokine gene expression in patients with CRS. The findings support the hypothesis, demonstrating associations between cytokine expression and sleep disturbances. However, the interpretation is cautious, considering the limitations of the study, such as the small sample size and potential confounding variables. Overall, the results contribute to	The study acknowledges several limitations, including the small sample size, potential confounding variables related to prior medical treatment, and the lack of correction for multiple comparisons. These limitations could affect the generalizability and robustness of the findings. Additionally, the cross-sectional design limits the ability to infer causality. Despite these limitations, the study provides valuable insights into

	pathophysiology of CRS.	important limitations.	limitations, the data analysis is conducted rigorously and provides valuable insights into the research question.	understanding the role of cytokines in CRS-related sickness behavior.	the research question and highlights areas for future research.
Alten et al. (2010)	The research question aims to investigate the effects of long-term low-dose chronotherapy with modified-release (MR) prednisone on the hypothalamus-pituitary-adrenal (HPA) axis in patients with rheumatoid arthritis (RA). Specifically, the study assesses cortisol plasma concentrations in response to corticotropin-releasing hormone (CRH) testing at different timepoints during treatment with immediate-release (IR) and MR prednisone. The question is clear, focused, and relevant to understanding the	The methodology involves a prospective study design as part of the Circadian Administration of Prednisone in Rheumatoid Arthritis (CAPRA-1) study. Twenty-eight patients with RA were included and underwent CRH testing at three timepoints: at baseline on prestudy IR prednisone, after a 3-month double-blind phase on either IR or MR prednisone, and after a 9-month open-label extension on MR prednisone. Cortisol plasma concentrations were assessed, and changes were compared to individual patients'	The data analysis involves assessing cortisol plasma concentrations in response to CRH testing at different timepoints during treatment with IR and MR prednisone. Changes in cortisol levels are reported and compared between treatment groups. Descriptive statistics, such as means and standard deviations, are used to summarize the data. Additionally, the study evaluates the number of normal, suppressed, and no response reactions to CRH testing across treatments. The analysis appears	The results indicate that treatment with nighttime-release prednisone, either IR or MR formulation, did not significantly change adrenocortical function over 12 months in patients with RA. Cortisol plasma concentrations in response to CRH testing were similar between IR and MR prednisone treatments at different timepoints. Additionally, switching from IR to MR prednisone did not influence cortisol responses, and long-term treatment with MR prednisone did not worsen adrenal impairment. These	The abstract acknowledges several limitations, including the small sample size and the absence of a control group for comparison. The study also lacks long-term follow-up beyond 12 months, limiting the assessment of the sustained effects of MR prednisone on HPA axis function. Additionally, the study does not explore potential confounding factors or comorbidities that may influence cortisol responses. Furthermore, the use of CRH testing as the sole measure of HPA axis function may not

	impact of chronotherapy on HPA axis function in RA patients. By addressing this question, the study provides valuable insights into the safety and efficacy of MR prednisone in RA management. Overall, the research question is appropriate and aligned with the study objectives.	efficacy and safety data. The study design allows for longitudinal assessment of HPA axis function in response to different prednisone formulations. However, the sample size is relatively small, and the study lacks a control group for comparison. Overall, the methodology is suitable for investigating the research question, but larger studies with control groups may enhance the robustness of the findings.	appropriate for the research question and provides insights into the effects of MR prednisone on HPA axis function in RA patients. However, more detailed reporting of statistical methods and effect sizes would enhance the transparency of the analysis. Overall, the data analysis is suitable for addressing the study objectives.	findings suggest that nighttime-release prednisone may not adversely affect HPA axis function in RA patients. Overall, the results effectively address the research question and provide valuable insights into the safety of MR prednisone in RA management.	capture all aspects of adrenal function. Despite these limitations, the study provides valuable insights into the short-term effects of MR prednisone on the HPA axis in RA patients. However, larger studies with longer follow-up periods and comprehensive assessments of adrenal function are needed to confirm these findings and address potential confounders. Overall, the limitations are appropriately acknowledged and discussed in the context of the study findings.
Andersen et al. (2016)	The research question is clearly defined and				The study acknowledges several

relevant, aiming to evaluate the adverse effects and safety profile of exogenous melatonin in humans. This addresses an important aspect of melatonin use, considering its widespread application in various medical and surgical conditions. The question is comprehensive, covering both short-term and long-term use of melatonin and highlighting specific patient groups where risks may differ.

The methodology involves a review of existing literature on the adverse effects and safety of exogenous melatonin in humans. While specific details on search criteria and inclusion/exclusion criteria are not provided, the review approach is appropriate for synthesizing available evidence on this topic. However, without transparency regarding the literature search and selection process, the rigor of the methodology is somewhat limited.

As this is a review article, data analysis is primarily qualitative, involving the synthesis and evaluation of findings from existing studies on melatonin safety. The reported adverse effects and safety profile of exogenous melatonin are summarized based on evidence from animal and human studies, as well as randomized clinical trials. While no statistical analyses are conducted, the assessment of safety outcomes is thorough and provides valuable insights for clinicians and researchers.

The results suggest that short-term use of exogenous melatonin is generally safe, with only mild adverse effects reported, such as dizziness, headache, nausea, and sleepiness. Moreover, long-term use appears to have minimal adverse effects, comparable to placebo, based on randomized clinical studies. However, the long-term safety of melatonin in children and adolescents requires further investigation, and caution is advised for pregnant and breastfeeding women due to a lack of human studies. The findings provide important guidance on the safety profile of melatonin in

limitations, including the need for further investigation into the long-term safety of melatonin in specific populations, such as children, adolescents, pregnant women, and breastfeeding women. Additionally, the review methodology may introduce bias due to the reliance on existing literature, which may vary in quality and study design. The lack of transparency regarding the literature search and selection process is also a limitation, affecting the reproducibility and reliability of the findings.

				various patient groups.	
Arvidson et al. (1997)	The research question aims to investigate the effect of timing of prednisolone administration on the diurnal rheumatoid inflammatory process. Specifically, the study seeks to determine whether administering low doses of prednisolone at 2:00 am or 7:30 am has differential effects on rheumatoid arthritis symptoms and inflammatory markers. The question is clear, focused, and relevant to understanding the	The methodology involves a randomized controlled trial design with 26 patients with rheumatoid arthritis randomly divided into two equal groups. Patients were allocated to receive low doses of prednisolone either at 2:00 am or 7:30 am. Assessments were conducted at 7:30 am both at the start of the study (day 1) and after four doses of prednisolone (day 5). The study protocol ensured differences in the time period from the	The data analysis involves comparing the effects of prednisolone administration at different times (2:00 am vs. 7:30 am) on rheumatoid arthritis symptoms and inflammatory markers. Changes in outcome measures, including morning stiffness, joint pain, inflammatory indices, and serum concentrations of IL-6, CRP, serum amyloid protein A, and erythrocyte sedimentation rate, are reported for both study	The results indicate that administering low doses of prednisolone at 2:00 am had favorable effects on morning stiffness, joint pain, Lansbury index, Ritchie index, and morning serum concentrations of IL-6 compared to administration at 7:30 am. Additionally, the 2:00 am group showed modest improvements in circulating concentrations of CRP, serum amyloid protein A, and erythrocyte sedimentation rate. In	The abstract acknowledges several limitations, including the small sample size and the lack of longer-term follow-up beyond day 5. Additionally, the study did not assess patient-reported outcomes or functional status, which could provide a more comprehensive evaluation of treatment effects. Furthermore, the study did not explore potential confounding factors or

potential impact of timing protocols on glucocorticoid efficacy in rheumatoid arthritis management. By addressing this question, the study provides valuable insights into optimizing glucocorticoid therapy for rheumatoid arthritis patients. Overall, the research question is appropriate and aligned with the study objectives.

last dose of prednisolone to assessment, with a shorter interval in the 2:00 am group compared to the 7:30 am group. Outcome measures included morning stiffness, joint pain, inflammatory indices, and serum concentrations of IL-6, CRP, serum amyloid protein A, and erythrocyte sedimentation rate. The methodology is appropriate for investigating the research question and allows for the comparison of prednisolone timing effects on rheumatoid arthritis symptoms and inflammatory markers. However, the sample size is relatively small, and longer-term follow-up would strengthen the study's findings. Overall, the methodology is suitable for addressing

groups. Statistical significance is assessed using appropriate tests (e.g., t-tests) with significance levels reported. The analysis appears appropriate for the research question, providing insights into the differential effects of prednisolone timing on rheumatoid arthritis outcomes. However, more detailed reporting of statistical methods and effect sizes would enhance the transparency of the analysis. Overall, the data analysis is suitable for addressing the study objectives.

contrast, the 7:30 am group exhibited minor effects on morning stiffness and IL-6 concentrations. These findings suggest that prednisolone administration preceding the circadian flare in inflammatory activity may improve rheumatoid arthritis symptoms and reduce IL-6 synthesis. Overall, the results effectively address the research question and provide valuable insights into optimizing glucocorticoid therapy timing for rheumatoid arthritis management.

comorbidities that may influence treatment response. The short duration of the study may limit the generalizability of the findings to long-term glucocorticoid therapy. Despite these limitations, the study provides valuable insights into the effects of prednisolone timing on rheumatoid arthritis outcomes. However, larger studies with longer follow-up periods and comprehensive assessments of patient outcomes are needed to confirm these findings and address potential confounders. Overall, the limitations are appropriately acknowledged and discussed in the context of the study findings.

		the research question, but larger studies with extended follow-up periods may enhance the robustness of the results.			
Auvinen et al. (2018)	The study investigates the prevalence of restless legs symptoms (RLS) in patients with depressive symptoms compared to controls without psychiatric diagnosis, and examines the association between RLS and TNF-α levels. This question is relevant for understanding the link between sensorimotor disorders and mental health conditions.	A cross-sectional study was conducted with 706 patients with depressive symptoms and 426 controls. Depressive symptoms were evaluated using the Beck Depression Inventory, and psychiatric diagnoses were confirmed using the Mini-International Neuropsychiatric Interview. The methodology is appropriate for the research question, utilizing validated tools and a large sample size.	The analysis showed that the prevalence of RLS symptoms was significantly higher in patients with clinical depression (50%) and depressive symptoms (42.4%) compared to controls (24.8%). Elevated TNF-α levels were associated with RLS symptoms in patients with depressive symptoms, both with and without a clinical depression diagnosis. CRP levels were also higher in clinically depressed patients. The statistical methods are sound and support the conclusions drawn.	The results indicate a higher prevalence of RLS symptoms among patients with depressive symptoms, with significant associations between elevated TNF-α levels and RLS symptoms in these patients. The findings suggest that TNF-α could be a mediating factor linking RLS and depressive symptoms, highlighting the role of inflammation in the comorbidity of these conditions.	The cross-sectional design limits causal inferences, and the reliance on self-reported measures for RLS symptoms could introduce bias. The study also does not account for potential confounders such as medication use or other medical conditions. Longitudinal studies are needed to confirm these associations and explore causal mechanisms.
Bakir et al. (2018)	The research question	The study employed a	The data analysis	The results	The study has several

	is clearly defined and relevant: to examine the effect of foot reflexology on pain and sleep quality in rheumatoid arthritis (RA) patients. This question addresses an important aspect of patient care, considering the prevalence of pain and sleep disturbances in RA patients, and explores a non-pharmacological intervention that could offer relief and improve quality of life.	randomized controlled trial (RCT) design, which is robust and minimizes bias. The setting was a rheumatology follow-up polyclinic in Turkey, and the study period was from January to July 2015. Sixty patients were included, and data were collected using a sociodemographic data form, the Pittsburgh Sleep Quality Index (PSQI), and the Visual Analogue Scale (VAS). Foot reflexology was administered to the experimental group. This methodology is appropriate for addressing the research question.	compared pain scores and sleep quality between the experimental and control groups. Statistical significance was determined ($p < .01$), indicating that foot reflexology significantly reduced pain and improved sleep quality. The use of the VAS and PSQI provided quantifiable measures of pain and sleep quality, respectively, allowing for objective comparison between groups.	demonstrated that foot reflexology significantly reduced pain and improved sleep quality in the experimental group compared to the control group. The average pain scores were reduced, and the PSQI scores indicated improved sleep quality over the six weeks of intervention. These findings support the efficacy of foot reflexology as a beneficial non-pharmacological intervention for RA patients.	limitations. The sample size is relatively small, which may limit the generalizability of the findings. The study was conducted in a single clinical setting in Turkey, which may affect the applicability of the results to other populations and settings. Additionally, the study period was only six weeks, which may not capture the long-term effects of foot reflexology on pain and sleep quality. Further research with larger, more diverse populations and longer follow-up periods is needed.
Bang et al. (2012)	The research question investigates the effect	The methodology involves dividing mice	Data analysis includes comparisons of Cry1	The results demonstrate that	Limitations include the use of a mouse model,

	of melatonin on the expression of circadian clock genes in mouse anti-type II collagen antibody-induced arthritis (CIA), addressing an important gap in understanding the role of circadian rhythm in arthritis pathogenesis. However, the study primarily focuses on the role of melatonin and does not explore other potential factors contributing to arthritis development and progression.	into control, CIA, and CIA + melatonin treatment (MLT) groups and assessing mRNA and protein levels of the circadian clock gene Cry1. Paw thickness, histological changes, X-ray assessment, and antibody concentrations are also measured. The experimental design is appropriate for studying the effects of melatonin on arthritis development and provides comprehensive data on various outcome measures.	expression, paw thickness, histological changes, X-ray findings, and antibody concentrations between the different treatment groups. Statistical significance is reported for differences observed, enhancing the reliability of the results. However, additional analyses such as correlation studies or pathway analyses could provide further insights into the mechanisms underlying melatonin-induced arthritis aggravation.	melatonin treatment decreases Cry1 expression and exacerbates arthritis symptoms in mice CIA models, including increased paw thickness, synovial hyperplasia, and destruction of articular cartilage and bone. Additionally, higher concentrations of anti-type II collagen antibodies, TNF-α, and IL-6 are observed in the CIA + MLT group, indicating increased inflammatory response. These findings suggest a potential role of Cry1 in mediating the aggravating effects of melatonin on arthritis.	which may not fully reflect human arthritis pathophysiology. The study primarily focuses on the effects of melatonin without considering potential interactions with other factors or treatments. Additionally, the mechanisms underlying the observed effects on Cry1 expression and arthritis aggravation remain unclear and warrant further investigation. Further studies in human cohorts are needed to validate these findings and assess their clinical relevance.
Bårdsen et al. (	The research question addresses an important gap in understanding the mechanisms underlying fatigue in primary Sjögren's syndrome (pSS) by	The methodology involves measuring Hcrt1, interleukin-1 receptor antagonist (IL-1Ra), IL-1 receptor type 2 (IL-1RII), IL-6, and S100B protein in cerebrospinal	The data analysis employs univariate and multiple regression analyses, as well as principal component analysis (PCA), to explore the	The results elucidate a functional network involving IL-1 β-related molecules and Hcrt1 in fatigue generation in pSS. IL-1 β-related molecules, depression,	The study acknowledges limitations such as the relatively small sample size and the cross-sectional design, which precludes

	investigating the influence of IL-1 β-related molecules and hypocretin-1 (Hcrt1) on fatigue. The study aims to elucidate the complex interplay between inflammatory mediators and neuromodulators in fatigue generation, contributing to the understanding of fatigue pathophysiology in pSS and potentially informing future therapeutic interventions.	fluid (CSF) from 49 pSS patients using enzyme-linked immunosorbent assay (ELISA) and radioimmunoassay (RIA). Fatigue is assessed using the fatigue visual analog scale (fVAS). Regression analyses and principal component analysis (PCA) are utilized to explore the associations between these variables and fatigue. The use of CSF biomarkers and advanced statistical techniques strengthens the methodological approach, although a larger sample size would enhance statistical power.	relationships between IL-1 β-related molecules, Hcrt1, and fatigue. The analyses reveal significant associations between depression, pain, IL-1Ra, and fatigue, highlighting the multifactorial nature of fatigue in pSS. PCA identifies distinct components reflecting IL-1 β-related activity and Hcrt1 levels, providing insights into the underlying mechanisms of fatigue. The incorporation of advanced statistical techniques enhances the interpretation of results.	and pain demonstrate significant associations with fatigue, indicating the involvement of inflammatory pathways and clinical factors. PCA reveals distinct components reflecting IL-1 β-related activity and Hcrt1 levels, suggesting a complex interplay between inflammation and neuromodulation in fatigue pathophysiology. The findings contribute to understanding the multifactorial nature of fatigue in pSS.	causal inferences. Additionally, the use of CSF biomarkers may not fully capture systemic inflammatory processes, and the assessment of Hcrt1 levels in CSF alone may not reflect its central activity accurately. Further longitudinal studies with larger sample sizes and comprehensive assessments of inflammatory and neuromodulatory pathways are warranted to confirm the findings and elucidate the mechanistic underpinnings of fatigue in pSS.
Bassel et al. (2011)	The research question is clear and pertinent,	The methodology is comprehensive, utilizing	Data analysis is thorough and	The results are significant and well-	The study acknowledges

	focusing on identifying the frequency and impact of various symptoms experienced by patients with systemic sclerosis (SSc). This study aims to fill gaps in understanding how these symptoms affect daily functioning, which is crucial for patient-centered care.	a large, national sample of SSc patients. The survey includes a wide range of symptoms, and both frequency and impact are assessed using validated questionnaires. The use of a web-based and paper survey increases accessibility and participation.	appropriate. Symptoms are dichotomized for clarity, and the analysis includes both frequency and impact on daily activities. Descriptive statistics are used to highlight the most common and impactful symptoms, providing a clear picture of patient experiences.	presented. The study identifies key symptoms that frequently affect patients and have a substantial impact on their daily lives, such as fatigue, Raynaud's phenomenon, hand stiffness, joint pain, and difficulty sleeping. These findings are supported by detailed statistical data.	limitations such as potential selection bias due to the survey's voluntary nature and the reliance on self-reported data, which could introduce recall bias. Additionally, the cross-sectional design limits causal inferences. Despite these limitations, the study provides valuable insights into the impact of SSc symptoms on daily life.
Bazzichi et al. (2019)	The research question is clearly defined, aiming to assess the efficacy and safety of weekly subcutaneous tocilizumab (TCZ-SC) 162 mg, alone or with a conventional synthetic DMARD (csDMARD), in moderate-to-severe RA patients with inadequate response to DMARDs or anti-TNFα drugs. This question is	The methodology involves a national, multicenter, open-label, phase IIIb trial conducted in 43 Italian centers as part of the TOZURA umbrella study. Patients were treated for 52 weeks followed by 8 weeks drug-free to evaluate immunogenicity. The	The data analysis likely involved descriptive statistics to characterize the patient population, including demographics and baseline characteristics. Changes in clinical disease activity and other efficacy variables over time	The results demonstrate that TCZ-SC, alone or with a csDMARD, led to a significant reduction in Clinical Disease Activity Index (CDAI) from baseline at week 2, which further progressed up to week 24 and remained stable	The study may have limitations inherent to its open-label design and lack of a placebo or active comparator group, which could introduce bias and limit the ability to draw causal inferences about the efficacy and safety of TCZ-SC. Additionally, the study population consisted of patients

important for informing clinical practice regarding the optimal treatment approach for patients with rheumatoid arthritis (RA) who have failed previous therapies. The study addresses a relevant gap in the literature by evaluating the real-world effectiveness and safety of TCZ-SC in this patient population.

primary endpoint was the change in Clinical Disease Activity Index (CDAI) from baseline at weeks 2 and 24. Other efficacy parameters, including sleep quality, safety, and immunogenicity, were also assessed up to week 52. The study design allows for the evaluation of both short-term and long-term outcomes of TCZ-SC treatment in RA patients, incorporating real-world clinical practice aspects.

were assessed using appropriate statistical methods, such as repeated measures analysis of variance (ANOVA) or non-parametric tests. Safety outcomes and immunogenicity were also analyzed descriptively. The statistical significance of treatment effects was determined based on pre-specified endpoints and statistical thresholds. The analysis appears comprehensive and suitable for evaluating the efficacy, safety, and immunogenicity of TCZ-SC in the study population.

thereafter. Additionally, rapid and sustained improvements were observed in other efficacy variables. The study also found low immunogenicity and no unexpected toxicities associated with TCZ-SC treatment. The findings suggest that TCZ-SC is effective and well-tolerated in RA patients with inadequate response to DMARDs or anti-TNFα drugs, supporting its use in clinical practice. Home administration of TCZ-SC appears feasible, enhancing patient convenience and adherence to treatment. Overall, the results provide valuable insights into the real-world effectiveness and

from Italian centers, which may limit the generalizability of the findings to other populations or healthcare settings. Furthermore, the relatively short follow-up period of 52 weeks may not capture long-term outcomes or potential late-onset toxicities associated with TCZ-SC treatment. These limitations should be considered when interpreting the results and extrapolating them to broader clinical practice contexts.

				safety of TCZ-SC in this patient population.	
Behboudi et al. (2021)	The research question aims to investigate the association between TNF-α-308G/A gene polymorphism, circulating TNF-α levels, and excessive daytime sleepiness (EDS) in patients with coronary artery disease (CAD) and obstructive sleep apnea (OSA). The question is relevant and addresses the need to understand molecular differences	The methodology involves a secondary analysis of the RICCADSA trial, including 326 participants categorized based on OSA presence and EDS status. TNF-α alleles and genotypes are assessed, along with circulating TNF-α levels, oxygen desaturation index, and EDS risk. Multivariate analysis is used to examine associations, adjusting	The data analysis appears rigorous, with multivariate analysis used to explore associations between TNF-α genotypes, circulating TNF-α levels, and EDS risk. Adjustments for confounding factors enhance the reliability of the results. However, specific details about the statistical methods employed are not provided, limiting the	The results indicate no significant differences in TNF-α alleles and genotypes between CAD patients with and without OSA. However, associations are observed between oxygen desaturation index, TNF-α genotypes, and circulating TNF-α levels. Importantly, the TNF-α-308A allele is linked to reduced EDS risk independently of	One limitation of the study is its reliance on a secondary analysis of existing trial data, which may introduce biases and limit the generalizability of the findings. Additionally, the study does not address potential confounding factors, such as medication use or comorbidities, which could influence the associations observed. Further

	in OSA phenotypes in cardiac patients.	for potential confounders. Overall, the methodology is comprehensive and appropriate for addressing the research question.	assessment of the analysis approach.	other factors. These findings provide valuable insights into the role of TNF-α gene polymorphism in modulating TNF-α levels and mitigating EDS in CAD patients with OSA.	research with larger, more diverse cohorts and longitudinal designs is needed to confirm the findings and elucidate underlying mechanisms.
Berenbaum et al. (2021)	The research question is clearly defined and addresses an important issue in osteoarthritis (OA) pathophysiology. The study aims to evaluate the anti-inflammatory and anti-catabolic effects of Liraglutide in in vitro models relevant to OA, which is relevant given the inflammatory nature of OA and the need for novel therapeutic interventions.	The methodology is well-described and appropriate for the research question. The use of two in vitro models, including LPS-stimulated murine Raw 264.7 macrophages and IL-1β-stimulated mouse articular chondrocytes, allows for comprehensive evaluation of the effects of Liraglutide on inflammation and cartilage degradation. Various assays and RT-qPCR analyses were employed to measure relevant parameters, ensuring robustness in data collection.	The data analysis appears thorough and appropriate. Concentration-dependent responses to Liraglutide treatment were assessed, and appropriate statistical methods were likely used to determine significance. The dose-response curves provided valuable insights into the efficacy of Liraglutide in inhibiting inflammatory mediators and cartilage degradation markers in both macrophages and chondrocytes.	The results are clearly presented and support the study's objectives. Liraglutide demonstrated dose-dependent inhibition of inflammatory mediators such as NO, PGE2, IL-6, and TNF in macrophages, as well as IL-6, MMP-3, and GAG in chondrocytes. The shift in M1/M2 macrophage phenotype and the rescue of chondrocyte differentiation markers by Liraglutide further validate its potential as a therapeutic agent for OA.	While the study provides valuable insights, it also has limitations. Being an in vitro study, the findings may not fully translate to in vivo conditions. Additionally, the study primarily focuses on cellular responses, and further research, including in vivo studies and clinical trials, is needed to validate the efficacy and safety of Liraglutide as a treatment for OA in humans.

Bhake et al. (2019)	The study aims to evaluate free cortisol profiles using microdialysis in healthy individuals, examining the circadian rhythm of cortisol secretion and its variations throughout the day, particularly in response to daily activities and meals.	The study employs a robust methodology involving two separate experiments: one measuring total and subcutaneous (SC) free cortisol levels at 10-minute intervals for 24 hours, and the other measuring SC free cortisol levels at 20-minute intervals for 72 consecutive hours in free-living individuals. The use of a portable collection device allows for ambulatory monitoring, capturing cortisol profiles in real-life settings. This methodology enhances ecological validity and provides valuable insights into cortisol dynamics in everyday life.	Data analysis involves measuring total and SC free cortisol levels at frequent intervals and examining the circadian rhythm of cortisol secretion. Statistical analyses likely include calculating mean cortisol levels, assessing peak and trough levels, and examining variations in response to activities and meals. Comparisons between individuals and within individuals across different time points provide insights into cortisol regulation under physiological conditions.	The study findings demonstrate the characteristic circadian rhythm of cortisol secretion, with lowest levels around sleep onset and peak levels around waking, consistent across all participants. Cortisol levels also show increases after lunch consumption. Additionally, cortisol levels remain consistent over 72 hours, suggesting stability in cortisol regulation despite variations in activities. These results highlight the robustness and reliability of cortisol dynamics in healthy individuals.	The study concludes that continuous monitoring of SC free cortisol profiles in healthy individuals is feasible using microdialysis, providing valuable insights into circadian cortisol rhythms and responses to daily activities and meals. The findings pave the way for ambulatory monitoring of cortisol profiles, offering opportunities for studying cortisol dynamics in various physiological and pathological conditions involving the hypothalamic-pituitary-adrenal axis.
Bjorvatn et al. (2021)	The research question aims to investigate the prevalence of RLS	The methodology involves a questionnaire survey conducted at GP	Data analysis includes chi-squared tests and logistic regression to	Results indicate a higher prevalence of RLS (14.3%) among GP	Limitations include reliance on self-reported data without

	among patients consulting their GPs, the severity of symptoms, medication usage, and associations with other common complaints (IBS, CF, CMBP). The question is clearly defined and relevant to clinical practice and patient outcomes.	offices in Southern and Western Norway, ensuring a high response rate (86.8%) and a large sample size (2,634 participants). The use of international RLS criteria for diagnosis and statistical methods (chi-squared tests, logistic regression) is appropriate for the study objectives. The study method is robust and suitable for exploring associations between variables.	explore associations between RLS and other complaints (IBS, CF, CMBP), adjusting for age and sex. Results are presented clearly, with prevalence rates and odds ratios reported with confidence intervals, enhancing the reliability and interpretability of findings.	patients compared to general population surveys (5-10%). Significant associations are found between RLS and IBS, CF, and CMBP, highlighting clinical relevance. The severity of RLS symptoms and low pharmacological treatment rates are also highlighted, suggesting a gap in clinical management awareness.	clinical confirmation of RLS diagnosis, potential underestimation of RLS prevalence due to diagnostic criteria variations over time, and lack of information on patients' reasons for GP consultations and mental health conditions, which could confound associations. Sampling bias towards patients consulting GPs also limits generalizability to the broader population.
Boers et al. (2022)	The research question is clearly stated: to assess the balance of benefit and harm of low-dose glucocorticoid therapy in senior patients with active rheumatoid arthritis. The question is relevant and addresses an important	The methodology appears robust with a pragmatic double-blind randomized trial design, which is appropriate for assessing real-world effectiveness. Inclusion criteria were tailored to seniors, enhancing external validity. Cotreatments were	Longitudinal models were used to analyze the data, which is suitable for longitudinal clinical trial data. One-sided testing with 95% confidence limits was employed, which is acceptable for confirmatory trials.	The results are clearly presented and include both benefit outcomes (reduced disease activity and joint damage progression) and harm outcomes (adverse events). The proportions and relative risks are reported, aiding	The study acknowledges several limitations, including the relatively short duration (2 years), which may not capture all long-term effects. Additionally, the study population was restricted to seniors with comorbidities,

	clinical need.	allowed, mimicking clinical practice. However, detailed information on randomization and blinding procedures would strengthen the methodology section.	However, further details on the statistical methods used would enhance transparency and reproducibility.	interpretation. However, more information on the specific adverse events and their severity would provide a clearer understanding of the risks associated with low-dose glucocorticoid therapy.	limiting generalizability to younger or healthier populations. The study also experienced discontinuations, some related to adverse events, which may introduce bias. Further discussion on potential biases and limitations in data collection or analysis would strengthen the discussion section.
Bolsius et al. (2021)	The research question is clearly articulated: to explore the involvement of circadian clock genes in sleep, stress, and memory, and to discuss their potential functions independent of circadian timekeeping. This	The methodology involves reviewing existing literature on the regulation of clock genes by sleep loss and stress in animal models, including acute and chronic stress paradigms. While the approach is appropriate for a review article,	Data analysis involves synthesizing findings from various studies rather than conducting primary data analysis. The quality depends on the rigor of selecting studies, analyzing their findings, and drawing conclusions based on the reviewed evidence.	The results section synthesizes findings from multiple studies on the impact of sleep loss and stress on clock gene expression in various brain regions and peripheral tissues.	The limitations of this review include potential biases in the selection of studies, heterogeneity of study designs and populations, and the inherent limitations of the studies included (such as small sample sizes, inconsistent

	question sets a comprehensive scope for the review.	specific details on the search strategy and inclusion/exclusion criteria are not provided.			methodologies across studies, etc.). These should be explicitly addressed to provide a balanced view of the existing literature.
Brady et al. (2018)	The research question is well-defined and significant, investigating the association between sleep duration, obesity, adipokines, and insulin resistance in individuals at high risk of type 2 diabetes mellitus (T2DM). The study aims to identify how variations in sleep duration correlate with metabolic and inflammatory markers, contributing to the understanding of risk factors for T2DM.	The methodology is appropriate for the research question, utilizing a large sample size (2848 participants) and comprehensive data collection, including fasting blood samples, glycaemic status, anthropometric measurements, physical activity, and self-reported sleep duration. The categorization of sleep duration into specific intervals allows for detailed analysis. However, reliance on self-reported sleep duration can introduce bias, and objective sleep measurements could provide more accurate data.	The data analysis is thorough, using regression models to explore linear and quadratic relationships between sleep duration and various metabolic and inflammatory markers. The adjustment for potential confounders, such as age, sex, ethnicity, body mass index, waist circumference, and physical activity, strengthens the validity of the findings. The analysis effectively demonstrates significant associations between sleep duration and obesity measures, as well as insulin	The results indicate that both short and long sleep durations are significantly associated with higher body mass index, body weight, and waist circumference. The study finds that 6-7 hours of sleep per 24 hours is associated with the lowest obesity measures. Additionally, fasting insulin and the leptin:adiponectin ratio are positively associated with sleep duration, while adiponectin levels are negatively associated. These findings support the hypothesis that sleep duration is linked	Limitations include the use of self-reported sleep duration, which may be subject to reporting bias and inaccuracies. The cross-sectional design of the study limits the ability to infer causality between sleep duration and metabolic outcomes. Additionally, the study does not explore the potential mechanisms underlying the observed associations, nor does it account for other factors that could influence sleep and metabolic health, such as sleep quality, stress levels, or dietary

			resistance and adipokine levels.	to obesity and insulin resistance.	habits. Further longitudinal studies are needed to confirm these findings and elucidate causative pathways.
Bravo et al. (2007)	The research question aims to investigate the association between obstructive sleep apnea (OSA) severity, excessive daytime sleepiness (EDS), and plasma TNF-α levels in children. The hypotheses are clear and relevant to understanding the pathophysiology of EDS in pediatric OSA. However, the conflicting results reported in adults regarding TNF-α levels in mediating the association with EDS highlight the need for clarification in children.	The methodology involves polysomnography to assess OSA severity, parental responses to determine EDS, and measurement of plasma TNF-α levels. Adjustments for potential confounders, such as age, gender, and obesity, strengthen the study design. However, the reliance on parental reports for EDS assessment may introduce subjectivity and potential bias.	The data analysis appears robust, with statistical comparisons made between different groups to assess the associations between OSA severity, EDS frequency, and TNF-α levels. Adjustments for confounders enhance the reliability of the results. However, the lack of significant differences in TNF-α levels between groups suggests the need for further exploration of potential mediators of the OSA-EDS association in children.	The results indicate that children with moderate-to-severe OSA are at a significantly higher risk for EDS compared to controls, but there are no significant differences in plasma TNF-α levels between groups. Additionally, high TNF-α levels do not predict the presence of EDS in children with sleep apnea. These findings suggest that while OSA severity is associated with EDS frequency in children, plasma TNF-α levels may not be a reliable predictor of EDS in this population.	One limitation of the study is the reliance on parental reports for EDS assessment, which may introduce subjectivity and potential bias. Additionally, the small sample size and cross-sectional design limit the generalizability of the findings and preclude the establishment of causality. Further research with larger sample sizes and longitudinal designs is needed to validate the study's findings and elucidate the underlying mechanisms linking OSA severity and EDS in children.

Campos-Rodriguez et al. (2021)	The research question is clear and focused on exploring the association between inflammation, antioxidant biomarkers, and depression in women with moderate-to-severe obstructive sleep apnea (OSA). Specifically, the study aims to investigate whether plasma levels of TNFα, IL-6, CRP, ICAM-1, CAT, SOD, and BDNF are associated with depression in this population.	The methodology involves a multicentre, cross-sectional study design, which is appropriate for exploring associations between biomarkers and depression in the specified population. Depression is assessed using the depression subscale of the Hospital Anxiety and Depression Questionnaire (HAD-D), and objective markers such as plasma biomarkers are measured. However, potential confounding variables should be considered, and the cross-sectional design limits causal inference.	The data analysis is comprehensive, employing logistic regression, linear regression, and multivariate regression analyses to assess associations between biomarkers, depression scores, and potential predictors of depression. The statistical methods used are appropriate for the study's objectives, and the results are presented clearly.	The results indicate a significant association between plasma IL-6 levels and the presence of depression in women with moderate-to-severe OSA. IL-6 levels are also positively correlated with depression scores. Additionally, low physical activity and higher Epworth Sleepiness Scale (ESS) scores are identified as independent predictors of depression in this population. The findings provide valuable insights into the potential role of inflammation and lifestyle factors in depression among women with OSA.	While the study provides valuable insights, several limitations should be considered. The cross-sectional design limits the ability to establish causal relationships between biomarkers and depression. Additionally, the study's generalizability may be limited to women with moderate-to-severe OSA, and potential confounding variables such as comorbidities and medication use are not fully accounted for. Further longitudinal research is needed to confirm the observed associations and explore potential underlying mechanisms.
Casal-Dominguez et al. (2019)	The research question aims to compare the efficacy and adverse	The methodology involves a retrospective comparative analysis of	The data analysis compares clinical outcomes and adverse	The results indicate that there were no significant differences	The abstract does not explicitly discuss limitations, but

effects of azathioprine (AZA) versus methotrexate (MTX) in patients with antisynthetase syndrome (ASyS). This question is relevant as it addresses treatment options for ASyS, a condition characterized by muscle weakness and systemic autoimmune features. By comparing the outcomes of patients treated with AZA versus MTX, the study provides valuable insights into the management of ASyS. Overall, the research question is clear, focused, and addresses an important clinical issue.

clinical outcomes in patients with ASyS treated with either AZA or MTX. The study compares changes in muscle strength, creatine kinase (CK) levels, corticosteroid dose, and the prevalence of adverse effects between the two treatment groups. While the retrospective design limits the ability to establish causality and control for confounding variables, it allows for the assessment of real-world treatment outcomes in a relatively large sample of ASyS patients. However, prospective randomized controlled trials would provide stronger evidence and minimize bias. Overall, the methodology is appropriate for addressing the research question but has inherent limitations

events between patients treated with AZA versus MTX. Statistical comparisons are made to evaluate differences in muscle strength recovery, CK levels, corticosteroid tapering, and the prevalence of adverse effects. The results are presented clearly, and appropriate statistical tests are used to assess significance. However, additional details on the specific statistical methods and adjustments for potential confounders would enhance the rigor of the analysis. Overall, the data analysis appears sound, but more detailed reporting would improve transparency.

in muscle strength recovery, CK decrease, corticosteroid tapering, or the prevalence of adverse events between ASyS patients treated with AZA versus MTX. This suggests that both medications have similar efficacy and safety profiles in this patient population. The occurrence of reversible pneumonitis in patients treated with MTX highlights a rare but important adverse event associated with MTX use. The findings are clearly presented and directly address the research question, providing valuable insights for clinical practice. Overall, the results are well-supported by the data and contribute to the understanding of treatment options for ASyS.

potential limitations include the retrospective study design, which may introduce bias and confounding factors. Additionally, the study relies on medical records, which may lack detailed information on certain variables or outcomes. The lack of randomization and control for confounders may limit the ability to draw causal inferences from the findings. Furthermore, the sample size may affect the statistical power to detect differences between treatment groups, especially for rare adverse events. Despite these limitations, the study provides valuable insights into the comparative effectiveness and

		inherent to its retrospective nature.			safety of AZA and MTX in ASyS, but cautious interpretation is warranted.
Chandra et al. (2019)	The research question aims to investigate sleep quality, depression, restless legs syndrome (RLS), and the role of interleukin-6 (IL-6) in patients with hematological malignancies. It addresses a gap in the literature and has clinical relevance.	Sixty-six subjects with hematological malignancies were included in the study. Sleep quality, depression, and RLS were assessed using validated tools, and serum IL-6 levels were measured at baseline and after chemotherapy initiation. The study followed patients for 6 months. The methodology is suitable for the research objectives, but more details on confounder exclusion criteria and IL-6 measurement methods would enhance clarity.	The data analysis appears robust, with appropriate statistical methods used to examine associations between variables. However, specific details about the statistical methods employed are not provided, which could affect the reproducibility of the results.	The study reports a high prevalence of poor sleep quality, depression, and RLS among subjects with hematological malignancies. Disturbed sleep at baseline was associated with depression and poor 6-month survival. However, serum IL-6 did not show associations with sleep quality, RLS, or depression. High baseline IL-6 levels and a decrease after chemotherapy were associated with poor 6-month survival. The findings provide valuable insights into the prognostic implications of sleep disturbances and IL-6	One limitation is the relatively small sample size, which may affect the generalizability of the findings. Additionally, the study's observational nature limits causal inference, and potential confounders not addressed in the analysis could influence the results. Further research with larger cohorts and longitudinal designs is needed to confirm the findings and elucidate underlying mechanisms.

				levels in hematological malignancies.	
Charoenwoodhipong et al. (2020)	The research question is clearly stated and addresses an important aspect of systemic lupus erythematosus (SLE) management. The objective aims to investigate the associations between dietary intake of omega-3 (n-3) and omega-6 (n-6) fatty acids and patient-reported outcomes in SLE, filling a significant gap in understanding the impact of nutrition on SLE symptoms.	The study utilizes a population-based cohort, enhancing the generalizability of the findings to the broader SLE population. Dietary intake and patient-reported outcomes are assessed using validated tools, strengthening the reliability of the methodology. Adequate adjustments are made for potential confounders, including age, sex, race, and body mass index.	The data analysis employs appropriate multivariable regression techniques, adjusting for potential confounders. Results are presented with 95% confidence intervals, enhancing the robustness of the findings. The analysis elucidates associations between dietary fatty acid intake and patient-reported outcomes, providing valuable insights into the relationship between nutrition and SLE symptoms.	The results are clearly presented and demonstrate significant associations between dietary intake of n-3 and n-6 fatty acids and patient-reported outcomes in SLE. Higher intake of n-3 fatty acids is associated with lower lupus activity and improved sleep quality, while a higher n-6:n-3 ratio is associated with increased lupus activity. These findings provide important implications for dietary interventions in SLE management.	Although the study provides valuable insights, limitations include its observational nature, which precludes establishing causality. Additionally, reliance on self-reported dietary intake and patient-reported outcomes may introduce recall bias. Further research is needed to validate these findings and explore potential mechanisms underlying the observed associations.
Cheng et al. (2016)	The research question is clearly stated and addresses an important gap in understanding the relationship between insomnia and nutrition in a large-	The methodology involves a well-defined cohort study with a large sample size, enhancing the generalizability of the findings. Dietary intake and insomnia	The data analysis employs appropriate statistical methods, including adjustments for potential confounders. The use of adjusted mean	The results are clearly presented and indicate significant associations between probable insomnia and higher energy intake, as well as specific dietary	While the study provides valuable insights, limitations include its observational nature, which precludes establishing causality.

	scale, population-based cohort study. The objective is relevant and contributes to existing literature on the adverse health outcomes associated with insomnia.	symptoms are assessed using validated tools, strengthening the study's methodology. Adequate adjustments are made for potential confounders.	differences and 95% confidence intervals enhances the robustness of the analysis. Trend analyses are conducted where appropriate, adding depth to the interpretation of results.	components such as trans fat, sodium, and vegetables. The findings regarding individual insomnia symptoms further elucidate the relationship between sleep disturbances and dietary habits.	Additionally, reliance on self-reported insomnia symptoms and dietary intake may introduce recall bias. Further research is needed to validate these findings in diverse populations and assess causality.
Christie et al. (2020)	Clear and significant. Quantifying prevalence and bias of study designs in environmental and social sciences is important for methodological research.	The authors use a large and comprehensive dataset of published studies (over 45,000 articles). They categorize studies based on design type (observational vs. experimental) and presence of control groups.	The authors use appropriate statistical methods to analyze the distribution of study designs and their association with factors like publication year and field of study.	The study reveals a dominance of observational designs, particularly those lacking control groups, which can limit causal inference. The authors identify potential biases related to study design and highlight areas where stronger research methods are needed.	The study doesn't assess the quality of individual studies beyond design type.
Coksevim et al. (2018)	The research question is clearly defined, aiming to investigate the effects of combination therapy with global postural reeducation exercise	The methodology involves a controlled study in which 60 patients with active AS were distributed into three groups: anti-TNF therapy plus GPR	The data analysis likely involves comparing the changes in clinical parameters before and after treatment between the three groups using appropriate statistical	The results indicate that both groups receiving exercise and anti-TNF therapy show significant improvements in clinical parameters	The study may have several limitations, including the relatively small sample size and the lack of blinding, which may introduce bias.

	(GPR) and anti-TNF treatments on clinical parameters in patients with active Ankylosing spondylitis (AS). This addresses an important aspect of AS management, evaluating the efficacy of combining exercise therapy with anti-TNF treatment, which is a common approach in clinical practice. The question is comprehensive, assessing various clinical parameters to provide a comprehensive understanding of treatment outcomes in AS patients.	program, anti-TNF therapy plus conventional exercise therapy, and a control group. Patients were assessed before and after treatment using various clinical parameters, including pain, disease activity, functionality, mobility, fatigue, sleep quality, and depression. The study design allows for the comparison of treatment outcomes between different therapeutic approaches, providing valuable insights into the effectiveness of combination therapy in AS patients.	tests, such as ANOVA or non-parametric tests. Subgroup analyses may also be conducted to compare the efficacy of different treatment modalities within each group. The significance of improvements in clinical parameters is determined based on statistical significance levels. The study may also assess correlations between changes in different clinical parameters to explore potential relationships.	compared to the control group after treatment. Furthermore, the group receiving anti-TNF therapy plus GPR exercise therapy demonstrates greater improvements in pain, walking performance, and mobility compared to the group receiving anti-TNF therapy plus conventional exercise therapy. These findings suggest that combination therapy with GPR exercise may be more effective in improving pain, function, and mobility in patients with active AS.	Additionally, the study does not assess long-term outcomes or evaluate potential adverse effects of the interventions. Furthermore, the study design does not allow for the determination of causality, as other factors may contribute to the observed improvements in clinical parameters. Addressing these limitations, such as conducting larger randomized controlled trials with longer follow-up periods, would strengthen the validity and generalizability of the findings.
Covassin et al. (2022)	The research question	The methodology is	The data analysis is	The results	Limitations include the

is well-defined and significant, aiming to investigate the effects of experimentally-induced sleep curtailment on energy intake, energy expenditure, and regional body composition, particularly focusing on body fat distribution. The study addresses a crucial aspect of how sleep deprivation might contribute to obesity and fat accumulation, which is relevant given the increasing prevalence of sleep disorders and obesity.

robust and appropriate for the research question. The study design includes a randomized, controlled, crossover, 21-day inpatient study, providing a high level of control over variables and reducing potential confounders. The use of repeated measures for energy intake, energy expenditure, body weight, body composition, fat distribution, and circulating biomarkers allows for comprehensive and accurate data collection. The controlled environment and crossover design enhance the reliability of the results, though the small sample size (12 participants) is a limitation.

thorough, using appropriate statistical methods to compare energy intake, energy expenditure, and changes in body composition between the sleep restriction and control conditions. The results clearly indicate significant differences in caloric intake and fat accumulation during sleep restriction. However, the study does not find significant changes in total body fat, focusing instead on regional fat distribution, which is a key strength in understanding the specific effects of sleep loss on abdominal fat accumulation. The analysis effectively highlights the impact of sleep restriction on increased caloric intake and central fat accumulation.

demonstrate that sleep restriction leads to increased caloric intake, particularly from protein and fat, without changes in energy expenditure. This imbalance results in weight gain and specifically in the accumulation of abdominal fat, including both subcutaneous and visceral fat. These findings provide strong evidence that sleep loss predisposes individuals to central obesity, a key risk factor for metabolic diseases. The study successfully establishes a link between sleep restriction and adverse changes in body fat distribution, which has significant implications for understanding the relationship between sleep and obesity.

small sample size and short duration, which may limit the generalizability and long-term applicability of the findings. The study's controlled setting might not fully replicate real-world conditions, and the participants being healthy and nonobese limits the applicability of the results to other populations, such as those who are already obese or have metabolic disorders. Additionally, the study does not explore potential underlying mechanisms, such as hormonal changes or appetite regulation, that might explain the observed increase in caloric intake and fat accumulation during sleep restriction. Further research with larger, more diverse populations and

					longer follow-up periods is needed to confirm these findings and explore underlying mechanisms.
Crofford et al. (1997)	The research question is well-defined, focusing on the circadian secretory dynamics of ACTH, cortisol, and IL-6 in early untreated RA patients. The study aims to understand the interactions between these hormones and cytokines, which is crucial for elucidating the pathophysiology of systemic symptoms in RA and the role of the HPA axis in mediating inflammation.	The methodology is robust and appropriate for the research question. The use of serial blood sampling over 24 hours, followed by oCRH administration, allows for detailed analysis of circadian hormone dynamics and their interactions. The inclusion of a well-matched control group strengthens the comparative analysis. However, the small sample size (five RA patients and five controls) is a limitation that may affect the generalizability of the findings.	The data analysis is comprehensive, examining 24-hour levels, circadian variations, and time-lagged cross-correlations of ACTH, cortisol, and IL-6. The statistical analyses are appropriate and provide insights into the temporal relationships between these variables. The study identifies significant differences in IL-6 levels and their temporal correlations with ACTH and cortisol, adding depth to the understanding of HPA axis function in early RA.	The results indicate that while overall HPA axis activity remains normal, there are specific circadian variations and temporal correlations between IL-6 and ACTH/cortisol in early untreated RA patients. The earlier morning surge of ACTH and cortisol and the positive temporal correlation with IL-6 suggest a complex regulatory mechanism. The data also suggest that endogenous IL-6 stimulates ACTH and cortisol secretion, but the HPA axis response may be insufficient to counteract ongoing inflammation.	Limitations include the small sample size, which limits the power and generalizability of the study. Additionally, the study focuses on early untreated RA patients, which may not reflect the HPA axis dynamics in patients with longer disease duration or those undergoing treatment. The short duration of the study (24 hours) may not capture long-term variations and effects. Potential confounding factors, such as variations in disease severity and individual stress responses, are not addressed.

Crowson et al. (2022)	The study effectively addresses the research question by confirming a list of 44 morbidities in patients with RA and assessing additional morbidities and lookback periods.	The methodology is robust, employing a retrospective cohort design with rigorous criteria for case definition (ACR classification criteria) and extensive medical record review.	Data analysis is comprehensive, utilizing descriptive statistics, logistic regression models adjusted for age and sex, and false discovery rate adjustments for multiple comparisons.	Results are clearly presented, showing significantly higher prevalence of multimorbidity in RA compared to non-RA cohorts across multiple morbidities.	The study acknowledges limitations including potential biases from retrospective data, variability in coding practices, and the challenge of defining chronic conditions uniformly.
Cui et al. (2018)	The research question is clearly defined: to evaluate the association of Primary Sjögren's syndrome (pSS) with depression. The question addresses an important gap in understanding the prevalence of depression in pSS patients and has significant clinical implications for early recognition and appropriate intervention.	The methodology involves a systematic review and meta-analysis of published literature up to October 2016, utilizing multiple databases to identify relevant studies. Studies were screened according to inclusion and exclusion criteria, and the quality of included studies was evaluated. Data analysis was conducted using Revman5.2 software, and psychometric measures such as PHQ-9, HADS, CES-D, Zung depression scale, and BDI were used to assess depression	The data analysis appears appropriate for the study objectives, utilizing meta-analysis techniques to estimate the summary odds ratio (OR) and standardized mean difference (SMD) of depression prevalence and scores in pSS patients compared to controls. Statistical significance levels and confidence intervals are reported, aiding in the interpretation of results. The use of Revman5.2 software enhances the standardization of data	The results are presented clearly, demonstrating a significant association between pSS and increased prevalence of depression, as well as higher depression scores in pSS patients compared to controls. The summary odds ratio and standardized mean difference values provide quantitative estimates of the magnitude of associations. The inclusion of statistical significance levels and confidence intervals enhances the reliability	The study acknowledges several limitations, including the potential for publication bias and the exclusion of studies published after October 2016. The quality assessment of included studies may introduce bias, and the use of multiple psychometric measures for depression assessment may limit comparability across studies. Additionally, the meta-analysis relies on aggregated data rather than

		severity. The study design allows for a comprehensive evaluation of the association between pSS and depression, incorporating a large sample size and standardized assessment tools. However, the search was limited to databases established until October 2016, potentially missing more recent studies, and the quality assessment of included studies may introduce bias. Further details on the search strategy and quality assessment criteria would enhance transparency.	analysis procedures. However, more information on potential sources of heterogeneity and sensitivity analyses would provide a deeper understanding of the findings.	of the findings. Additionally, the clinical implications for early recognition and intervention are emphasized. However, further exploration of potential moderators and subgroup analyses would provide additional insights.	individual patient data, which may obscure important nuances. Further discussion on these limitations and their implications for interpretation would strengthen the discussion section.
Curtis et al. (2006)	The research question aims to determine the prevalence of glucocorticoid-associated adverse events (AEs) in a large US managed care	The methodology involves identifying adults receiving >or=60 days of glucocorticoids using linked administrative and pharmacy claims data.	The data analysis involves examining the prevalence of 8 self-reported glucocorticoid-associated AEs and assessing their	The results indicate that over 90% of individuals receiving glucocorticoids reported at least one adverse event (AE), with 55% reporting that	The abstract does not explicitly discuss the limitations of the study. However, potential limitations can be inferred, including the reliance

population, focusing on individuals receiving >or=60 days of glucocorticoids. Specifically, the study investigates the frequency of 8 commonly attributed AEs associated with glucocorticoid use and explores the dose-dependent association between cumulative glucocorticoid use and the prevalence of these AEs. The research question is clearly defined, relevant, and addresses a gap in understanding the prevalence and dose-dependent nature of glucocorticoid-related AEs in real-world clinical practice. By providing insights into the frequency and severity of AEs, the study contributes to optimizing patient care and risk management strategies in These individuals were then surveyed about glucocorticoid use and symptoms of 8 AEs commonly attributed to glucocorticoid use. The study utilized a large US managed care population, enhancing the generalizability of the findings. The survey response rate was reasonable (38%), and efforts were made to adjust for potential confounders through multivariable analysis. However, the reliance on self-reported symptoms may introduce bias or underreporting, and the use of administrative data may limit the accuracy and completeness of clinical information. Despite these limitations, the methodology is appropriate for addressing the research question and provides valuable insights into the association with cumulative and average glucocorticoid dose in a dose-dependent manner. Multivariable adjustment was performed to account for potential confounders. The analysis demonstrates a strong dose-dependent association between cumulative glucocorticoid use and the prevalence of AEs, with increasing duration of use significantly associated with specific AEs, even among users of low-dose therapy (<or=7.5 mg of prednisone per day). The results provide valuable insights into the relationship between glucocorticoid dosage, duration, and the prevalence of AEs, supporting the study's conclusions and at least one AE was very bothersome. Weight gain was the most commonly reported AE, followed by cataracts and fractures. Importantly, all AEs demonstrated a strong dose-dependent association with cumulative glucocorticoid use, highlighting the significance of glucocorticoid dosage in AE prevalence. Among users of low-dose therapy, increasing duration of use was significantly associated with specific AEs, emphasizing the importance of long-term monitoring and risk management in glucocorticoid therapy. The results effectively address the research question and underscore the need for physicians to be vigilant for on self-reported symptoms, which may introduce bias or underreporting, and the use of administrative data, which may limit the accuracy and completeness of clinical information. Additionally, the study's retrospective design may preclude the establishment of causal relationships, and the survey response rate of 38% may raise concerns about response bias. Despite these limitations, the study provides valuable real-world data on the prevalence and dose-dependent association of glucocorticoid-associated AEs, informing clinical practice and risk management strategies. Addressing these limitations in

	glucocorticoid therapy. Overall, the research question is appropriate and well-aligned with the study objectives.	prevalence and dose-dependent association of glucocorticoid-associated AEs in real-world clinical practice.	implications for clinical practice. Overall, the data analysis is robust and effectively addresses the research question.	glucocorticoid-related AEs and counsel patients about potential risks, even at low doses. Overall, the results provide valuable insights into the prevalence and dose-dependent nature of glucocorticoid-associated AEs in real-world clinical practice.	future research, such as employing prospective study designs and comprehensive clinical assessments, would enhance the robustness and generalizability of the findings.
Cutolo et al. (1999)	The research question is relevant and timely, aiming to explore the influence of melatonin (MLT) on the production of IL-12 and nitric oxide (NO) in RA synovial macrophages. This is significant for understanding the diurnal rhythmicity of melatonin and its impact on immune responses and inflammation in RA, particularly in relation to symptoms like joint	The methodology involves primary cultures of RA synovial macrophages and human myeloid monocytic cells (THP-1), with treatments including MLT and lipopolysaccharide (LPS) stimulation. The experimental design is robust, allowing for comparison between treated and control groups, and the use of both primary cells and cell lines enhances the reliability of the findings.	Data analysis is rigorous, employing statistical tests to compare IL-12 and NO production across different treatment groups. The significant findings are clearly presented, demonstrating the effects of MLT on cytokine and NO production. The comparison between MLT-stimulated and LPS-prestimulated macrophages provides	The results indicate that MLT induces IL-12 and NO production in unstimulated RA synovial macrophages and THP-1 cells. Notably, MLT decreases IL-12 production in LPS-prestimulated macrophages, suggesting a modulatory effect dependent on prior cell activation. These findings support the role of MLT in immune	Limitations include the in vitro nature of the study, which may not fully replicate the in vivo environment of RA patients. The study also does not explore the long-term effects of MLT treatment or its impact on clinical outcomes in RA. Additionally, the unexpected results regarding NO production in LPS-treated macrophages warrant further

	morning stiffness.	However, further in vivo studies would be necessary to validate these results.	insights into the dose-dependent and priming effects of MLT.	response modulation and its potential impact on RA symptoms.	investigation to understand the underlying mechanisms. Further research is needed to confirm these findings and elucidate the precise role of MLT in RA pathophysiology.
Dardin et al. (2021)	The research question addresses a significant gap in knowledge by investigating the feasibility, effectiveness, and safety of a resistance exercise program for reducing fatigue in patients with primary Sjogren's syndrome (pSS). It aims to evaluate the potential benefits of resistance training in improving various aspects of health and well-being in this patient population. The question is clear, relevant, and contributes to the	The study utilized a parallel, single-blind randomized trial design, which is appropriate for assessing the effectiveness of an intervention compared to a control group. Participants were appropriately recruited based on inclusion criteria, and randomization was employed to allocate them into the resistance training group (RT) or control group (CG). The resistance exercise program was well-described, including details on exercise intensity, duration, and	Data analysis involved comparing outcomes between the RT and CG groups using one-factor repeated-measures analysis of variance (ANOVA) to assess intergroup and intragroup variability. The statistical approach appears suitable for evaluating the effectiveness of the resistance exercise program in improving fatigue, pain, functional capacity, emotional aspects, vitality, and subjective perception of disease activity. However, additional details on specific	The results indicate that the resistance training program effectively improved fatigue, pain, functional capacity, emotional aspects, vitality, and subjective perception of disease activity in women with pSS. However, no significant differences were found between the RT and CG groups in certain outcomes after the training period. The findings are presented clearly and align with the study objectives, demonstrating the potential benefits of	While the study provides valuable insights, several limitations should be considered. These include the relatively small sample size and potential selection bias due to the single-center design. Additionally, the study's duration of 16 weeks may not capture long-term effects of resistance training on fatigue and other outcomes in pSS patients. Future research with larger sample sizes, longer follow-up periods, and multicenter designs

	understanding of non-pharmacological interventions for managing fatigue in pSS.	frequency. The methodology appears robust for evaluating the impact of resistance training on fatigue and other outcomes in pSS patients.	statistical tests and adjustments for potential confounders would enhance the transparency of the analysis.	resistance training for managing fatigue and improving various aspects of health in pSS patients.	would help validate and generalize the findings.
Davies et al. (2012)	The research question is clearly articulated: to assess the utility of a low glycemic index (GI) diet in achieving weight loss and improving glycemic control in patients with corticosteroid-dependent systemic lupus erythematosus (SLE). This question addresses an important clinical issue related to weight gain and cardiovascular risk in patients with SLE who require prolonged corticosteroid therapy. The study aims to investigate whether dietary intervention	The study employed a randomized controlled trial design involving 23 women with stable SLE who were receiving corticosteroids and had a body mass index (BMI) > 25 kg/m(2). Subjects were randomly assigned to either a low GI diet or a calorie-restricted (low cal) diet for a duration of 6 weeks. Various outcome measures including weight loss, waist and hip measurements, biomarkers of cardiovascular risk, disease activity, fatigue, and sleep quality were assessed. The	The data analysis involved comparing the outcomes between the two diet groups using appropriate statistical tests such as paired t-tests. Both low GI and low cal diets led to significant weight loss, improvements in waist and hip measurements, and reductions in fatigue severity scale scores. The results were presented clearly, and the statistical significance of the findings was appropriately reported. The analysis appears to have been conducted	The results indicate that both the low GI and low cal diets led to significant weight loss and improvements in waist and hip measurements in patients with corticosteroid-dependent SLE. There were also significant reductions in fatigue severity scale scores in both diet groups. However, there were no statistically significant differences in weight loss and other outcomes between the two diet groups. Overall, the findings suggest that	While the study provides valuable insights into the potential benefits of dietary interventions in patients with corticosteroid-dependent SLE, several limitations should be considered. These include the small sample size, short duration of the intervention, and lack of long-term follow-up to assess sustainability of the effects. Additionally, the study only included women, limiting generalizability to male patients with

	can mitigate these adverse effects in this population.	methodology appears appropriate for evaluating the efficacy and safety of the dietary interventions in achieving weight loss and improving other relevant outcomes in patients with corticosteroid-dependent SLE.	rigorously and effectively.	both dietary interventions are effective and well-tolerated in this patient population, with potential benefits for weight management and fatigue reduction.	SLE. Further research with larger sample sizes, longer follow-up periods, and inclusion of diverse patient populations is needed to confirm these findings and elucidate the clinical implications of dietary interventions in this context.
Detert et al. (2016)	The research question aims to compare the effects of etanercept (ETA) and methotrexate (MTX) on sleep quality, disease activity, and patient-reported outcomes in patients with rheumatoid arthritis. The objectives are clearly defined and relevant to addressing the comparative	The methodology involves an open, prospective study design with a 16-week duration, including 36 patients with rheumatoid arthritis (RA) with a 28-joint Disease Activity Score (DAS28CRP) ≥3.2. Patients were assigned to receive either methotrexate (MTX) or etanercept (ETA), alone or in combination with	The data analysis involves comparing baseline characteristics and outcomes between the ETA and MTX groups, focusing on disease activity (DAS28CRP), patient-reported outcomes (SF-36, HAQ-DI), and sleep parameters obtained from polysomnography. Statistical significance	The results section presents key findings related to the comparative efficacy of etanercept (ETA) and methotrexate (MTX) in patients with rheumatoid arthritis (RA). Baseline characteristics did not differ significantly between the two treatment groups, except for disease	The study acknowledges several limitations that warrant consideration. These limitations include the small sample size, potential selection bias inherent in the open, prospective study design, and the lack of randomization. Additionally, the study duration of 16

efficacy of these two treatments in improving various parameters related to rheumatoid arthritis management. By focusing on sleep quality, disease activity, fatigue, and immune parameters, the research question encompasses key aspects of patient well-being and treatment response. Overall, the research question is well-articulated and aligns with the study's objectives and methodology.

MTX, and various clinical, laboratory, sleep, functional, immunological, and neuroendocrine parameters were assessed at baseline, week 8, and week 16. The study design allows for direct comparison of the two treatment modalities and includes comprehensive assessments of relevant outcomes. However, the study could benefit from a larger sample size and a randomized controlled trial design to minimize bias and improve the robustness of the findings. Additionally, the inclusion of polysomnography for sleep assessment adds depth to the methodology by providing objective measures of sleep quality. Overall, while the methodology is sound, there is room for

was assessed using appropriate tests, and changes in outcomes over time were analyzed within each treatment group. The analysis demonstrates significant improvements in disease activity, SF-36 scores, and HAQ-DI scores in both treatment groups, with no statistically significant differences between the ETA and MTX groups in terms of DAS28CRP improvements at week 16. Furthermore, the ETA group showed significantly improved sleep parameters compared to the MTX group. The data analysis is clear, concise, and effectively communicates the main findings of the study, providing insights into the comparative efficacy of ETA and

duration. Both ETA and MTX resulted in significant improvements in disease activity (DAS28CRP), patient-reported outcomes (SF-36, HAQ-DI), and C-reactive protein (CRP) levels. However, improvements in DAS28CRP were more pronounced in the ETA group, although not statistically significant compared to the MTX group. Importantly, the ETA group demonstrated significantly improved sleep parameters, including sleep efficiency, total sleep time, and stage 2 sleep duration, compared to the MTX group. Overall, the results highlight the effectiveness of both therapies in improving disease activity and patient-reported

weeks may not capture long-term treatment effects and outcomes. Furthermore, the study's reliance on self-reported measures, such as patient-reported outcomes and visual analogue scale assessments, may introduce subjectivity and recall bias. The study could also benefit from more detailed reporting of adverse events and tolerability of treatments. Despite these limitations, the study provides valuable insights into the comparative efficacy of ETA and MTX in patients with RA, particularly in terms of sleep quality, disease activity, and patient-reported outcomes. Future research with larger

		improvement in terms of study design and sample size.	MTX in improving various outcomes in patients with RA.	outcomes, with ETA showing additional benefits in sleep quality.	sample sizes and longer follow-up periods could address some of these limitations and further elucidate the comparative effectiveness of these treatments in RA management.
du Montcel et al. (2005)	The research question is clearly defined and addresses the need for re-evaluation of the shared epitope hypothesis in rheumatoid arthritis.	The methodology involves the recruitment of RA patients from a reputable consortium and utilizes a novel classification of HLA-DRB1 alleles.	The data analysis appears robust, employing statistical tests to assess the fit of the new HLA-DRB1 classification in two distinct patient groups.	The results are presented clearly, demonstrating how the new classification explains the observed data regarding HLA-DRB1 genotypes in RA patients.	While the study offers valuable insights, limitations may include potential biases in patient selection and the need for replication in diverse populations.
El-Sharkawy et al. (2019)	The study aims to investigate the additive effect of melatonin supplementation in individuals with generalized chronic periodontitis (gCP) and primary insomnia after	The research employs a randomized clinical trial (RCT) design, which is an appropriate method to evaluate the efficacy of interventions. The sample size of 74 gCP patients with primary	Data analysis involves comparing key outcomes, such as CAL gain, PD reduction, bleeding on probing (BOP %), salivary TNF-α levels, and Athens insomnia scale (AIS)	The study findings indicate significantly greater CAL gain and PD reduction in the melatonin group compared to the control group at both 3 and 6 months post-	While the study provides valuable insights, several limitations should be acknowledged. Firstly, the study's generalizability may be limited due to the

scaling and root planing (SRP), focusing on its potential benefits for periodontal health, particularly in terms of clinical attachment level (CAL) gain, pocket depth reduction (PD), and salivary TNF-α levels.	insomnia is adequate for the study's objectives. Random allocation into melatonin and control groups helps minimize selection bias. The use of melatonin capsules and matching placebos ensures blinding, enhancing the study's reliability. The measurement of various clinical parameters and salivary TNF-α levels as primary and secondary endpoints is comprehensive, providing a holistic assessment of periodontal health. However, the study could benefit from additional details on the randomization process, allocation concealment, and blinding procedures to strengthen the methodological rigor.	scores between the melatonin and placebo groups at different time points (3 and 6 months post-therapy). Statistical significance is appropriately determined using tests such as t-tests, with a significance level set at $P < 0.01$ to reduce the likelihood of Type I errors. The statistical approach appears robust and aligns with the study's objectives. However, the absence of details on adjustments for potential confounders or subgroup analyses limits the depth of the data analysis.	therapy, highlighting the potential benefits of melatonin supplementation in enhancing periodontal health outcomes. Additionally, lower salivary TNF-α levels and AIS scores in the melatonin group suggest a potential role in reducing inflammation and insomnia severity. The improvement in BOP% in both groups underscores the efficacy of SRP in periodontal therapy, regardless of melatonin supplementation. However, the lack of correlation between salivary TNF-α levels and other clinical variables warrants further investigation. Overall, the results support the hypothesis that melatonin supplementation adjunctive to SRP may	inclusion of only gCP patients with primary insomnia, potentially restricting the applicability of findings to broader patient populations. Secondly, the absence of detailed information on patient characteristics, such as comorbidities or medication use, limits the ability to assess potential confounding factors. Additionally, the short-term nature of the study (6 months) may not capture long-term effects of melatonin supplementation on periodontal health. Furthermore, the lack of mechanistic insights into the observed improvements and the absence of long-term follow-up data are notable limitations. Future studies addressing these

				offer clinical benefits for gCP patients with primary insomnia.	limitations would provide a more comprehensive understanding of the role of melatonin in periodontal therapy.
Enns et al. (2018)	The research question is well-defined: to evaluate the association between pain, fatigue, depression, anxiety, and work impairment in patients with immune-mediated inflammatory diseases (IMID), specifically multiple sclerosis, inflammatory bowel disease, and rheumatoid arthritis, as well as in a depression and anxiety group. The question addresses an important gap in understanding the determinants of work impairment in chronic health conditions and has significant clinical implications.	The methodology involves a cross-sectional study evaluating the association between pain, fatigue, depression, anxiety, and work impairment in four patient populations. Participants completed measures assessing pain, fatigue, depression, anxiety, and work impairment, with analyses conducted using quantile regression to control for sociodemographic factors, physical disability, and cognitive deficits. The study design allows for the examination of the unique contributions of pain, fatigue, depression, and anxiety to work	The data analysis appears appropriate for the study objectives, utilizing quantile regression to assess the associations between pain, fatigue, depression, anxiety, and work impairment while controlling for sociodemographic and clinical factors. Standardized estimates are reported, aiding in the interpretation of results. However, more information on effect sizes and potential interactions between variables would provide a deeper understanding of the findings.	The results are presented clearly, demonstrating significant associations between pain, fatigue, depression, anxiety, and work impairment in patients with IMID and depression and anxiety disorders. Each distress variable individually showed significant associations with work absenteeism, presenteeism, and general activity impairment. Fatigue emerged as a significant predictor of work and activity impairment in all models when distress variables were entered concurrently. The inclusion of	The study acknowledges several limitations, including its cross-sectional design, which limits causal inference. The reliance on self-report measures of pain, fatigue, depression, anxiety, and work impairment may introduce bias, and potential confounders such as medication usage or comorbidities are not fully addressed. Additionally, the study's focus on specific chronic health conditions and a depression and anxiety group may limit the generalizability of

		impairment across different chronic health conditions. However, the cross-sectional nature of the study limits causal inference, and potential confounders such as medication usage or comorbidities are not fully addressed. Further details on participant recruitment and potential sources of bias would enhance transparency.		standardized estimates enhances the understanding of the magnitude of associations. However, further exploration of potential mechanisms underlying these associations would provide additional insights.	findings. Further discussion on these limitations and their implications for interpretation would strengthen the discussion section.
Esalatmanesh et al. (2021)	The research question addresses the efficacy of melatonin supplementation in improving disease activity, oxidative stress, inflammatory markers, and metabolic parameters in RA patients, contributing to the exploration of adjunctive therapies in RA management.	The study employs a randomized double-blind, placebo-controlled trial design with a sample size of 64 RA patients. Participants are randomly assigned to receive either melatonin supplementation (6 mg/day) or placebo for 12 weeks. Various parameters including disease activity, oxidative stress markers, lipid profile, and glucose	Data analysis involves comparing baseline and post-treatment measures within each group and between the melatonin and placebo groups. Statistical significance is determined using appropriate tests, with adjustments made for baseline measures to mitigate potential confounding factors.	Melatonin supplementation leads to significant reductions in disease activity (DAS-28) and oxidative stress (MDA levels) and improvements in lipid profile (increase in HDL-C), while showing no significant impact on inflammatory markers (ESR), total antioxidant capacity (TAC), or	Some limitations include the relatively small sample size, short duration of the intervention (12 weeks), and lack of long-term follow-up to assess sustained effects. Additionally, the study does not evaluate the impact of melatonin supplementation on other relevant outcomes such as joint

		metabolism are assessed before and after the trial.		glucose metabolism (FBS and insulin levels). The findings suggest a potential role for melatonin as an adjunctive therapy in RA.	function, quality of life, or adverse events. Further research with larger sample sizes and longer durations is warranted to validate these findings and address these limitations.
Esen et al. (2021)	The research question aims to investigate the impact of smoking on obstructive sleep apnea, addressing an important gap in the literature. However, the objectives could be more specific regarding the aspects of OSA affected by smoking, which may enhance the clarity of the study's focus.	The methodology provides a clear description of the study population, inclusion criteria, and data collection methods, including polysomnographic examination. However, the study could benefit from a more detailed explanation of how smoking status was determined, including criteria for defining smokers and assessing smoking frequency.	The data analysis compares non-smokers and smokers in terms of OSA severity, demographic factors, and polysomnographic data. While the analysis provides valuable insights into the association between smoking and OSA severity, the interpretation of results could be strengthened by considering potential confounding variables and conducting multivariate analyses.	The results highlight differences in OSA severity between male smokers and non-smokers, particularly among those with a BMI < 30. However, the lack of a statistically significant correlation between smoking frequency and OSA severity suggests a nuanced relationship that warrants further investigation. The conclusions drawn from the results are cautious and acknowledge the limitations of the study.	The study acknowledges limitations such as the relatively small sample size and the lack of statistically significant differences in PSG data between non-smokers and smokers. Additionally, the cross-sectional design limits the ability to establish causality or assess temporal relationships between smoking and OSA severity. While the limitations are acknowledged, further discussion on potential confounders and biases would enhance

					the transparency of the study.
Falup-Pecurariu et al. (2021)	The research question is clear and addresses the prevalence, clinical phenotype, and impact of Restless Legs Syndrome (RLS) on sleep in patients with Systemic Lupus Erythematosus (SLE). The study aims to fill a gap in the literature, as few studies have explored the relationship between RLS and sleep disturbances in SLE patients. This question is relevant due to the known impact of both conditions on patients' quality of life.	The study uses a prospective design, enrolling 26 SLE patients and 26 age- and sex-matched controls. It follows ethical guidelines and obtained necessary ethical approvals and informed consents. The use of validated diagnostic criteria for RLS (IRLSSG criteria) and various scales for sleep assessment (IRLS, PSQI, Epworth Sleepiness Scale) is appropriate. However, the small sample size and single-center design limit generalizability. The exclusion criteria are well-defined to minimize confounding factors.	Data analysis is conducted using appropriate statistical methods, including descriptive statistics, one-way ANOVA, and Tukey's post hoc test. The results are clearly presented with statistical significance noted ($P<0.05$ for key comparisons). However, the analysis is somewhat limited by the small sample size, which may affect the power to detect significant differences.	The study found a higher prevalence of RLS in SLE patients (34.62%) compared to controls (7.69%). RLS symptoms were more frequent and intense in the SLE group, with significant associations noted between RLS and poorer sleep quality, earlier wake-up times, and shorter sleep duration. These findings are consistent with previous studies, although the small sample size warrants caution in interpreting the results.	Internal Validity: The small sample size and the cross-sectional design limit the ability to establish causation. The study relies on self-reported data, which can introduce reporting bias. External Validity: The study is conducted at a single hospital in Romania, which may not be representative of the broader SLE population. The specific demographics and regional characteristics of the sample may limit the generalizability of the findings. Methodological Considerations: The study lacks objective sleep assessments (e.g., polysomnography) and did not perform

	electromyography or nerve conduction studies, which could have provided more detailed information on the presence of polyneuropathy. The reliance on self-reported questionnaires for RLS and sleep characteristics may introduce subjective bias. Additionally, the exclusion of certain comorbidities may limit the understanding of RLS in the broader SLE population. The study did not control for all potential confounding factors, such as medication use or lifestyle factors.
Fitzcharles et al. (2016)	The study acknowledges several limitations, including the limited number of identified RCTs, the

The research question is clearly stated and relevant, aiming to assess the potential effects of cannabinoid molecules in managing chronic pain associated with rheumatic diseases. This addresses an important gap in current treatment options, particularly in light of the global interest in the medicinal use of herbal cannabis. The question is comprehensive, covering multiple rheumatic diseases and various cannabinoid preparations, providing valuable insights for clinicians and researchers in this field.

The methodology involves a systematic search of multiple databases for randomized controlled trials (RCTs) evaluating the effects of herbal cannabis or pharmaceutical cannabinoid products on chronic pain in rheumatic diseases. The inclusion criteria are clearly specified, including study duration, sample size, and outcome measures. The quality of RCTs is assessed using the Cochrane Risk of Bias Tool. While the methodology is robust overall, the potential for publication bias and the limited number of identified RCTs may affect the

The data analysis includes the synthesis of findings from four identified RCTs involving cannabinoid treatments in fibromyalgia syndrome (FMS), chronic spinal pain, and rheumatoid arthritis (RA) pain. Outcome measures include pain reduction, sleep problems, fatigue, quality of life, tolerability, and safety. While the findings suggest inconsistent efficacy of cannabinoids compared to controls, cannabinoids were generally well-tolerated and safe during the study duration.

The results indicate insufficient evidence to recommend any cannabinoid preparations for symptom management in patients with chronic pain associated with rheumatic diseases. Despite some RCTs showing potential benefits of cannabinoids, the findings were not consistent across studies, and the risk of bias was high in some cases. The conclusions are cautious, highlighting the need for further research to establish the efficacy and safety of cannabinoids in this patient population.

high risk of bias in some studies, and the absence of RCTs in osteoarthritis (OA) patients. Additionally, publication bias and the potential heterogeneity of cannabinoid preparations and dosages may affect the interpretation of the findings. The limitations are appropriately discussed, providing context for the cautious conclusions drawn from the systematic review.

		generalizability of the findings.			
Formica et al. (2003)	The research question is clearly defined, aiming to investigate the associations of smoking and alcohol consumption with incident systemic lupus erythematosus (SLE) in African-American women. This addresses a gap in prospective research on SLE risk factors in this population.	The study utilizes a prospective cohort design, providing stronger evidence than case-control studies and minimizing recall bias. A large sample size (64,500 participants) enhances the statistical power and generalizability of the findings. Cox proportional hazards regression is an appropriate statistical method for analyzing time-to-event data.	Data analysis employs Cox proportional hazards regression to estimate incidence rate ratios with 95% confidence intervals. Adjustments are made for potential confounders in multivariate analyses, enhancing the validity of the results.	The results are clearly presented, demonstrating a suggestive increased risk of incident SLE among smokers, particularly those who began smoking before age 19 years. However, the associations did not reach statistical significance, likely due to the relatively small number of incident cases. No significant association was found between alcohol consumption and SLE risk.	While the study provides valuable insights into the associations between smoking, alcohol consumption, and incident systemic lupus erythematosus (SLE) in African-American women, its reliance on self-reported data may introduce recall bias and misclassification of exposure. Additionally, the relatively small number of incident SLE cases may limit the statistical power to

					detect associations, particularly for subgroup analyses such as age of smoking initiation.
Freitas et al. (2018)	The research question is well-defined and significant, focusing on the effect of weight loss induced by bariatric surgery on pro-inflammatory cytokine (TNF-α), anti-inflammatory adipokine (adiponectin), and an adipose-derived hormone (leptin) in severely obese subjects. The study aims to understand how surgical weight loss impacts the inflammatory state associated with obesity, which is relevant for improving patient outcomes.	The methodology is robust and appropriate for the research question, involving a randomized controlled trial with a clear inclusion and exclusion criteria. The use of bariatric surgery as an intervention and the measurement of TNF-α, adiponectin, and leptin levels through enzyme-linked immunosorbent assay (ELISA) provides reliable data. The control group enhances the validity of the findings by providing a comparative baseline. However, the relatively small sample size (55 patients) and the focus on a 6-month follow-up period are limitations that could affect the generalizability	The data analysis is thorough, comparing baseline and 6-month follow-up variables within both the control group and the bariatric surgery group. The statistical methods used to determine significance are appropriate, and the results are clearly presented, showing significant differences in the BSG group post-surgery. The study effectively highlights the impact of surgical weight loss on inflammatory markers and adipokines, providing valuable insights into the physiological changes associated with obesity and weight loss.	The results indicate that bariatric surgery leads to significant reductions in pro-inflammatory cytokine (TNF-α) and leptin levels, as well as an increase in anti-inflammatory adipokine (adiponectin) levels in the BSG group compared to the CG. These findings suggest that surgical weight loss can effectively reduce the inflammatory state (inflammome) associated with severe obesity. The lack of significant changes in the control group reinforces the impact of the surgical intervention. This	Limitations include the small sample size, which may limit the statistical power and generalizability of the findings. The study's short follow-up period (6 months) does not capture the long-term effects of bariatric surgery on inflammatory markers and adipokines. Additionally, the study does not explore other potential factors that could influence inflammation and adipokine levels, such as changes in diet, physical activity, or medication use post-surgery. Further research with larger sample sizes and longer follow-up

		and long-term applicability of the results.		supports the hypothesis that weight loss through bariatric surgery can mitigate the inflammatory state in obese patients.	periods is needed to confirm these findings and understand the long-term impacts of bariatric surgery on inflammation in obese patients.
Galbo et al. (2016)	The research question aims to investigate the diurnal variation of clinical symptoms and the concentrations of melatonin, inflammatory cytokines, and cortisol in polymyalgia rheumatica (PMR) patients. Additionally, the study explores the effects of prednisolone treatment on these parameters. The question is well-defined, relevant, and addresses an important gap in understanding the pathophysiology of PMR and the potential role of circadian rhythms in disease manifestation. By	The methodology involves studying ten glucocorticoid-naive patients newly diagnosed with PMR and seven non-PMR control subjects over a 24-hour period before treatment and on the 14th day of treatment with 20 mg/day of prednisolone. Global pain and generalized muscle stiffness were assessed using visual analogue scales, and blood samples were collected repeatedly to measure concentrations of melatonin, inflammatory cytokines, and cortisol. The study design allows for the evaluation of diurnal	The data analysis involves assessing the diurnal variation of clinical symptoms, melatonin, inflammatory cytokines, and cortisol concentrations in PMR patients and control subjects. Statistical comparisons are made between untreated PMR patients and control subjects, as well as before and after prednisolone treatment in PMR patients. The analysis includes appropriate statistical tests (e.g., t-tests, ANOVA) to determine significance levels and identify differences between groups and	The results demonstrate diurnal variations in pain, stiffness, melatonin, inflammatory cytokines (IL-6, IL-8, TNF-α, IL-1β, IL-4, IL-10), and cortisol concentrations in untreated PMR patients. These parameters peak in the early morning and decline throughout the day. Additionally, prednisolone treatment abolishes symptoms, normalizes C-reactive protein, and reduces melatonin and cytokine concentrations in PMR patients. IL-10 levels increase following prednisolone treatment. The findings	The abstract acknowledges several limitations, including the small sample size, lack of long-term follow-up, and absence of a placebo group for comparison. Additionally, the study did not investigate potential confounding factors or comorbidities that may influence symptom severity and biomarker levels. The short duration of prednisolone treatment limits the assessment of long-term effects. Furthermore, the study did not explore the mechanisms

assessing diurnal variations in symptoms and biomarkers, the study sheds light on the underlying mechanisms and supports the use of chronotherapy in PMR management. Overall, the research question is appropriate and aligns with the study objectives.

variations in symptoms and biomarkers and the effects of prednisolone treatment on these parameters. However, the small sample size and lack of long-term follow-up are notable limitations of the methodology. Additionally, the study did not include a placebo group for comparison, which could have provided further insights into the effects of prednisolone treatment. Despite these limitations, the methodology is appropriate for addressing the research question and provides valuable insights into the diurnal patterns of symptoms and biomarkers in PMR.

time points. The findings suggest diurnal variations in symptoms and biomarkers in PMR patients, with prednisolone treatment abolishing symptoms and modulating cytokine levels. Overall, the data analysis is robust and supports the study's conclusions.

suggest that melatonin may stimulate cytokine production, contributing to PMR symptoms, while cortisol may downregulate cytokine production and symptoms. These results support the use of chronotherapy in PMR management and highlight the importance of circadian variations in inflammatory autoimmune diseases. Overall, the results effectively address the research question and provide valuable insights into the pathophysiology of PMR and the effects of prednisolone treatment.

underlying the observed diurnal variations in symptoms and biomarkers. Despite these limitations, the study provides valuable insights into the diurnal patterns of symptoms and biomarkers in PMR and supports the use of chronotherapy in disease management. However, larger studies with longer follow-up periods and comprehensive assessments of confounding factors are needed to confirm these findings and address potential limitations. Overall, the limitations are appropriately acknowledged and discussed in the context of the study findings.

Gandhi et al. (2021)	The research question is comprehensive, focusing on the prevalence, causes, assessment methods, and treatment options for excessive daytime sleepiness.	The methodology appears robust, utilizing systematic searches of PubMed and MEDLINE databases and referencing the latest practice parameters from the American Academy of Sleep Medicine.	The data analysis involves evidence from the literature to provide an overview of EDS epidemiology, causes, assessment methods, and treatment options. A qualitative synthesis approach is expected.	The results offer a detailed overview of EDS epidemiology, causes, assessment methods, and treatment options, relevant for clinical practice.	Limitations may include biases in evidence selection and interpretation, variability in study methodologies, and the inability to capture the most recent evidence beyond the search cutoff date.
Garber (2016)	The research question is effectively addressed, focusing on the FDA approval of tofacitinib (Xeljanz), the first Janus kinase (JAK) inhibitor for rheumatoid arthritis, and its implications for the treatment landscape. The article explores the novelty of JAK inhibition in rheumatoid arthritis therapy and provides	The methodology involves a detailed analysis of various factors related to Xeljanz's approval and market dynamics. The article draws upon clinical trial data, expert opinions from rheumatologists and analysts, sales projections, and government documents obtained through the US Freedom of Information	The data analysis involves synthesizing information from clinical trials, expert opinions, sales projections, and government documents to evaluate Xeljanz's efficacy, safety, market potential, and pricing strategy. Key findings, such as efficacy comparisons with TNF inhibitors, sales projections, and pricing	The results section provides a detailed overview of key findings related to Xeljanz's approval, efficacy, safety profile, market potential, and pricing strategy. These findings include clinical trial outcomes, expert opinions, sales projections, and insights into the drug's development history. Key points, such as	The article acknowledges several limitations inherent in Xeljanz's approval and market entry. These limitations include potential third-party payer resistance due to high pricing, uncertainties regarding long-term safety profiles, and concerns about herpes zoster infections and other adverse effects.

insights into the drug's development, efficacy, safety profile, market potential, and pricing strategy. By analyzing these aspects, the research question comprehensively evaluates the impact of Xeljanz on rheumatoid arthritis treatment and its positioning relative to existing therapies. Overall, the research question is relevant, timely, and well-articulated, covering various dimensions of Xeljanz's approval and market entry.

Act to provide a comprehensive overview. While the methodology lacks explicit details on data collection procedures, it effectively synthesizes information from multiple sources to address key aspects of Xeljanz's approval and market outlook. Additionally, the inclusion of perspectives from diverse stakeholders enhances the credibility and depth of analysis. Overall, the methodology is appropriate for exploring the research question and provides valuable insights into Xeljanz's regulatory pathway, development history, and market positioning.

considerations, are supported by relevant data and expert insights. While the article does not present statistical analyses in the traditional sense, it effectively analyzes qualitative and quantitative information to draw meaningful conclusions. The data analysis is thorough, comprehensive, and aligns with the objectives of the research question, providing valuable insights into various aspects of Xeljanz's approval and market entry.

Xeljanz's comparable efficacy to TNF inhibitors, its gradual market adoption, potential sales projections, and pricing considerations, are clearly presented and supported by relevant data and expert commentary. Overall, the results section effectively summarizes the main outcomes of the research and provides valuable insights into the implications of Xeljanz's approval for rheumatoid arthritis treatment.

Additionally, the article discusses challenges related to market adoption, physician comfort with the new therapy, and the need for further real-world usage and experience. By addressing these limitations, the article provides a balanced perspective on Xeljanz's prospects and underscores the importance of ongoing monitoring and research to assess its long-term safety and effectiveness. Overall, the discussion of limitations enhances the credibility and completeness of the analysis, highlighting areas for future research and consideration in clinical practice.

Gavilán-Carrera et al. (2022)	The research question addresses an important gap in understanding the effects of aerobic exercise on patient-reported outcomes (PROs) in women with systemic lupus erythematosus (SLE) and whether changes in cardiorespiratory fitness (CRF) mediate these effects. It is clear, specific, and relevant to improving rehabilitation strategies for SLE patients.	The study utilized a non-randomized clinical trial design, which may introduce selection bias and limit the generalizability of the findings. However, the intervention group underwent 12 weeks of aerobic exercise sessions, while the control group received usual care, enhancing the internal validity of the study. PROs were assessed using validated measures, and cardiorespiratory fitness was objectively measured using the Bruce test. Overall, the methodology is appropriate for evaluating the effects of aerobic exercise on PROs and CRF in women with SLE.	Data analysis involved comparing changes in PROs and CRF between the exercise group (EG) and control group (CG) using appropriate statistical methods, including mean differences and mediation analysis. The study found significant reductions in general fatigue and physical fatigue in the EG compared to the CG, with changes in CRF mediating the effects on general fatigue. The data analysis appears thorough and aligns with the research objectives.	The results indicate that 12 weeks of progressive aerobic exercise may lead to improvements in certain dimensions of fatigue in women with SLE, particularly general fatigue and physical fatigue. However, no significant differences were observed in other PROs, including psychological stress, sleep quality, depressive symptoms, and quality of life. Changes in CRF were found to mediate the effects of exercise on general fatigue. Overall, the results provide valuable insights into the potential benefits of aerobic exercise in managing fatigue in SLE patients.	Several limitations should be considered, including the non-randomized design, which may introduce selection bias and limit causal inference. The study's sample size was relatively small, potentially impacting the statistical power and generalizability of the findings. Additionally, the study only included women with SLE, limiting the extrapolation of results to other populations. Future research with larger sample sizes and randomized controlled designs is warranted to confirm the findings and address these limitations.

Gezer et al. (2017)	The research question addresses the effects of psoriatic arthritis (PsA) on sleep quality and explores the association between sleep quality and clinical parameters, quality of life, and psychological state in patients with PsA. It is clear and specific, focusing on an important aspect of PsA management that has not been extensively studied. By investigating the relationship between PsA and sleep quality, the study aims to provide valuable insights into the impact of the disease on patients' overall well-being. Overall, the research question is relevant and aligned with the objectives of the study.	The methodology involves recruiting 41 patients with PsA and 38 healthy volunteers and assessing sleep quality using the Pittsburgh Sleep Quality Index (PSQI). Additionally, anxiety and depression are evaluated using the Hospital Anxiety and Depression Scale (HADS), while PsA-specific parameters such as PsA Quality of Life (PsAQoL) Index, Psoriasis Area and Severity Index (PASI), and visual analogue scale (VAS) for generalized pain are measured. The methodology is comprehensive and appropriate for investigating the association between PsA and sleep quality, quality of life, and psychological	The data analysis includes comparisons of sleep quality between patients with PsA and healthy controls using the PSQI and correlations between PSQI scores and various clinical parameters, including anxiety, pain, PsAQoL scores, and inflammatory markers. Multiple regression analysis is also performed to identify independent associations between PSQI scores and clinical parameters. The analysis is thorough and provides valuable insights into the relationships between PsA and sleep quality, anxiety, pain, and disease activity. However, more detailed reporting of	The results indicate that patients with PsA have significantly lower sleep quality compared to healthy controls, as evidenced by higher PSQI scores across various domains. Moreover, PSQI scores correlate with anxiety, generalized pain, PsAQoL scores, enthesitis, and inflammatory markers such as C-reactive protein (CRP) and erythrocyte sedimentation rate (ESR). Multiple regression analysis further highlights the independent association between PSQI scores and ESR levels. These findings suggest that sleep quality in PsA patients is influenced by	The abstract acknowledges several limitations, including the relatively small sample size, which may limit the generalizability of the findings. Additionally, the cross-sectional design of the study precludes the establishment of causal relationships between PsA and sleep quality or other clinical parameters. The methodology does not provide details on potential confounders or adjustments made in the analysis, which may affect the interpretation of results. Furthermore, the study relies on self-reported measures of sleep quality, anxiety, and depression, which may be subject to

		state. However, the sample size is relatively small, and additional details on recruitment methods and potential confounders would enhance the study's rigor.	statistical methods and effect sizes would improve the transparency of the analysis. Overall, the data analysis is appropriate for addressing the research question and objectives of the study.	various clinical parameters and disease activity markers. Overall, the results effectively address the research question and provide valuable insights into the impact of PsA on sleep quality and associated factors.	recall or reporting bias. Despite these limitations, the study provides valuable insights into the association between PsA and sleep quality, highlighting areas for further research and potential interventions to improve sleep outcomes in PsA patients. Overall, the limitations are appropriately acknowledged and discussed in the context of the study findings.
Gharib et al. (2021)	The research question is clearly defined and relevant, aiming to assess the efficacy and safety of metformin in rheumatoid arthritis patients receiving	The methodology is appropriate, employing a prospective, randomized, controlled, single-blinded study design. This design helps to minimize bias and	The data analysis appears appropriate, utilizing statistical comparisons to assess outcomes between the metformin and control groups. By analyzing	The results of the study demonstrate significant improvements in inflammation, disease severity, and QOL in RA patients treated with metformin	Despite its strengths, the study has limitations, including the dropout of some participants due to intolerance to metformin side effects

	conventional synthetic disease-modifying anti-rheumatic drugs. This question addresses a gap in the literature and has practical implications for managing RA.	allows for the comparison of outcomes between the metformin group and the control group. However, it's worth noting that blinding of participants and researchers could strengthen the study further.	serum C-reactive protein, disease activity of 28 joints based on CRP (DAS-28-CRP), quality of life, and serum adiponectin levels, the study provides a comprehensive evaluation of metformin's effects on various parameters relevant to RA.	compared to the control group. The reduction in CRP levels and DAS-28-CRP scores, along with the improvement in QOL, suggests a beneficial effect of metformin in managing RA symptoms. However, the unexpected reduction in serum adiponectin levels in the metformin group warrants further investigation and discussion.	and non-compliance. Additionally, the relatively small sample size may limit the generalizability of the findings. Furthermore, longer-term follow-up could provide insights into the sustained effects and safety profile of metformin in RA patients.
Goes et al. (2017)	The research question is clearly stated: to investigate the associations of sleep quality with pain, depression, and disease activity in rheumatoid arthritis (RA) patients. The question is relevant and addresses an important aspect of RA management and quality of life.	The methodology involves a cross-sectional observational study of RA patients, utilizing standardized measures to assess sleep quality, daily sleepiness, depression, and risk of sleep apnea. Data collection also includes epidemiological, clinical, serological, and treatment information. The study design allows	The data analysis appears appropriate for the study objectives, utilizing descriptive statistics and both univariate and multivariate analyses to examine associations between sleep quality and various factors. Statistical tests are used to assess significance levels and identify independent predictors	The results are presented clearly, providing information on the prevalence of poor sleep quality in RA patients and associations with pain, depression, and risk of sleep apnea. The inclusion of both univariate and multivariate analyses aids in understanding the factors	The study acknowledges several limitations, including its cross-sectional design, which limits causal inference. The reliance on self-reported measures may introduce bias, and potential confounders such as medication usage or comorbidities are not fully addressed.

		for the examination of associations between sleep quality and various factors. However, the cross-sectional nature of the study limits causal inference, and potential confounders may not be fully accounted for. Additional details on participant selection and recruitment procedures would enhance transparency.	of sleep impairment. However, further details on the specific statistical methods used and adjustments for potential confounders would enhance the robustness of the findings.	independently associated with sleep impairment. However, more information on effect sizes and confidence intervals would provide a clearer interpretation of the results.	Additionally, the study's sample size may affect the generalizability of findings. Further discussion on these limitations and their implications for interpretation would strengthen the discussion section.
Goulabchand et al. (2022)	The research question aimed to investigate neurocognitive profiles in pSS patients with cognitive complaints and explore relationships with fatigue, depression, sleep disorders, and quality of life. The study effectively addressed these objectives by conducting extensive neuropsychological	Strengths: The study used a prospective design with consecutive patient inclusion and comprehensive neuropsychological testing. It employed validated diagnostic criteria for pSS and included a broad range of assessments (e.g., cognitive tests, disease activity indices, psychiatric evaluations). Brain MRIs were offered	The data analysis utilized appropriate statistical methods (e.g., descriptive statistics, chi-square tests, correlation analyses) to explore relationships between cognitive profiles, clinical parameters, and quality of life measures. However, due to the small sample size, caution is warranted in interpreting statistical	The study provided detailed insights into neurocognitive impairments in pSS patients, highlighting prevalent memory and executive function deficits. It effectively linked cognitive complaints with clinical manifestations such as fatigue, depression, and sleep disorders. The findings underscored the	Internal Validity: The study's internal validity was supported by rigorous inclusion criteria, standardized assessments, and statistical analyses. However, potential biases (e.g., selection bias, measurement bias) and the exclusion of patients with comorbidities may limit the findings'

	assessments and correlating findings with various clinical parameters.	to a subgroup for additional insights.	significance. The inclusion of thresholds for defining pathological cognitive scores and the integration of multiple clinical parameters strengthened the analysis.	multidimensional impact of cognitive impairments on patient well-being and quality of life.<br>While informative, the results may not apply universally to all pSS patients due to the small sample and single-center nature. The interpretation of cognitive test results could be subjective, despite attempts to standardize assessments. Additionally, the study did not investigate underlying mechanisms (e.g., biomarkers, neuroimaging) driving cognitive dysfunction in pSS.	generalizability.<br>External Validity: The study's external validity is constrained by its single-center design and relatively small sample size. Therefore, caution is needed in applying findings to broader populations of pSS patients.<br>Methodological Limitations: The reliance on self-reported measures for some assessments (e.g., quality of life, fatigue) and the lack of longitudinal data limit the study's ability to establish causal relationships or long-term outcomes.
Griffin et al. (2021)	The research question is well-defined: to examine the relationship between sleep disturbance and pain over a 14-year	The methodology involves secondary data analysis using waves of the Health and Retirement Study (HRS), a longitudinal	The data analysis appears appropriate for the study objectives, utilizing random intercept cross-lagged panel modeling (RI-	The results are presented clearly, providing evidence of reciprocal effects between sleep disturbance and pain	The study acknowledges several limitations, including its reliance on self-report measures of sleep disturbance and

period among adults over age 50. The question is relevant and addresses an important aspect of health and aging, providing insights into the bidirectional relationship between sleep and pain in older individuals.	observational study of U.S. adults over age 50. Sleep disturbance and pain were assessed via self-report measures, and random intercept cross-lagged panel modeling (RI-CLPM) was employed to analyze the bidirectional relationship between sleep disturbance and pain over two-year intervals. The study design allows for the examination of longitudinal associations while accounting for the stable trait-like nature of both factors. However, the reliance on self-report measures may introduce bias, and the study's generalizability may be limited to older adults in the United States. Further details on potential sources of bias and missing data handling would enhance transparency.	CLPM) to examine the bidirectional relationship between sleep disturbance and pain over time. Statistical significance levels are reported, and adjustments are made for baseline covariates. However, more information on the specific statistical methods used and model fit indices would enhance the transparency of the analysis.	across some intervals over a 14-year period among adults over age 50. The inclusion of latent variables representing the trait-like nature of sleep disturbance and pain enhances the understanding of stable differences between individuals. Additionally, adjustments for baseline covariates strengthen the robustness of the findings. However, more information on effect sizes and potential clinical implications would provide a deeper interpretation of the results.	pain, which may be subject to recall bias. The generalizability of findings may be limited to older adults in the United States participating in the HRS. Additionally, potential confounders such as medication usage or comorbidities are not fully addressed. Further discussion on these limitations and their implications for interpretation would strengthen the discussion section.

Guagnano et al. (2021)	The research question addresses the impact of a specific dietary intervention on RA symptoms and overall health in patients already receiving optimized drug treatment. This is relevant and timely, considering the interest in non-pharmacological approaches to managing RA symptoms. Understanding the potential benefits of dietary modifications can enhance treatment strategies and improve patient outcomes.	The methodology involves a randomized controlled trial with a clear comparison between an exclusionary diet and a balanced control diet. Patients are appropriately assigned to dietary groups and assessed using standardized measures. However, the study lacks details on randomization procedures and blinding methods, which could affect the validity of results. Nonetheless, the study design allows for a systematic evaluation of the dietary intervention's effects on RA symptoms and health outcomes.	Data analysis appears robust, with appropriate statistical techniques used to compare outcomes between dietary groups and over time. Results are presented clearly, highlighting significant findings and trends observed during the study period. However, additional information on specific statistical tests used and adjustments for potential confounders would enhance the transparency and rigor of the analysis. Overall, the data analysis effectively addresses the study objectives and provides valuable insights into the dietary intervention's effects.	The results demonstrate significant improvements in pain levels, overall health, and quality of life among patients on the exclusionary diet compared to the control group. Key health markers, including weight, blood pressure, and inflammation markers, also showed positive changes in response to the dietary intervention. These findings support the potential benefits of dietary modifications in managing RA symptoms and enhancing overall health outcomes.	Limitations include the lack of detailed information on randomization and blinding procedures, which could introduce bias into the study. Additionally, the study's relatively small sample size and short duration may limit the generalizability of findings and the ability to detect long-term effects of the dietary intervention. Future research with larger sample sizes and longer follow-up periods could address these limitations and provide further insights into dietary management of RA.
Guan et al. (2020)	The research question addresses the effect of hydroxychloroquine (HCQ) on sleep disturbance in patients	The methodology involves enrolling and following up with 383 pSS patients to assess sleep quality using the	The data analysis involves comparing baseline characteristics and outcomes between good sleep and poor	The results indicate that long-term use of HCQ is associated with a reduced risk of sleep disturbance in pSS	The abstract identifies several limitations, including the retrospective nature of the study, which may

with primary Sjögren's syndrome (pSS), which is relevant and clearly stated. The study aims to investigate the relationship between HCQ use and sleep quality in pSS patients, filling a gap in the literature regarding the impact of HCQ on sleep disorders in this population. By evaluating the Pittsburgh Sleep Quality Index (PSQI) and other relevant measures, the study seeks to provide insights into the potential benefits of HCQ in improving sleep quality and overall quality of life in pSS patients. Overall, the research question is well-defined and aligned with the study objectives.

PSQI and quality of life using the World Health Organization Quality of Life Brief Version (WHOQOL-BREF) scale and European League Against Rheumatism Sjögren's Syndrome Patients Reported Index (ESSPRI). Patients were divided into good sleep and poor sleep groups based on PSQI scores, and risk factors for sleep disorders were analyzed using univariate and multivariate analysis. Additionally, patients were divided into HCQ-administered and non-administered groups to compare baseline characteristics and outcomes. The methodology is comprehensive and appropriate for investigating the research question. However, additional details on data collection methods, follow-up

sleep groups, as well as between HCQ-administered and non-administered groups. Descriptive statistics are used to summarize the prevalence of sleep disturbance and HCQ use, while univariate and multivariate analyses are employed to identify risk factors for sleep disorders. The analysis also includes comparisons of PSQI scores, ESSPRI, and QoL between HCQ-administered and non-administered groups. Overall, the data analysis is appropriate for addressing the research question, although additional statistical methods or adjustments for potential confounders could strengthen the analysis.

patients. The study reports a significant difference in sleep quality between HCQ-administered and non-administered groups, as well as improvements in PSQI scores, ESSPRI, and QoL in the HCQ-administered group. The findings suggest that HCQ may have a beneficial effect on sleep quality and overall well-being in pSS patients. However, the results could be enhanced with clearer delineation of findings from individual analyses and more detailed reporting of effect sizes or measures of association. Overall, the results effectively address the study objectives and provide valuable insights into the potential benefits of HCQ in pSS patients.

introduce bias and limit the ability to establish causality. Additionally, the methodology does not provide details on potential confounders or adjustments made in the analysis, which may affect the interpretation of results. The inclusion of only pSS patients may limit the generalizability of the findings to other populations. Furthermore, the study relies on self-reported measures of sleep quality and quality of life, which may be subject to recall or reporting bias. Despite these limitations, the study provides valuable insights into the potential benefits of HCQ in improving sleep quality and overall well-being in

		procedures, and potential confounders would enhance the study's rigor and reliability.			pSS patients. However, cautious interpretation is warranted due to the inherent limitations of the study design and potential confounding factors.
Gudbergsen et al. (2019)	The research question is well-defined and relevant to the management of patients with overweight/obesity and knee osteoarthritis (KOA). It aims to determine the efficacy and safety of liraglutide in inducing weight loss and alleviating knee pain in this population. The question addresses an important gap in current treatment options for individuals with both conditions.	The methodology is robust, employing a randomized controlled trial design to investigate the effects of liraglutide on weight loss and knee pain in patients with KOA and overweight/obesity. The inclusion criteria are clearly specified, and the intervention consists of a pre-random assignment diet followed by liraglutide or placebo treatment. However, additional details on the randomization	The data analysis appears thorough, with appropriate statistical comparisons between the liraglutide and placebo groups for primary and secondary outcomes (i.e., changes in body weight and Knee injury and Osteoarthritis Outcome Score (KOOS) pain subscale). Confidence intervals and p-values are provided to quantify the magnitude and	The results indicate a significant reduction in body weight with liraglutide compared to placebo over the 52-week treatment period. However, there was no significant difference in KOOS pain scores between the two groups. These findings suggest that liraglutide effectively induces weight loss but does not alleviate knee pain in patients with KOA and overweight/obesity. The conclusions are supported by the	The study acknowledges several limitations, including the lack of long-term follow-up beyond 52 weeks, potential biases related to the pre-random assignment diet intervention, and the relatively small sample size. Additionally, the study population may not fully represent all patients with KOA and overweight/obesity, limiting the generalizability of the findings. Addressing these limitations would strengthen the

		process, blinding procedures, and potential confounders would strengthen the methodology section.	significance of the observed effects. However, further information on statistical methods and adjustments for potential confounders would enhance transparency.	reported effect sizes and confidence intervals.	validity and applicability of the study results.
Gudbjörnsson et al. (1993)	The research question is clearly defined: to investigate the sleeping habits and disturbances in outpatients with primary Sjögren's syndrome (pSS) compared to rheumatoid arthritis (RA) and healthy controls. The question is relevant, addressing an important aspect of quality of life in autoimmune diseases.	The methodology involves a comparative study utilizing a standardized sleep questionnaire and polysomnography to assess sleep disturbances in patients with pSS, RA, and healthy controls. The inclusion of both subjective (questionnaire) and objective (polysomnography) measures strengthens the study's validity. However, further details on participant selection,	The data analysis appears appropriate for the study design, utilizing statistical tests to compare sleep deficit and disturbances between patient groups. The use of p-values to denote statistical significance is standard practice, aiding in the interpretation of results. However, additional information on the specific statistical tests used and adjustments for	The results are presented clearly, detailing significant differences in sleep deficit and disturbances between patients with pSS, RA, and healthy controls. The inclusion of percentages and statistical significance enhances the understanding of the magnitude of differences observed. Additionally, the findings from polysomnography	The study acknowledges several limitations, including the relatively small sample size, which may limit the generalizability of the findings. Additionally, the use of polysomnography in a subset of patients may introduce selection bias. The study does not address potential confounding factors such as medication usage or comorbidities, which

		matching criteria, and data collection procedures would enhance transparency.	multiple comparisons would provide clarity.	provide objective evidence supporting the questionnaire results.	could influence sleep disturbances. Further discussion on these limitations and their implications for the interpretation of results would strengthen the discussion section.
Gumenyuk et al. (2012)	The study aims to investigate the relationship between disturbances of sleep and wakefulness in shiftworkers and the degree of mismatch between their endogenous circadian rhythms and the night-work schedule. It seeks to determine whether such mismatches contribute to the development of shift work disorder.	The study employs a well-designed methodology involving the recruitment of both asymptomatic night workers and night workers meeting diagnostic criteria for SWD. Participants are admitted to a sleep center, where circadian phase is determined through the assessment of salivary melatonin onset. Objective sleepiness is assessed using the multiple sleep	Data analysis involves comparing DLMO, sleep patterns, objective sleepiness (MSLT), and exposure to morning light between ANWs and those with SWD. Statistical tests, such as the Mann-Whitney U test, are used to assess differences between groups. Correlation analysis is conducted to explore relationships between DLMO and insomnia severity. The analysis provides	The study findings reveal that asymptomatic night-shift workers have an internal physiological delay of the circadian pacemaker, as evidenced by later DLMO compared to those with SWD. Individuals with SWD maintain a circadian phase position similar to day workers, leading to a mismatch/conflict between their endogenous rhythms	The study concludes that internal physiological delays of the circadian pacemaker in asymptomatic night-shift workers may contribute to a better adaptation to night work. In contrast, individuals with SWD experience a mismatch between their endogenous rhythms and their sleep-wake schedule, leading to sleep disturbances and

		latency test. Exposure to morning light is also measured. Statistical analyses, including the Mann-Whitney U test, are used to evaluate differences between groups. This methodology provides valuable insights into the circadian rhythms and sleep patterns of shiftworkers.	quantitative insights into circadian phase delays and their association with sleep disturbances and SWD.	and their sleep-wake schedule. Additionally, DLMO is significantly correlated with insomnia severity, indicating that workers with more severe insomnia symptoms have an earlier timing of DLMO. These results highlight the importance of circadian phase alignment in the development of SWD.	more severe insomnia symptoms. Exposure to morning light appears to play a role in this mismatch. These findings underscore the importance of circadian alignment in promoting sleep health among shiftworkers.
Gundogdu et al. (2021)	The research question on the prevalence and factors associated with OSA in SSc patients is clearly defined and clinically relevant.	Strengths: Cross-sectional design, clear inclusion criteria, ethical approval, use of PSG for OSA diagnosis. Weaknesses: Small sample size, potential selection bias, lack of control group.	Strengths: Detailed descriptive and inferential statistics, appropriate statistical tests. Weaknesses: Small sample size affecting statistical power, limited multivariate analysis.	Strengths: Clear presentation of findings, relevance of clinical variables examined. Weaknesses: Limited generalizability, lack of outcome measures related to treatment response.	Internal Validity: Potential selection and measurement biases. External Validity: Small sample size, single-center study. Methodological Considerations: Cross-sectional design limitations.
Gutiérrez et al. (1999)	The study aims to compare cortisol and prolactin responses to specific stimuli in patients with active rheumatoid arthritis	The study uses cortisol response to insulin-induced hypoglycemia and PRL response to thyrotropin-releasing hormone in patients with	Statistical tests compare basal cortisol and PRL levels, peak values, and integrated responses (AUC) between RA and controls, revealing	Basal cortisol and PRL levels did not significantly differ between groups, but the RA group showed lower cortisol response	The study concludes that active RA is associated with subtle dysfunction of the HPA axis, specifically in cortisol response

	and controls, shedding light on potential dysfunctions in the hypothalamic-pituitary-adrenal axis in RA.	RA and paired controls, analyzing differences in cortisol and PRL levels between groups.	subtle HPA axis dysfunction in RA based on cortisol response patterns.	during hypoglycemia at the 30-45 min interval. Peak cortisol and PRL levels were similar between groups, indicating normal PRL secretion.	patterns, while PRL secretion remains normal. Further research is needed to understand the mechanisms underlying HPA axis dysfunction in RA.
Guyon et al. (2014)	The research question is well-defined and relevant, examining the impact of restricted sleep on the daytime profiles of ACTH and cortisol concentrations. The study aims to explore the mechanisms underlying the link between insufficient sleep and increased cardiometabolic risk, focusing on alterations in the hypothalamic-pituitary-adrenal (HPA) axis.	The methodology is appropriate for the research question, utilizing a randomized crossover design with controlled sleep conditions and comprehensive hormonal measurements. The use of polygraphic sleep recording and frequent blood and saliva sampling provides detailed data on ACTH and cortisol profiles. The assessment of perceived stress, hunger, and appetite adds valuable context to the hormonal data. However, the small sample size (13 subjects) is a limitation that could	The data analysis is thorough, examining overall levels, pulse frequency, and time-of-day variations of ACTH and cortisol. The study employs appropriate statistical methods to determine the significance of changes in hormone levels and their correlations with sleep loss and other variables. The analysis effectively highlights the differential impact of sleep restriction on morning versus evening hormone levels and the dampening of the circadian cortisol decline.	The results indicate that sleep restriction leads to increased overall ACTH and cortisol levels, with significant time-of-day variations. Morning ACTH levels are elevated without a corresponding increase in cortisol, while evening cortisol levels are markedly increased, dampening the circadian decline. The study also finds that sleep restriction increases appetite, correlated with elevated cortisol levels. These findings suggest that insufficient sleep disrupts HPA axis	Limitations include the small sample size, which may limit the statistical power and generalizability of the findings. The short duration of the study (two nights of restricted sleep) does not capture long-term effects of chronic sleep restriction. The study does not explore other potential confounding factors, such as variations in individual stress responses or baseline HPA axis activity. Additionally, the study participants' usual sleep patterns and overall health

		affect the generalizability of the findings.		activity, potentially contributing to cardiometabolic risk.	status are not detailed, which could influence the results.
Haack et al. (2007)	The research question is well-defined: to investigate the effects of reduced sleep duration on peripherally circulating inflammatory mediators and the relationship between inflammation and increased pain in response to prolonged sleep restriction. The question addresses an important gap in understanding the interrelationships between sleep, pain, and inflammation, particularly in medical conditions where these factors co-occur.	The methodology involves a randomized, controlled in-laboratory study conducted over 16 days, with participants randomly assigned to either 12 days of sleeping 8 hours per night or 4 hours per night. Mood and pain symptoms were assessed throughout the experimental days, and urine and blood samples were collected for the analysis of inflammatory markers. The study design allows for the examination of causal relationships between sleep duration, inflammation, and pain. However, the small sample size may limit the generalizability of findings, and potential confounders such as medication usage or comorbidities are not	The data analysis appears appropriate for the study objectives, utilizing statistical tests to compare levels of inflammatory markers between the two sleep conditions and assess associations between inflammation and pain ratings. Statistical significance levels are reported, aiding in the interpretation of results. However, more information on effect sizes and potential confounders would provide a deeper understanding of the findings.	The results are presented clearly, demonstrating elevated levels of IL-6 in the 4-hour sleep condition compared to the 8-hour sleep condition. Although CRP levels showed a similar trend, the difference was not statistically significant. Levels of sTNF-R p55, PG E2, and 11beta-F2alpha metabolite did not differ significantly between groups. Elevated IL-6 levels were strongly associated with increased pain ratings in response to sleep restriction. The inclusion of associations between inflammatory markers and pain ratings strengthens the understanding of the	The study acknowledges several limitations, including its small sample size and reliance on self-report measures of pain and mood symptoms. The generalizability of findings may be limited to healthy adults in a controlled laboratory setting, and potential confounders such as medication usage or comorbidities are not fully addressed. Additionally, the study's focus on acute sleep restriction may not fully capture the complex interactions between sleep, inflammation, and pain in chronic conditions. Further discussion on these

		fully addressed. Further details on participant selection criteria and blinding procedures would enhance transparency.		relationship between sleep, inflammation, and pain. However, more information on potential mechanisms underlying these associations would provide additional insights.	limitations and their implications for interpretation would strengthen the discussion section.
Hartescu et al. (2015)	The research question aims to investigate whether increasing physical activity to the level recommended in public health guidelines can improve sleep quality among inactive adults with insomnia. It is well-defined and addresses an important gap in the literature regarding the minimum levels of physical activity required to enhance sleep outcomes. The question aligns with the objectives of the study and provides	The study employs a two-armed parallel randomized controlled trial design, which is appropriate for assessing the effects of increasing physical activity on sleep quality. A total of 41 participants meeting research diagnostic criteria for insomnia were randomized to either a physical activity intervention group or a control group. The intervention involved a monitored program of ≥150 minutes of moderate- to vigorous-intensity physical	Data analysis involves comparing changes in insomnia severity, mood, fatigue, and daytime sleepiness between the physical activity group and the control group at 6 months post-baseline. The results are presented clearly, indicating a significant reduction in insomnia symptom severity, depression, and anxiety scores in the physical activity group compared to the control group. Statistical tests (e.g.,	The results demonstrate that increasing physical activity to internationally recommended minimum levels significantly improves both daytime and nighttime symptoms of chronic insomnia. Specifically, the physical activity group showed a significant reduction in insomnia symptom severity, depression, and anxiety scores compared to the control group at 6 months post-baseline.	While the study design is robust, some limitations should be considered. The sample size is relatively small, which may limit the generalizability of the findings. Additionally, the study primarily focuses on adults meeting research diagnostic criteria for insomnia, and the results may not be applicable to individuals with other sleep disorders or those without insomnia.

valuable insights into the potential benefits of physical activity for managing insomnia symptoms. Overall, the research question is clear, relevant, and contributes to advancing knowledge in the field of sleep medicine.	activity per week for 6 months. Outcome measures included the Insomnia Severity Index, mood, fatigue, and daytime sleepiness, assessed at baseline and 6 months post-baseline. Activity and light exposure were monitored throughout the trial using accelerometry and actigraphy. The methodology appears robust, with clear intervention protocols and appropriate outcome measures to evaluate the impact of physical activity on sleep outcomes.	ANOVA) are appropriately used to assess between-group differences, and effect sizes are provided to quantify the magnitude of changes. The analysis also explores the independence of changes from daily light exposure. Overall, the data analysis appears rigorous and effectively supports the study findings.	These improvements were independent of daily light exposure levels. However, no significant effect on daytime fatigue was observed. The findings are presented clearly and supported by statistical analyses, contributing to the understanding of the relationship between physical activity and sleep outcomes in individuals with insomnia. Overall, the results provide valuable insights into the potential benefits of physical activity for managing insomnia symptoms.	Furthermore, the study duration of 6 months may not capture long-term effects of physical activity on sleep outcomes. Additionally, the reliance on self-reported measures and the lack of objective sleep measures (e.g., polysomnography) may introduce bias. Despite these limitations, the study provides important preliminary evidence supporting the role of physical activity in improving sleep quality among individuals with insomnia. Future research with larger sample sizes and longer follow-up periods is warranted to confirm these findings and address potential limitations.

He et al. (2016)	The research question addresses an important gap in understanding the role of dietary patterns in rheumatoid arthritis (RA) susceptibility among Chinese populations. The study design allows for the assessment of dietary factors as potential risk or protective factors for RA development.	The study employs a large-scale case-control design involving 968 patients with RA and 1037 matched healthy controls recruited from 18 teaching hospitals, enhancing the generalizability of the findings. The use of a self-administered questionnaire to collect socio-demographic characteristics and dietary intakes 5 years prior to RA onset may introduce recall bias and impact data accuracy. However, the application of multiple logistic regression analysis strengthens the identification of independent dietary risk factors for RA susceptibility.	Data analysis includes Student's t-test to compare consumption differences between cases and controls, providing insight into dietary patterns associated with RA. Multiple logistic regression analysis is used to identify independent dietary risk factors for RA susceptibility, controlling for potential confounders. While the findings suggest protective effects of certain dietary items such as mushrooms, citrus fruits, and dairy products, further exploration of dietary interactions and confounding factors could enhance the analysis.	The results indicate decreased consumption of several dietary items among RA patients compared to healthy controls, including mushrooms, beans, citrus fruits, poultry, fish, edible viscera, and dairy products. Multivariate analyses reveal protective effects of mushrooms, citrus fruits, and dairy products on RA development, highlighting the potential role of dietary interventions in reducing RA risk.	The conclusion underscores the significance of dietary factors in RA susceptibility among Chinese populations and suggests the potential for dietary interventions to mitigate RA risk. The identification of specific dietary items with protective effects provides actionable insights for public health initiatives aimed at reducing RA incidence. However, further research is warranted to elucidate the mechanisms underlying these associations and validate the effectiveness of dietary interventions in RA prevention.

Heffner et al. (2011)	The research question is well-defined: to examine the associations between sleep disturbance and circulating levels of the inflammatory cytokine interleukin-6 (IL-6) in individuals with and without chronic low back pain (CLBP). The question addresses an important gap in understanding the concurrent relationships among chronic pain, sleep disturbance, and inflammation, particularly in the context of CLBP.	The methodology involves a cross-sectional study comparing adults with chronic low back pain (CLBP) to controls without chronic pain, matched for sex and age. Participants completed measures of sleep quality and depressive symptoms, provided a blood sample for IL-6 analysis, and reported morning pain levels. The study design allows for the examination of associations between sleep disturbance, IL-6 levels, and pain reports in individuals with and without CLBP. However, the cross-sectional nature of the study limits causal inference, and potential confounders such as medication usage or comorbidities are not fully addressed. Further details on participant recruitment and potential sources of bias would enhance	The data analysis appears appropriate for the study objectives, utilizing statistical tests to compare sleep disturbance, IL-6 levels, and pain reports between individuals with CLBP and controls. Additionally, associations between sleep quality, IL-6 levels, and pain reports are examined within the CLBP group. Statistical significance levels are reported, aiding in the interpretation of results. However, more information on effect sizes and potential confounders would provide a deeper understanding of the findings.	The results are presented clearly, demonstrating that individuals with CLBP had more sleep disturbance compared to controls. While circulating IL-6 levels were similar between groups, poorer sleep quality was associated with higher IL-6 levels and both sleep and IL-6 related to pain reports in the CLBP group. In contrast, controls showed normal age-related increases in IL-6 levels, and sleep quality was unrelated to IL-6 levels. Depressive symptoms could not fully explain the observed associations. The inclusion of associations between sleep quality, IL-6 levels, and pain reports strengthens the understanding of the complex relationships	The study acknowledges several limitations, including its cross-sectional design, which limits causal inference. The reliance on self-report measures of sleep quality, depressive symptoms, and pain may introduce bias, and potential confounders such as medication usage or comorbidities are not fully addressed. Additionally, the small sample size and lack of diversity in the participant population may limit the generalizability of findings. Further discussion on these limitations and their implications for interpretation would strengthen the discussion section.

		transparency.		among chronic pain, sleep disturbance, and inflammation. However, further exploration of potential mechanisms underlying these associations would provide additional insights.	
Hennessy et al. (2014)	The study investigates the prevalence of restless legs syndrome (RLS) among HIV-infected adults and examines the differences in demographic factors, clinical characteristics, and biomarkers related to dopamine, iron, and inflammation between those with and without RLS symptoms. This question is pertinent for understanding the interplay between RLS and HIV, potentially guiding better clinical management of these	The study employs a prospective longitudinal design with a sample of 316 HIV-positive adults evaluated using the International RLS Study Group criteria. Genes associated with dopamine, iron, and inflammation were selected for analysis. The methodology is robust, using established diagnostic criteria and focusing on a diverse set of biomarkers.	The analysis revealed that 11% of the sample met all four RLS diagnostic criteria, similar to the general population. Genetic analysis showed that carrying two copies of the minor allele for IL1B or the minor allele for IL17A increased the likelihood of meeting RLS diagnostic criteria. The statistical methods used are appropriate for identifying significant genetic associations.	The results indicate a comparable prevalence of RLS in HIV-infected adults to that in the general population, with specific genetic variations in cytokine genes (IL1B and IL17A) being associated with increased likelihood of RLS symptoms. These findings suggest an inflammatory component in the pathogenesis of RLS among HIV-infected individuals.	The study's cross-sectional nature limits causal inferences, and the sample size, although adequate, may not capture all genetic variations associated with RLS. Additionally, the study focuses on a specific population (HIV-infected adults), which may limit the generalizability of the findings to other groups. Longitudinal studies and larger samples are needed to confirm these associations and

	patients.				explore the mechanisms further.
Henshaw et al. (2021)	The research question is clearly defined and relevant, aiming to provide insight into cannabinoid-mediated pro- and anti-inflammatory cytokine responses in preclinical in vivo studies. This addresses an important aspect of cannabinoid pharmacology, considering the potential therapeutic applications of cannabinoids in modulating inflammation. The question is comprehensive, covering various cannabinoids and their effects on cytokine levels in animal models,	The methodology involves a systematic search of multiple databases to identify relevant preclinical in vivo studies evaluating the effects of cannabinoids on inflammation in animal models. The inclusion criteria are clearly specified, and the risk of bias assessment is conducted using the SYstematic Review Center for Laboratory animal Experimentation (SYRCLE) tool. The methodology is robust, allowing for a comprehensive analysis of the available evidence on cannabinoid-mediated cytokine responses. However,	The data analysis appears thorough, involving the extraction and synthesis of data from 26 eligible full-text articles. Pro-inflammatory cytokines such as tumor necrosis factor alpha, interleukin-1β, interleukin-6, and interferon gamma are examined, and their levels are assessed following treatment with various cannabinoids. The findings indicate a predominantly anti-inflammatory effect of cannabidiol (CBD), cannabigerol (CBG), and CBD+THC combination, while delta 9-tetrahydrocannabinol	The results of the systematic review demonstrate that CBD, CBG, and CBD+THC combination exert a predominantly anti-inflammatory effect in preclinical in vivo studies, whereas THC alone does not reduce pro-inflammatory cytokine levels. Furthermore, improvements in disease or disability are apparent in studies where CBD, CBG, or CBD in combination with THC were administered. These findings suggest the potential therapeutic utility of cannabinoids, particularly CBD and	The study acknowledges several limitations, including the relatively small number of eligible studies identified for inclusion, potential publication bias, and heterogeneity in study designs and cannabinoid formulations. Additionally, while the review provides valuable insights into cannabinoid-mediated cytokine responses, the extrapolation of findings to human clinical trials may require further validation. The limitations are appropriately discussed, enhancing the interpretation and generalizability of the results.

	providing valuable insights for potential clinical translation.	potential limitations related to publication bias and study heterogeneity should be considered.	(THC) alone does not reduce pro-inflammatory cytokine levels. The association between cannabinoid-induced anti-inflammatory response and disease severity is also explored.	CBG, in modulating inflammation across various pathophysiological processes.	
Higuchi et al. (2015)	The study aims to investigate the prevalence and severity of restless legs syndrome (RLS) in patients undergoing hemodialysis and identify associated risk factors. This addresses an important gap in the literature and has clinical significance for the management of RLS in this patient population.	The study enrolled 159 stable patients on maintenance hemodialysis and diagnosed RLS based on criteria proposed by the International Restless Legs Syndrome Study Group (IRLSSG). RLS severity was assessed using the IRLSSG Severity Scale. Potential risk factors were assessed using univariate and multivariate regression analyses. The methodology is robust and appropriate for	The data analysis reveals that RLS affected 22% of the study population. The RLS subgroup had a longer duration of hemodialysis, higher cardiothoracic ratio, and elevated levels of inflammatory and oxidative stress markers compared to the non-RLS subgroup. Serum 8-hydroxy-2'-deoxyguanosine (8-OHdG) level emerged as an independent risk factor for high RLS	The study confirms the high prevalence of RLS among hemodialysis patients and highlights the association between RLS severity and markers of oxidative stress, particularly serum 8-OHdG levels. These findings suggest a potential role of oxidative stress in the pathophysiology of RLS in hemodialysis patients and underscore the need for further research in this area.	One limitation is the cross-sectional design, which precludes causal inference and limits the ability to establish temporal relationships between RLS, oxidative stress markers, and other variables. Additionally, the study may have selection bias as it includes stable patients on maintenance hemodialysis, potentially

		achieving the research objectives.	severity scores. The statistical analysis is comprehensive and accounts for potential confounders, enhancing the reliability of the results.		underestimating the prevalence of RLS. Longitudinal studies are needed to confirm the findings and explore causal relationships.
Hinkka et al. (2017)	The research question is clearly stated: to evaluate the safety and effects of a new home treatment method, a whole-body cold mist treatment, on patients with chronic inflammatory arthritis. This question addresses a relevant issue in arthritis management and seeks to determine the potential benefits and safety of a novel treatment approach for pain relief and improved sleep quality in this population.	The study utilized a crossover design involving 121 voluntary patients with chronic inflammatory arthritis. Whole-body cold mist shower therapy was administered during 1-week rehabilitation periods. Pain, sleep quality, and mental status were assessed using standardized measures such as the Visual Analogue Scale (VAS) for pain and sleep quality, and the Depression Scale (DEPS) for mental status. Physiological	The data analysis involved comparisons of pain, sleep quality, and mental status between treatment and control periods using appropriate statistical tests such as paired t-tests and likelihood ratio tests. The results indicate significant differences in pain and a trend towards improved sleep quality during the treatment period, while mental status remained stable. The analysis appears robust and adequately addresses	The results demonstrate a significant difference in pain levels between treatment and control periods, suggesting that the whole-body cold mist treatment may offer pain relief for patients with chronic inflammatory arthritis. There is also a trend towards improved sleep quality during the treatment period, although this finding is not statistically significant. Mental status, as assessed by the DEPS, did not show significant differences between treatment periods. Overall, the results suggest that the	While the study provides valuable insights into the potential benefits of the whole-body cold mist treatment for chronic inflammatory arthritis, several limitations should be considered. These include the relatively short duration of the study, limited assessment of long-term effects, and potential for bias in self-reported measures such as pain and sleep quality. Additionally, the study population consisted of voluntary patients, which may introduce selection bias. Further research

		parameters, medication use, and side effects were also recorded. The methodology appears appropriate for evaluating the safety and effects of the treatment.	the research objectives.	treatment may be safe and beneficial for pain management at home.	with larger samples and longer follow-up periods is needed to confirm these findings and evaluate the clinical significance of the treatment effect.
Horsley-Silva et al. (2019)	The research question is well-defined and relevant. It addresses the association between GERD symptoms and sleep quality in patients with systemic sclerosis, highlighting a significant clinical concern for this patient population.	The study uses a robust methodology, including a large sample size and validated patient-reported questionnaires (such as the Pittsburgh Sleep Quality Index and the gastrointestinal tract instrument). It employs multivariable logistic regression to control for confounding variables.	Data analysis is thorough and appropriate. The use of multivariable logistic regression helps identify independent associations between GERD symptoms and poor sleep quality. Statistical significance is reported, enhancing the credibility of the findings.	The results clearly demonstrate a significant association between GERD symptoms and poor sleep quality in SSc patients. The study provides detailed statistics, including odds ratios and confidence intervals, which support the robustness of the findings.	The study acknowledges several limitations, including the potential for recall bias in patient questionnaires, the lack of objective physiologic testing for GERD, and the inability to establish causality. Despite these limitations, the study provides valuable insights into the issue.
Hunt et al. (2021)	The research question is clearly defined,	The methodology involves a randomized,			The study may have several limitations,

aiming to investigate whether trait positive affect (PA) moderates the associations between sleep disruption and stimulated monocyte production of proinflammatory cytokines (TNF and IL-6) in good sleeping adults. This addresses an important aspect of understanding the potential protective factors against the inflammatory response to sleep disturbances, which is relevant for the prevention of adverse health consequences associated with insufficient sleep. The question is comprehensive, considering both trait positive affect and its interaction with sleep disruption in predicting cellular inflammation.

within-subjects crossover experiment comparing the effects of two nights of sleep disruption versus two nights of uninterrupted sleep in good sleeping adults. Stimulated monocytic production of proinflammatory cytokines TNF and IL-6 was assayed using flow cytometric methods. Linear mixed effects models were used to evaluate the hypotheses, controlling for potential confounders such as negative affect, body mass index, age, and sex. The study design allows for the investigation of causal relationships and the examination of trait positive affect as a potential protective factor against inflammation induced by sleep disruption.

The data analysis likely involves fitting linear mixed effects models to assess the moderating effect of trait positive affect on the associations between sleep condition and stimulated monocyte production of TNF and IL-6. Potential confounders such as negative affect, body mass index, age, and sex are controlled for in the analysis. Statistical tests, such as t-tests and linear regression, are used to determine the significance of the moderating effects. The study may also explore potential interactions between trait positive affect and other covariates in predicting cellular inflammation.

The results indicate that trait positive affect significantly moderates the associations between sleep disruption and stimulated monocyte production of IL-6 and its co-expression with TNF, such that inflammatory responses are blunted among those high in positive affect. This effect is independent of negative affect and other potential confounders. Although the effect on TNF is marginally significant, the findings suggest that high trait positive affect may protect individuals from the inflammatory activation following sleep disruption. The results are consistent with the hypothesis

including the relatively small sample size (n = 79) and the use of a homogeneous sample of good sleeping adults, which may limit the generalizability of the findings to other populations. Additionally, the study relies on self-reported measures of trait positive affect and negative affect, which may be subject to recall bias. Furthermore, the study does not investigate potential mechanisms underlying the moderating effect of positive affect on inflammation. Addressing these limitations, such as using a larger and more diverse sample and incorporating objective measures of affect, would

				and provide evidence for the role of positive affect in mitigating the adverse health consequences of sleep disturbance.	strengthen the validity and applicability of the findings.
Huscher et al. (2008)	The research question aims to identify patterns of self-reported health problems associated with the dose and duration of glucocorticoid intake in unselected patients with rheumatoid arthritis (RA) from routine practice. The question is clearly defined and relevant, addressing the need for real-life data on the adverse effects of glucocorticoids in a	The methodology involves analyzing data from 1066 patients with rheumatoid arthritis (RA) to investigate the association between glucocorticoid dose and duration and self-reported health problems. Clinical status and drug treatment were reported by physicians, while patients reported health problems over the past 6 months using a comprehensive list of symptoms. Patients were categorized based on	The data analysis involves comparing the frequency of self-reported health problems among different groups of rheumatoid arthritis (RA) patients based on glucocorticoid dosage and duration. Two distinct patterns of adverse events related to glucocorticoid dose were identified: a "linear" rising with increasing dose and a "threshold pattern" beyond a certain dose	The results demonstrate dose-related associations between glucocorticoid intake and self-reported health problems in patients with rheumatoid arthritis (RA). Specifically, the frequency of adverse events increased with glucocorticoid dosage, with two distinct patterns observed: a linear increase with increasing dose and a threshold effect at	The abstract does not explicitly discuss the limitations of the study. However, several potential limitations can be inferred from the methodology and results. These may include the reliance on self-reported health problems, which may be subject to recall bias or underreporting, and the lack of detailed information on potential confounding factors or

diverse patient population. By analyzing self-reported health problems in relation to glucocorticoid dose and duration, the study seeks to inform clinicians about the potential risks associated with glucocorticoid therapy and improve the benefit-risk ratio of treatment decisions. Overall, the research question is appropriate and aligns with the study objectives.

glucocorticoid dosage and duration, and comparisons were made with a group not receiving glucocorticoid treatment. The study utilizes a large sample size and real-world data from routine practice, enhancing the generalizability of the findings. However, the methodology could be strengthened by providing more details on the criteria used to categorize patients and the methods used to collect and analyze patient-reported health problems. Despite this limitation, the methodology is appropriate for addressing the research question and provides valuable insights into the adverse effects of glucocorticoid therapy in RA patients.

threshold. Specific health problems, such as cushingoid phenotype, ecchymosis, leg edema, and sleep disturbance, showed dose-related associations, while others, such as glaucoma and depression, exhibited threshold effects at higher doses. The analysis provides valuable insights into the dose-dependent nature of glucocorticoid-related adverse events and highlights potential threshold effects at higher doses. Overall, the data analysis is robust and supports the study's conclusions regarding the association between glucocorticoid dose and self-reported health problems in RA patients.

higher doses. Specific health problems showed dose-dependent associations, while others exhibited threshold effects beyond certain dose thresholds. These findings are consistent with biological mechanisms and clinical observations, highlighting the importance of optimizing glucocorticoid therapy to minimize adverse effects while maximizing therapeutic benefits. Overall, the results effectively address the research question and provide valuable insights into the dose-dependent nature of glucocorticoid-related adverse events in RA patients.

comorbidities that could influence the association between glucocorticoid dose and adverse events. Additionally, the study's retrospective design and reliance on routine practice data may limit the ability to establish causal relationships or generalize findings to other patient populations. Despite these limitations, the study provides valuable real-world data on the adverse effects of glucocorticoids in RA patients and offers insights that can inform clinical practice and treatment decisions. Addressing these limitations in future research would enhance the robustness and generalizability of the findings.

Inutsuka et al. (2016)	The research question is clearly defined and relevant, focusing on investigating the physiological importance of orexin neuronal activity in nociception and pain regulation. Specifically, the study aims to elucidate the role of orexin neurons in modulating pain-related behaviors and analgesia. The question addresses an important gap in understanding the neural mechanisms underlying nociceptive perception and pain regulation, particularly the involvement of orexin signaling. Overall, the research question aligns well with the objectives of the study and provides a clear direction for the investigation.	The methodology involves selective ablation of orexin neurons in adult-stage animals and pharmacogenetic activation of orexin neurons to assess their effects on pain-related behaviors. Additionally, fiber photometry recordings of orexin neurons in conscious animals are conducted to examine the correlation between orexin neuronal activity and nociception. These experimental approaches allow for the assessment of the physiological role of orexin neurons in nociceptive perception and pain regulation in a controlled laboratory setting. The methodology is well-described and suitable for investigating the	The data analysis includes the assessment of pain-related behaviors following selective ablation or activation of orexin neurons, as well as the analysis of fiber photometry recordings to determine the correlation between orexin neuronal activity and nociception. Statistical analyses are likely conducted to compare pain-related behaviors between experimental and control groups, and to evaluate the relationship between orexin neuronal activity and nociceptive stimuli. The analysis is appropriate for the experimental design and allows for the evaluation of the specific hypotheses	The results demonstrate that selective ablation of orexin neurons enhances pain-related behaviors, while pharmacogenetic activation of orexin neurons induces analgesia. Additionally, correlative activation of orexin neurons during nociception is observed using fiber photometry recordings in conscious animals. These findings provide evidence for an integrative role of orexin neurons in nociceptive perception and pain regulation. The results are presented clearly and are consistent with the objectives of the study, supporting the hypothesis regarding the involvement of orexin signaling in pain	While the study provides valuable insights into the role of orexin neurons in nociception and pain regulation, it has some limitations. The use of animal models may not fully replicate the complexity of human pain conditions, and translational relevance to clinical settings requires further investigation. Additionally, the specific mechanisms underlying the observed effects of orexin neuronal activity on pain-related behaviors and analgesia remain to be elucidated. Future studies should consider addressing these limitations and exploring the broader neural circuitry

		research question.	regarding the role of orexin neurons in nociception and pain regulation.	modulation.	involved in orexin-mediated pain modulation.
Irwin et al. (2004)	The research question is clear and well-defined: whether plasma levels of IL-6 and TNF are associated with disordered sleep in alcohol dependence.	The methodology seems robust, employing polysomnography and serial blood sampling across baseline, partial sleep deprivation, and recovery nights.	The data analysis appears thorough, adjusting for potential confounders such as alcohol consumption and body mass index.	The results indicate nocturnal elevations of IL-6 and TNF in alcoholics, as well as greater nocturnal increases of TNF following sleep deprivation.	Some limitations include the relatively small sample size and the focus on African American alcoholics, which may limit generalizability.
Jahangirvand et al. (2016)	The research question is clearly defined and relevant, focusing on investigating the involvement of orexin-1 (OX1) receptors in the nucleus accumbens (NAc) in modulating antinociception induced by intra-lateral hypothalamus (LH) administration of carbachol in the formalin test, an animal	The methodology involves the use of rats implanted with cannulae in the LH and NAc for microinjections of carbachol and SB334867, an OX1 receptor antagonist, respectively. The formalin test is employed as an animal model of persistent inflammatory pain. Different doses of SB334867 are	The data analysis involves the calculation of pain scores at 5-minute intervals during the formalin test following administration of SB334867 into the NAc. Results are presented as changes in pain scores over time and are analyzed to assess the dose-dependent effects of OX1 receptor	The results demonstrate that administration of SB334867 into the NAc decreases LH chemical stimulation-induced antinociception dose-dependently in both the early and second phases of the formalin test, indicating a role for OX1 receptors in the NAc in modulating inflammatory pain.	While the study provides valuable insights into the role of OX1 receptors in the NAc in modulating inflammatory pain, it has some limitations. The use of an animal model may not fully replicate the complexity of human pain conditions, and translational relevance to clinical settings

	model of persistent inflammatory pain. The study aims to elucidate the role of OX1 receptors in the NAc in pain modulation, which addresses an important aspect of orexin-mediated pain regulation. The research question aligns with the objectives of the study and provides a clear direction for the investigation.	administered into the NAc prior to intra-LH carbachol injection, and pain scores are calculated at 5-minute intervals for a 60-minute test period. The experimental design allows for the assessment of the dose-dependent effects of OX1 receptor blockade in the NAc on LH chemical stimulation-induced antinociception. Overall, the methodology is well-described and suitable for investigating the research question.	blockade on LH chemical stimulation-induced antinociception. The statistical analysis is appropriate for the experimental design and allows for the evaluation of the specific hypothesis regarding the involvement of OX1 receptors in the NAc in modulating inflammatory pain.	These findings support the hypothesis that OX1 receptors in the NAc may be involved in the modulation of inflammatory pain induced by intra-LH carbachol administration. The results are presented clearly and are consistent with the objectives of the study, providing evidence for the involvement of orexin signaling in pain regulation.	requires further investigation. Additionally, the study focuses on the specific role of OX1 receptors in the NAc, and other brain regions involved in pain modulation may also contribute to the observed effects. Future studies should consider addressing these limitations and exploring the broader neural circuitry underlying orexin-mediated pain regulation.
Jiang et al. (2022)	The research question aims to investigate the pharmacology and toxicology of prednisolone (PN) by analyzing its effects on the sleep phenotype	The methodology involves exposing zebrafish to prednisolone (PN) and analyzing its effects on sleep phenotype, melatonin secretion, and internal	The data analysis involves analyzing sleep phenotype, melatonin secretion, and gene expression patterns in zebrafish exposed to	The results demonstrate that exposure of zebrafish to prednisolone (PN) leads to decreased melatonin secretion, shortened sleep time,	The abstract does not explicitly discuss the limitations of the study. However, several potential limitations can be inferred from the

and internal circadian oscillation of zebrafish. Specifically, the study aims to elucidate the detailed mechanism by which PN disrupts sleep patterns and circadian rhythms in zebrafish. The question is well-defined, relevant, and addresses an important gap in understanding the mechanisms underlying the sleep-disrupting effects of glucocorticoids. By utilizing zebrafish as a model organism, the study offers insights into the potential mechanisms that may contribute to sleep disorders associated with long-term glucocorticoid use in humans. Overall, the research question is appropriate and aligns with the study objectives.

circadian rhythm. Sleep time and melatonin secretion were measured as indicators of sleep quality, while the expression of per and cry genes was assessed to evaluate the internal circadian oscillation. The study utilized appropriate techniques, including behavioral analysis, gene expression analysis, and luciferase reporter assays, to investigate the mechanisms underlying the effects of PN on sleep and circadian rhythms. However, the methodology lacks details on certain aspects, such as the dosage and duration of PN exposure and the sample size used in each experimental group. Additionally, the study did not explore potential confounding factors or alternative mechanisms that may contribute to

prednisolone (PN). Statistical methods were used to compare sleep parameters and gene expression levels between PN-exposed and control groups. Additionally, luciferase reporter assays were conducted to investigate the regulation of gene expression by PN via the glucocorticoid receptor (GR). The analysis includes appropriate statistical tests to determine significance levels and identify differences between experimental groups. The findings suggest that PN exposure leads to decreased melatonin secretion, shortened sleep time, and alterations in the expression of circadian rhythm-related genes. The data analysis is robust and supports

and alterations in the expression of circadian rhythm-related genes, including per and cry. Specifically, PN exposure upregulates the expression of per and cry genes, resulting in a delay in the phase of the zebrafish behavioral rhythm. Furthermore, luciferase reporter assays reveal that PN represses per2 and cry1aa expression via the glucocorticoid receptor (GR), leading to decreased melatonin secretion and subsequent sleep disorders. These findings highlight the mechanisms underlying the effects of glucocorticoids on sleep and provide valuable insights into the pharmacology and toxicology of PN. Overall, the results effectively address the research question and

methodology and results. These may include the lack of detailed information on PN dosage and duration of exposure, potential confounding factors not accounted for in the analysis, and the use of zebrafish as a model organism, which may not fully recapitulate the complexities of human sleep disorders. Additionally, the study focused on the effects of PN on sleep and circadian rhythms but did not investigate other potential adverse effects or alternative mechanisms of action. Despite these limitations, the study provides valuable insights into the pharmacological and toxicological effects of PN in zebrafish and lays the foundation for future research in this

		the observed effects. Despite these limitations, the methodology is appropriate for addressing the research question and provides valuable insights into the pharmacological and toxicological effects of PN in zebrafish.	the study's conclusions regarding the mechanisms underlying the effects of PN on sleep and circadian rhythms in zebrafish.	contribute to our understanding of the mechanisms by which PN disrupts sleep patterns and circadian rhythms in zebrafish.	area. Overall, acknowledging and addressing these limitations would enhance the interpretation and generalizability of the study findings.
Johnson et al. (2006)	The research question is well-defined and relevant, focusing on the relationship between HPA axis activity and rheumatic diseases, specifically Sjögren's syndrome (SS) and fibromyalgia (FM). The question is significant as it explores potential physiological mechanisms underlying these autoimmune conditions.	The methodology is appropriate for the research question. Using oCRH stimulation to assess ACTH and cortisol responses provides direct and indirect measures of pituitary and adrenal function. The inclusion of both SS and FM patients, along with a subgroup meeting criteria for both conditions, allows for comparative analysis.	The data analysis appears thorough, comparing basal ACTH and cortisol levels, as well as responses to oCRH stimulation between patient groups and controls. The use of peak, delta, and net integrated responses provides a detailed assessment of the HPA axis activity. Statistical significance is noted, enhancing the robustness of the findings.	The results indicate significant differences in HPA axis function between SS and FM patients, with SS patients showing hypoactivity and FM patients showing a somewhat exaggerated response. The intermediate response in the SS-FM subgroup provides additional insight. However, the precise physiological implications need further exploration.	Limitations include a potential small sample size, which might affect the generalizability of the findings. The study might also benefit from a longitudinal approach to assess changes over time. Additionally, the study does not explore potential confounding factors, such as medication use or coexisting conditions.
Julie et al. (2020)	The research question	The methodology	Thematic analysis is	Results are well-	Limitations include a

	is clearly defined: examining the impact of culture on employees' perception of safety. It aligns with the qualitative approach used in the study.	(qualitative approach, semi-structured interviews) is appropriate for exploring perceptions and experiences. Sampling and data collection methods are well-explained.	systematically applied to interview transcripts, ensuring rigor and depth in identifying themes related to safety perceptions and cultural influences.	supported by quotes and examples from participants, providing a rich understanding of how culture influences safety perceptions in the workplace.	small sample size from a specific industry, which may limit generalizability. Additionally, potential biases in participant responses could affect findings.
Kang et al. (2012)	The research question investigates the longitudinal risk of developing autoimmune diseases in patients with obstructive sleep apnea (OSA), addressing a significant gap in knowledge regarding the potential association between OSA and autoimmune disorders. The question is clearly defined and relevant, providing a basis for meaningful investigation.	The methodology involves a retrospective cohort study using data from the Taiwan Longitudinal Health Insurance Database. Patients with OSA were identified and matched with controls, and both cohorts were tracked over a five-year period to assess the development of autoimmune diseases. Cox proportional hazard regression was appropriately employed to compare the risk of autoimmune diseases between the study cohort and controls while adjusting for relevant factors. Overall, the	The data analysis effectively compares the incidence of autoimmune diseases between patients with OSA and matched controls over the five-year follow-up period. Stratified Cox proportional hazard regression is utilized to compute the hazard ratio, providing a clear measure of the relative risk associated with OSA. Adjustment for confounding factors enhances the validity of the findings. The statistical significance of the results is appropriately reported.	The results indicate that patients with OSA have a significantly higher risk of developing autoimmune diseases compared to controls, with the hazard ratio estimated at 1.91 after adjusting for relevant factors. This finding suggests a potential association between OSA and autoimmune disease risk. The reported incidence rates and confidence intervals support the reliability of the results.	While the study provides valuable insights into the association between OSA and autoimmune disease risk, several limitations should be considered. The retrospective design may introduce biases, and reliance on administrative databases may limit the accuracy of OSA and autoimmune disease diagnoses. Additionally, the study lacks detailed clinical information, such as OSA severity and autoimmune disease subtypes,

		methodology is robust and suitable for investigating the research question.			which could affect the interpretation of results. Addressing these limitations in future research could enhance the robustness and generalizability of the findings.
Kapsimalis et al. (2005)	The research question is clearly defined, aiming to explore the role of cytokines in regulating spontaneous non-rapid eye movement (NREM) sleep and the interactions between cytokines and neurohormonal systems in sleep-wake regulation. This addresses an important aspect of sleep physiology, considering the complex network of cytokines and their potential impact on sleep mechanisms in the brain. The question	The methodology likely involves a review and synthesis of existing literature on cytokines and sleep mechanisms. Specific details on the literature search strategy and inclusion criteria are not provided in the abstract. However, the review approach is appropriate for summarizing selected findings and advancing our understanding of the relationship between cytokines and sleep. The study likely	As this is a review article, data analysis primarily involves the synthesis and interpretation of findings from existing studies on cytokines and sleep. The article likely discusses the mechanisms through which cytokines, such as interleukin-1 and tumor necrosis factor, regulate NREM sleep and their interactions with neurohormonal systems. While no statistical analyses are conducted, the	The results highlight the increasing evidence of a role for cytokines, particularly interleukin-1 and tumor necrosis factor, in regulating spontaneous NREM sleep. The article discusses the sites of action of cytokines in the brain, including the hypothalamic preoptic area and basal forebrain, and their mechanisms of action on sleep, such as direct receptor-mediated effects and modulation of	The abstract does not explicitly discuss limitations; however, potential limitations of the study may include the reliance on existing literature, which may vary in quality and study design. Additionally, the review may be subject to publication bias, as only selected findings are highlighted. Furthermore, the complexity of cytokine interactions with sleep mechanisms and neurohormonal systems may present challenges in fully elucidating their roles

	is comprehensive, encompassing both the effects of cytokines on sleep and their interactions with neurohormonal systems, providing valuable insights into the underlying mechanisms of sleep regulation.	relies on observational and experimental studies investigating the effects of cytokines on sleep and their mechanisms of action.	review provides a comprehensive overview of the current understanding of cytokine-mediated effects on sleep.	neurotransmitter synthesis and release. Additionally, the article emphasizes the significant overlap between cytokines and neurohormonal systems in sleep-wake regulation.	in sleep regulation. Addressing these limitations would enhance the reliability and validity of the review.
Karatas et al. (2017)	The research question is clearly defined, aiming to evaluate the sleep quality (SQ) in rheumatoid arthritis (RA) patients treated with anti-tumor necrosis factor alpha (anti-TNF-α) therapy. This addresses an important aspect of RA management, considering the impact of disease	The methodology involves a prospective observational and open-label study of RA patients, with a total of 35 patients enrolled. Patients with high disease activity (DA) were initiated on anti-TNF-α therapy, while those in remission served as a comparison group. SQ was assessed	The data analysis likely involves comparing the total PSQI scores and the frequency of poor SQ between the high DA and remission groups, as well as assessing changes in PSQI scores before and after anti-TNF-α therapy initiation. Statistical tests, such as Mann-Whitney U test and paired t-test, may be used to	The results indicate that a majority of RA patients experience poor SQ, with higher PSQI scores observed in the high DA group compared to the remission group. Following anti-TNF-α therapy initiation, significant improvements in SQ, as assessed by the PSQI test, are observed in the high DA group. However,	The study may have several limitations, including the relatively small sample size and the lack of a control group receiving alternative treatments or no treatment. Additionally, the open-label design may introduce bias, and the short duration of follow-up after therapy initiation may not capture long-term effects on SQ. Furthermore, the

	activity and treatment on sleep disturbances, which can significantly affect quality of life and pain perception. The question is comprehensive, assessing both subjective (Pittsburgh Sleep Quality Index - PSQI) and objective (polysomnography - PSG) parameters of SQ in RA patients undergoing anti-TNF-α therapy.	using the PSQI questionnaire and PSG. The study design allows for the evaluation of changes in SQ before and after anti-TNF-α therapy initiation, providing valuable insights into the effectiveness of treatment on sleep disturbances in RA patients.	determine the significance of differences observed. Additionally, subgroup analyses may be conducted based on disease activity status and treatment response to further elucidate the relationship between RA severity, treatment, and SQ.	no significant differences are found by PSG. These findings suggest that anti-TNF-α therapy may improve subjective perceptions of sleep quality in RA patients, despite no significant changes observed in objective sleep parameters.	study does not investigate potential mechanisms underlying the observed improvements in SQ with anti-TNF-α therapy. Addressing these limitations would strengthen the validity and applicability of the findings.
Kasi et al. (2012)	The research question aims to investigate the severe complications associated with the use of rituximab, a monoclonal antibody used in the treatment of various hematological malignancies and autoimmune disorders.	The methodology involves a comprehensive review of evidence from randomized controlled trials, post-marketing surveillance data, and case reports to identify severe complications associated with	The data analysis involves synthesizing evidence from various sources, including clinical trials, post-marketing surveillance data, and case reports, to identify severe complications associated with	The results summarize the severe complications associated with rituximab use, leading to critical care admission, based on evidence from clinical trials, post-marketing surveillance data, and	The review acknowledges several limitations, including the absence of specific details on search strategies and inclusion criteria, which may affect reproducibility and transparency.

Specifically, the study focuses on adverse events necessitating critical care admission, highlighting the need for effective management and prevention strategies. By addressing this question, the review provides valuable insights into the safety profile of rituximab and its implications for critical care practice. Overall, the research question is relevant and well-defined, addressing an important aspect of rituximab therapy that requires attention in clinical practice.

rituximab use. While the specific search strategies and inclusion criteria are not detailed, the review appears to adopt a systematic approach to data collection and analysis. Additionally, the methodology discusses management and prevention strategies for common complications associated with rituximab therapy. However, the methodology lacks specific details on the selection and appraisal of included studies, potentially limiting the transparency and reproducibility of the review process. Nonetheless, the methodology is appropriate for addressing the research question and synthesizing evidence on rituximab-related severe complications.

rituximab use. The analysis highlights common adverse events leading to critical care admission, along with management and prevention strategies for these complications. While the analysis provides valuable insights into the safety profile of rituximab, it primarily relies on qualitative synthesis of evidence rather than quantitative measures or statistical analysis. Nonetheless, the data analysis effectively summarizes key findings and informs the discussion on rituximab-related severe complications requiring critical care intervention.

case reports. Key findings include an increasing number of serious adverse events associated with rituximab therapy, necessitating critical care admission, and complicating management. Additionally, the results discuss management and prevention strategies for common complications, providing insights into the clinical implications of rituximab therapy in the critical care setting. Overall, the results provide a comprehensive overview of rituximab-related severe complications and their implications for critical care practice.

Additionally, while the review synthesizes evidence from diverse sources, the absence of quantitative measures or statistical analysis limits the depth of data analysis. Furthermore, the review primarily focuses on summarizing existing evidence rather than presenting new findings or conducting original research. Despite these limitations, the review provides valuable insights into rituximab-related severe complications requiring critical care intervention, serving as a useful resource for clinicians and researchers. Future studies could address the identified limitations and further explore specific aspects of rituximab

					therapy in critical care practice.
Kasitanon et al. (2013)	The research question is well-defined: to determine the prevalence of sleep disturbance in systemic lupus erythematosus (SLE) patients, identify associated factors, and assess the correlation between changes in clinical parameters and sleep quality over time. The question is relevant and addresses an important aspect of SLE management and quality of life.	The methodology involves a prospective observational study with repeated assessments over three months. Data collection includes demographic information, clinical parameters, standardized assessment tools (e.g., Pittsburgh Sleep Quality Index, disease activity index, quality of life measures), and psychiatric evaluations. The study design allows for the examination of longitudinal changes in sleep quality and associated factors. However, the study's generalizability may be limited due to its focus on female SLE patients in	The data analysis appears appropriate for the study objectives, utilizing descriptive statistics, logistic regression analyses, and correlation assessments. Statistical tests are used to identify associations between sleep disturbances and various factors, such as depression and clinical parameters. However, the statistical methods used and adjustments for potential confounders are not explicitly mentioned, which may affect the robustness of the findings. More details on the statistical analysis plan would	The results are presented clearly, providing information on the prevalence of sleep disturbances in SLE patients, associated factors, and correlations between changes in clinical parameters and sleep quality over time. The inclusion of descriptive statistics and regression analyses aids in understanding the relationships observed. However, further details on the magnitude of associations and the clinical significance of findings would provide a more comprehensive interpretation.	The study acknowledges several limitations, including its observational nature, which precludes establishing causality between variables. The relatively small sample size and focus on female SLE patients in Thailand may limit the generalizability of findings to other populations. Additionally, the study relies on self-reported measures, which may be subject to recall and reporting biases. Further discussion on potential confounders, such as medication usage or comorbidities, would

		Thailand and the relatively small sample size. Further details on participant recruitment, inclusion criteria, and potential biases would enhance transparency.	enhance the rigor of the study.		strengthen the interpretation of results.
Kaur et al. (2018)	The research question is well-defined and relevant, aiming to explore the properties, possible mechanisms, and functions of melatonin in chronic pain, particularly in the context of circadian rhythms and chronotherapy. This addresses an important aspect of pain management, considering the potential role of melatonin in modulating pain perception and providing neuroprotection. The	The methodology appears to involve a literature review approach to synthesize existing evidence on the properties and mechanisms of melatonin in chronic pain. While specific details on the literature search strategy and inclusion/exclusion criteria are not provided, the review approach is appropriate for exploring the multifaceted role of melatonin in pain modulation and	As this is a review article, data analysis is primarily qualitative, involving the synthesis and interpretation of findings from existing studies on melatonin and chronic pain. The reported information encompasses the properties of melatonin, its effects on circadian rhythms, and its potential role in pain modulation and neuroprotection. While no statistical analyses are conducted, the	The results of the review highlight the multifaceted role of melatonin in chronic pain, emphasizing its involvement in circadian rhythms, pain modulation, and neuroprotection. Specifically, melatonin is described as having analgesic and neuroprotective effects, with potential implications for chronotherapy in chronic pain management. The findings provide valuable insights into the mechanisms	The study acknowledges several limitations, including the reliance on existing literature, which may vary in quality and study design. Additionally, the lack of specific details on the literature search strategy and inclusion/exclusion criteria introduces potential bias and affects the reproducibility of the findings. Furthermore, while the review provides a comprehensive overview of the role of melatonin in chronic pain, the specific

	question is comprehensive, covering both basic science aspects and potential clinical applications of melatonin in chronic pain conditions.	chronotherapy. However, without transparency regarding the literature selection process, the rigor of the methodology is somewhat limited.	review provides a comprehensive overview of the mechanisms underlying the analgesic and neuroprotective effects of melatonin in chronic pain conditions.	through which melatonin may exert its beneficial effects in chronic pain conditions, offering potential avenues for therapeutic intervention.	mechanisms and clinical implications may require further validation through experimental and clinical studies.
Kazt et al. (2023)	The research question addresses an important gap in the literature concerning the prevalence of sleep disorders (OSA risk, RLS symptoms, SS) in RA patients and their associated factors. It explores the relationship between RA disease activity, medication use, and sleep quality outcomes. However, while the prevalence and associations are examined, the study	Strengths: The study utilizes a large cohort from a national database, which enhances generalizability. Validated screening measures for OSA risk and RLS symptoms are employed, and sleep quality is assessed using a recognized index. Multivariable logistic regression models are used to identify predictors, which is appropriate for analyzing associations. Weaknesses: The	Strengths: Multivariable logistic regression models are applied to identify independent predictors of sleep disorders and quality. This statistical approach allows for controlling potential confounders and assessing associations. Weaknesses: The analysis does not include more advanced statistical techniques or sensitivity analyses to further explore the robustness of the	Strengths: The study reports clear prevalence rates of OSA risk, RLS symptoms, and SS among RA patients. Significant associations with RA disease activity and specific medications (e.g., abatacept) are highlighted, providing valuable clinical insights. The association between RLS, SS, and perceived sleep quality adds to understanding patient-	The study acknowledges several limitations, including the reliance on self-report measures for sleep assessment, potential selection biases inherent in database studies, and the cross-sectional nature limiting causal inference. It also notes the need for further research to explore causal pathways and long-term health consequences. However, additional

	does not delve into causal pathways or long-term consequences extensively.	methodology relies heavily on self-report measures for sleep duration and quality, which can introduce reporting biases. Additionally, the study lacks objective sleep measures (e.g., polysomnography) and longitudinal data, limiting the ability to establish causal relationships.	findings. Moreover, the absence of mediation or moderation analyses limits deeper insights into the relationships examined.	reported outcomes. Weaknesses: The results focus primarily on associations rather than causation, and the implications of these findings for clinical practice or interventions are not fully explored. There's also a lack of discussion on potential interactions between different sleep disorders in RA patients.	limitations could include the lack of diversity in the cohort or the potential for unmeasured confounding factors influencing the associations found.
Khalyfa et al. (2011)	The study aims to assess sleepiness, TNF-α plasma levels, and TNF-α gene variance in pediatric OSA.	The study design involves a case-control approach, with children being evaluated for OSA and matched control children assessed for sleepiness and TNF-α plasma levels. The inclusion of a modified Epworth Sleepiness Scale questionnaire and blood draw the morning after nocturnal	Comprehensive analysis reveals increased TNF-α levels and sleepiness in OSA, with genetic variance playing a role, particularly the TNF-α -308G SNP.	The results indicate that morning TNF-α levels and Epworth Sleepiness Scale scores are increased in the presence of OSA, with substantial variability observed. TNF-α plasma concentrations are globally increased in OSA, but most of the variance is attributable to the presence or	One limitation of the study is the observational design, which precludes causal inference and may be subject to confounding factors. Additionally, the study's reliance on a single assessment of TNF-α levels and sleepiness symptoms may not capture

		polysomnography enhances the depth of data collection. Genotyping and allelic frequencies determination for TNF-α single nucleotide polymorphisms (SNPs) add genomic information to the analysis. However, the study design does not allow for causal inference due to its observational nature.		absence of the TNF-α -308G gene polymorphism. These findings suggest a potential genetic influence on TNF-α levels and sleepiness symptoms in pediatric OSA, particularly related to the TNF-α -308G SNP.	longitudinal changes or variability over time. Further research with larger sample sizes and longitudinal designs is needed to validate the findings and elucidate the underlying mechanisms linking TNF-α gene polymorphisms and sleepiness in pediatric OSA.
Khan et al. (2020)	The research question addresses a significant gap in care for patients with systemic lupus erythematosus (SLE) by investigating the impact of a digital therapeutic intervention on quality of life. It aims to determine whether a mobile app for self-tracking dietary, environmental, and lifestyle triggers, paired with telehealth	The study employed a randomized controlled pilot design, assigning adults with SLE to either the digital therapeutic intervention plus usual care or usual care alone for 16 weeks. Primary outcome measures included changes in health-related quality of life (HRQoL) using validated tools. The methodology appears robust, utilizing appropriate measures	The data analysis involved comparing changes in HRQoL measures between the intervention and control groups using validated tools such as the Functional Assessment of Chronic Illness Therapy-Fatigue (FACIT-F) and Lupus Quality of Life (LupusQoL). Statistical analysis was conducted to determine significant improvements in	The results indicate that the digital therapeutic intervention resulted in statistically significant and clinically meaningful improvements in HRQoL compared to usual care alone. The intervention group showed greater improvement in multiple domains of HRQoL, including fatigue, pain	While the study provides valuable insights into the potential benefits of the digital therapeutic intervention for patients with SLE, several limitations should be considered. These include the relatively small sample size, which may limit the generalizability of the findings, and the short duration of the study period.

	coaching, can improve quality of life in patients with SLE compared to usual care alone. The question is relevant, clearly stated, and addresses an unmet need in SLE management.	and study duration to assess the impact of the intervention on HRQoL in patients with SLE.	HRQoL domains. The statistical methods used appear appropriate and rigorous, providing reliable insights into the impact of the digital therapeutic intervention on HRQoL outcomes in patients with SLE.	interference, emotional health, and physical health. The findings are clearly presented, supported by statistical analysis, and demonstrate the efficacy of the intervention in enhancing HRQoL outcomes in patients with SLE.	Additionally, the study may be subject to selection bias, as participants were recruited voluntarily, and there may be challenges in sustaining long-term adherence to the digital intervention beyond the study period. Further research with larger sample sizes and longer follow-up periods is needed to confirm and extend these findings.
Kim et al. (2021)	The research question is clearly defined, addressing the potential of metformin as a treatment for Sjögren's syndrome.	The methodology appears sound, focusing on metformin's anti-inflammatory and immunomodulatory mechanisms relevant to SS.	The data analysis seems appropriate, involving comparisons of salivary gland function before and after treatment.	The results demonstrate the potential of metformin to improve salivary gland function in Sjögren's syndrome.	Limitations may include lack of clinical trials or limited sample size, requiring further research for conclusive evidence.

Koh et al. (2022)	The research question is well-defined: to determine the effect of obstructive sleep apnea on metabolic function and the mechanisms underlying the increased risk of type 2 diabetes.	The methodology is robust, employing a two-stage hyperinsulinemic-euglycemic clamp procedure, stable isotopically labeled tracer infusions, and positron emission tomography, ensuring comprehensive evaluation of insulin action and secretion.	The data analysis appears thorough, effectively quantifying insulin resistance in various tissues and comparing glucose-stimulated insulin secretion between groups.	The results are well-supported by the data, demonstrating a significant association between OSA and insulin resistance in adipose tissue and skeletal muscles, highlighting the importance of considering OSA in evaluating metabolic function in obesity.	Although the study provides valuable insights, it may have limitations such as a relatively small sample size and potential confounding factors not fully addressed, which could affect the generalizability of the findings.
Kok et al. (2016)	The research question addresses whether chronic insomnia requiring sleep-inducing pills is associated with the development of autoimmune diseases (AID), particularly primary Sjögren's syndrome (pSS). The question is clear, relevant, and important for understanding the potential health implications of chronic insomnia. By investigating the	The methodology involves a population-based, nationwide longitudinal study using a claims dataset from the National Health Insurance Research Database. The study assembled a chronic insomnia group and a propensity score-matched comparison group to compare the incidence of AID between the two groups. The methodology includes appropriate	The data analysis includes descriptive statistics, hazard ratio calculations using Cox proportional hazards models, and sensitivity analysis to assess the robustness of the findings. The study reports incidence rates, adjusted hazard ratios, and confidence intervals to quantify the magnitude of risk for AID in individuals with chronic insomnia. Additionally, sensitivity analysis	The results indicate a significant association between chronic insomnia requiring sleep-inducing pills and an increased risk of future AID, with a 70% higher adjusted hazard ratio compared to the propensity score-matched comparison group. Additionally, a positive association with primary Sjögren's syndrome is observed. Sensitivity analysis confirms the robustness of the findings,	The study acknowledges several limitations, including those inherent to claims data, such as potential misclassification and incomplete information. While propensity score matching helps control for confounders, residual confounding cannot be entirely ruled out. Additionally, the study does not explore potential mechanisms

association between chronic insomnia and AID risk, the study aims to fill a knowledge gap in the literature and provide valuable insights into the relationship between sleep disorders and autoimmune conditions. Overall, the research question is well-defined and aligns with the study's objectives.	measures to control for potential confounders and bias, such as propensity score matching and sensitivity analysis. Additionally, the study reports person-time data, adjusted hazard ratios, and AID-free survival functions. While the methodology appears robust, limitations associated with claims data, such as potential misclassification and incomplete information, should be considered. Overall, the methodology is suitable for addressing the research question and provides valuable insights into the association between chronic insomnia and AID risk.	examines the time lag effect to further validate the results. The use of the Benjamini-Hochberg procedure controls the error rate. Overall, the data analysis is thorough and provides comprehensive insights into the association between chronic insomnia and AID risk, particularly primary Sjögren's syndrome.	showing an even stronger risk of AID after 5 years of follow-up. The results are presented clearly and supported by appropriate statistical measures and confidence intervals. Overall, the results provide compelling evidence of the association between chronic insomnia and AID risk, highlighting the potential health implications of sleep disorders.	underlying the association between chronic insomnia and AID risk. Furthermore, the study focuses on chronic insomnia requiring sleep-inducing pills, which may not capture all individuals with insomnia. Despite these limitations, the study provides valuable insights into the association between chronic insomnia and AID risk, but further research is needed to address remaining questions and potential mechanisms. Overall, the limitations are acknowledged, but additional research is warranted to validate and expand upon the findings.

Krajewska-Włodarczyk et al. (2018)	The research question aims to assess the occurrence of sleep disorders in patients with psoriatic arthritis (PsA) and psoriasis (Ps) and explore the relationship between sleep quality, disease severity, treatment, and patient-reported outcomes such as fatigue and quality of life. It is clear, focused, and relevant to understanding the impact of PsA and Ps on sleep quality and associated factors. By addressing this question, the study provides valuable insights into the prevalence of sleep disorders in these patient populations and their implications for overall well-being.	The methodology involves recruiting 62 patients with PsA, 52 patients with Ps, and a control group. Sleep quality is assessed using the Pittsburgh Sleep Quality Index (PSQI), while fatigue and quality of life are evaluated using the fatigue subscale of the FACIT-F questionnaire and the Health Assessment Questionnaire (HAQ), respectively. Disease severity is measured using the Psoriasis Area Severity Index (PASI) and the disease activity score of 28 joints (DAS 28). Pain severity is assessed using the Visual Analogue Scale (VAS). The methodology is comprehensive and utilizes validated tools to assess various aspects of	The data analysis includes comparisons of sleep quality between patients with PsA, patients with Ps, and the control group using the PSQI. Additionally, correlations and linear regression analyses are performed to explore the relationship between sleep quality and various clinical parameters, including pain, disease severity, treatment, and patient demographics. The analysis is thorough and provides valuable insights into the factors influencing sleep quality in PsA and Ps patients. However, more detailed reporting of statistical methods and effect sizes would enhance the	The results indicate that poor sleep quality is prevalent in both PsA and Ps patients, with higher rates compared to the control group. Sleep disorders are associated with worse quality of life and intense fatigue in both patient populations. Methotrexate treatment is not associated with sleeping disorders, but improvement in sleep quality is observed in patients treated with anti-TNF-α antibodies. Linear regression analyses identify several factors contributing to sleep quality, including pain, disease severity, patient age, and duration of psoriasis. These findings	The abstract acknowledges several limitations, including the relatively small sample size and potential confounding factors that may affect the interpretation of results. The cross-sectional design of the study precludes the establishment of causal relationships between sleep quality and disease parameters. Additionally, the study relies on self-reported measures of sleep quality, fatigue, and quality of life, which may be subject to recall or reporting bias. Furthermore, the study does not provide details on potential comorbidities or

	Overall, the research question is appropriate and aligned with the study objectives.	sleep and disease severity. However, the study could benefit from a larger sample size and additional details on patient recruitment and selection criteria. Overall, the methodology is suitable for addressing the research question.	transparency of the analysis. Overall, the data analysis is appropriate for addressing the research question and objectives of the study.	highlight the multifactorial nature of sleep disorders in PsA and Ps patients. Overall, the results effectively address the research question and provide valuable insights into the factors influencing sleep quality in these populations.	medication use that could impact sleep quality. Despite these limitations, the study provides valuable insights into the prevalence and factors associated with sleep disorders in PsA and Ps patients. Overall, the limitations are appropriately acknowledged and discussed in the context of the study findings.
Kronholm et al. (1996)	The research question is clearly defined: to investigate differences in sleep behavior between monozygotic twins discordant for BMI.	The methodology involves using a static charge-sensitive bed to record motor breathing and cardiac activity, coupled with questionnaires on sleep habits. This approach enhances data collection.	The data analysis appears thorough, correlating intrapair differences in sleep characteristics with physiological and anthropometric differences.	The results effectively demonstrate that obese twins exhibit disrupted sleep patterns compared to nonobese co-twins, with associations to obesity-related factors.	While the study identifies associations, it does not establish causality. Additionally, the reliance on self-reported sleep habits via questionnaire may introduce bias.

Kushikata et al. (1998)	The research question is clearly defined and relevant, aiming to investigate the effects of interleukin-4 (IL-4), an anti-inflammatory cytokine, on spontaneous sleep in rabbits. This addresses an important aspect of sleep regulation, particularly the potential involvement of cytokines such as IL-4 in modulating sleep-wake cycles. The question is comprehensive, considering the specific effects of IL-4 administration during different phases of the sleep-wake cycle and providing valuable insights into the cytokine network's	The methodology involves intracerebroventricular injections of IL-4 at different doses during the rest (light) period and active (dark) cycle in rabbits, followed by monitoring of spontaneous sleep. Time-matched control injections of saline are administered for comparison. The study design allows for the assessment of IL-4's effects on both non-rapid eye movement (NREM) sleep and rapid eye movement (REM) sleep. Overall, the methodology is appropriate for investigating the hypothesis regarding IL-4's influence on sleep regulation.	The data analysis includes the comparison of IL-4 injections at different doses and during different phases of the sleep-wake cycle with control injections of saline. Statistical analysis is likely performed to determine significant differences in spontaneous NREM sleep and REM sleep following IL-4 administration. The findings indicate that the three highest doses of IL-4 significantly inhibit spontaneous NREM sleep when administered during the light cycle, while the highest dose also decreases REM sleep. The analysis provides clear insights into IL-4's	The results demonstrate that IL-4 administration, particularly at higher doses during the rest (light) period, significantly inhibits spontaneous NREM sleep in rabbits. Additionally, the highest dose of IL-4 also decreases REM sleep. However, IL-4 administered during the active (dark) cycle has no effect on sleep. These findings provide compelling evidence supporting the hypothesis that IL-4, an anti-inflammatory cytokine, plays a role in regulating physiological sleep, further highlighting the involvement of a brain cytokine network in sleep regulation.	The study acknowledges several limitations, such as the focus on rabbit models, which may not fully represent human sleep physiology. Additionally, the use of intracerebroventricular injections of IL-4 may not precisely mimic physiological IL-4 release in the brain. Furthermore, the study design does not account for potential interactions between IL-4 and other cytokines or neurotransmitters involved in sleep regulation. Addressing these limitations, along with potential confounding factors, would enhance the validity and generalizability of the findings.

	role in regulating physiological sleep.		sleep-modulating effects.		
Lal et al. (2021)	The research question aims to investigate the pathophysiology and clinical management of excessive daytime sleepiness (EDS) in patients with obstructive sleep apnea (OSA), particularly focusing on residual EDS despite optimized therapy. This question is highly relevant given the clinical challenge of managing EDS in OSA patients and the potential impact on daily functioning and quality of life.	The methodology involves a comprehensive review of existing literature on the mechanisms underlying EDS in OSA patients, including experimental studies on chronic intermittent hypoxia, sleep fragmentation, neuroimaging findings, and clinical management strategies. While the review methodology is appropriate for synthesizing existing evidence, it does not involve primary data collection or experimentation.	As the study is a review article, data analysis primarily involves synthesizing and summarizing existing evidence from various studies, including experimental and clinical findings. The analysis appears thorough, with key findings and concepts effectively summarized and presented. However, the review does not involve original data analysis or statistical interpretation.	The results section of the review article presents key findings from the literature on the pathophysiology of EDS in OSA patients, including mechanisms involving chronic intermittent hypoxia, sleep fragmentation, neuroimaging findings, and clinical management strategies. The presentation of results is clear and organized, facilitating understanding for readers. However, since it is a review article, it does not	One limitation of the study is its reliance on existing literature, which may introduce biases associated with the selection and interpretation of studies included in the review. Additionally, the review may not capture the most recent advancements in the field, as it may be limited by the publication date of included studies. Furthermore, the generalizability of findings may be limited by variations

				present new empirical results.	in study populations and methodologies across included studies.
Lana et al. (2019)	The research question addresses the association between habitual meat consumption and changes in sleep duration and quality in older adults, an area with limited understanding. The study design allows for the examination of meat consumption as a potential predictor of sleep outcomes, filling a gap in current knowledge.	The study utilizes data from the Seniors-ENRICA cohort, comprising 1,341 participants aged ≥60 years, providing a robust sample size for analysis. Habitual meat consumption was assessed using a validated diet history, enhancing the reliability of dietary data. Sleep duration and quality were measured at baseline and follow-up, allowing for longitudinal analysis of sleep patterns. Logistic regression models were employed, adjusting for socio-demographic variables, lifestyle factors, morbidity, and baseline sleep indicators, strengthening the validity of the	Logistic regression analysis was performed to assess the association between meat consumption and changes in sleep duration and quality, controlling for potential confounders. The study reports a significant association between higher meat consumption and increased incidence of large sleep duration changes, snoring, and poor general sleep quality. The dose-response relationship observed, with each 100 g/d increment in meat intake associated with a 60% higher risk of adverse sleep outcomes, strengthens the evidence for a potential causal	The results indicate that higher habitual meat consumption, particularly ≥128 g/d, is associated with adverse changes in sleep duration and quality among older adults. Specifically, individuals in the highest tertile of meat consumption show an increased incidence of large decreases in sleep duration, snoring, and poor general sleep quality compared to those with lower meat intake. The consistency of results across different types of meat (red, processed, and white) suggests a broad effect of meat consumption on sleep outcomes.	This study's reliance on self-reported dietary intake and sleep data may introduce biases. The observational design precludes establishing causality, and residual confounding could affect results. Limited generalizability to younger age groups or other demographics and lack of information on potential mediators or moderators are also notable limitations. Additionally, the relatively short follow-up duration may not capture long-term effects.

		associations observed. However, the study could benefit from additional objective measures of sleep, such as polysomnography, to complement self-reported sleep data.	relationship between meat consumption and sleep disturbances. The analysis also considers subgroups, such as individuals with physical impairment, providing valuable insights into differential effects across demographic and health status groups.		
Lee et al. (2013)	The study provides a comprehensive review of the relationship between obesity, inflammation, and dietary factors, addressing the multifactorial nature of obesity and its impact on health. The research question aims to elucidate the role of diet in modulating inflammation associated with obesity, highlighting the importance of dietary interventions in	The methodology involves a thorough examination of existing literature, encompassing both observational and interventional studies to evaluate the effects of dietary factors on inflammation related to obesity. While the review lacks a specific study design, the inclusion of diverse sources and data synthesis techniques enhances the comprehensiveness and validity of the findings. However, a more	Data analysis primarily involves synthesizing findings from previous studies, including cross-sectional, observational, and interventional research. The review employs a narrative approach to data analysis, summarizing key findings and identifying patterns across different dietary factors and inflammatory markers. While the narrative synthesis offers	The results provide a comprehensive overview of the impact of dietary factors on inflammation related to obesity, highlighting the role of carbohydrates, dietary fat, vegetables, fruits, vitamins, minerals, phytoestrogens, probiotics, and prebiotics in modulating inflammatory markers. The review synthesizes evidence from multiple studies to elucidate the	Limitations of the review include the reliance on existing literature, which may introduce publication bias and limit the generalizability of findings. Additionally, the review primarily focuses on observational and interventional studies, which may have inherent limitations such as confounding variables and sample bias. The lack of a systematic review

	managing inflammatory conditions. The review synthesizes previous findings and identifies key areas for future research, contributing to our understanding of the complex interplay between diet, obesity, and inflammation.	structured approach to data selection and analysis could strengthen the methodological rigor of the review.	valuable insights into the relationship between diet and inflammation, a more systematic analysis of study outcomes and effect sizes could enhance the rigor and interpretability of the findings.	complex relationship between diet and inflammation, offering valuable insights into potential dietary interventions for managing inflammatory conditions associated with obesity.	methodology and standardized data extraction procedures could also affect the reliability and reproducibility of the findings. However, the review acknowledges these limitations and underscores the need for further research to validate the observed associations and elucidate underlying mechanisms.
Lehrskov et al. (2018)	The research question is clearly defined, aiming to investigate whether postprandial fatigue is regulated by interleukin-1 (IL-1) and whether IL-1 antagonism reduces postprandial fatigue in both lean and obese subjects. This addresses an important aspect of fatigue pathogenesis and its potential modulation by anti-inflammatory	The methodology involves a double-blind, crossover study in which lean and obese male subjects are randomized to receive either saline (placebo) or the IL-1 receptor antagonist anakinra. Postprandial fatigue is induced by having subjects run 30 minutes prior to a	The data analysis likely involves comparing postprandial fatigue scores and systemic concentrations of cytokines between the placebo and anakinra groups, as well as between lean and obese subjects. Statistical tests may be used to determine the significance of differences observed. The analysis would focus on assessing the	The results indicate that IL-1 antagonism led to a reduction in postprandial fatigue, with a more pronounced effect observed in obese individuals compared to lean individuals. This finding supports the hypothesis that IL-1 is involved in regulating postprandial fatigue,	The abstract does not explicitly discuss limitations; however, potential limitations of the study may include the small sample size and the focus on male subjects, which may limit the generalizability of the findings. Additionally, the use of anakinra as the IL-1 receptor antagonist may not fully reflect the physiological effects of

	treatment, particularly in the context of chronic diseases and obesity. The question is comprehensive, considering the impact of IL-1 on postprandial fatigue and its potential relevance in both lean and obese individuals.	high-fat, high-carbohydrate meal. Fatigue is assessed using the Stanford Sleepiness Scale, and blood samples are collected at baseline and after the intervention. Overall, the methodology is appropriate for investigating the effects of IL-1 antagonism on postprandial fatigue in both lean and obese subjects.	effect of IL-1 antagonism on reducing postprandial fatigue and whether this effect differs between lean and obese individuals. While specific details are not provided, the analysis is likely robust given the study design.	particularly under physiological conditions in both lean and obese individuals. The conclusion is clear and supported by the data obtained from the study.	endogenous IL-1. Furthermore, the study design does not account for potential confounding factors that may influence postprandial fatigue. Addressing these limitations would enhance the validity and applicability of the findings.
Lin et al. (2012)	The research question is well-defined and addresses an important gap in the literature regarding the association between smoking and obstructive sleep apnea (OSA). By exploring potential mechanisms linking smoking to	The use of PubMed and Medline databases is appropriate for identifying relevant studies on the topic.	The data analysis involves a qualitative synthesis of findings from selected studies rather than quantitative meta-analysis. While this approach provides a comprehensive overview of the evidence, it may limit the ability to quantify	The results summarize key findings from multiple studies, confirming the association between smoking and OSA. The discussion of potential mechanisms linking smoking to OSA pathophysiology provides valuable	The study acknowledges limitations such as potential publication bias and the exclusion of studies considering smoking as a confounding factor. However, additional limitations, such as the lack of detail on the

	OSA, the question is comprehensive and relevant to clinical practice.		effect sizes and assess heterogeneity across studies. Nonetheless, the discussion of potential mechanisms linking smoking to OSA pathophysiology adds depth to the analysis.	insights into the underlying biological processes. However, the conclusions drawn from these findings should be interpreted cautiously due to the complexity of OSA and smoking interactions.	search strategy and potential biases in study selection, could have been addressed. Overall, while the limitations are acknowledged, they may impact the comprehensiveness and generalizability of the findings.
Lin et al. (2020)	The research question is clear and relevant, focusing on the pathomechanism of RA, diagnosis, and the efficacy, benefits, and side effects of DMARDs. It addresses a significant clinical issue.	The methodology involves a comprehensive review of existing literature on DMARDs. It categorizes DMARDs into conventional synthetic, targeted synthetic, and biologic, examining their modes of action and clinical benefits. However, specifics on study selection criteria or search strategy are not provided.	The data analysis appears to be qualitative, synthesizing findings from various studies. This approach is suitable for a review article but lacks quantitative analysis or meta-analytical techniques that could provide more robust conclusions.	The results highlight significant advancements in DMARDs that have transformed RA from a debilitating disease to a manageable condition. They provide detailed information on the types of DMARDs, their mechanisms, and their clinical efficacy. However, exact figures or statistical analysis of the outcomes are not provided.	The review identifies key limitations, including side effects and high costs associated with DMARDs. However, it does not discuss the variability in patient responses or long-term outcomes comprehensively. The absence of primary data limits the ability to draw definitive conclusions.
Liu et al. (2022)	The study aims to investigate the circadian expression	The study likely employs a combination of human and animal research	Data analysis likely involves comparing the circadian expression	The study findings reveal a distinct circadian expression	Despite the promising findings, several limitations should be

	profile of clock genes in primary Sjögren's syndrome (pSS) and evaluate the therapeutic potential of melatonin in alleviating pSS symptoms. The research question addresses gaps in understanding the role of circadian clocks and potential therapeutic interventions in autoimmune diseases like pSS.	methods, including gene expression analysis in salivary glands from pSS patients and animal models, as well as melatonin administration to assess its therapeutic effects. This approach allows for a comprehensive investigation of both clinical and mechanistic aspects of pSS pathogenesis and treatment. However, specific details regarding sample sizes, experimental protocols, and animal models used would provide clarity and enhance the methodological rigor.	profiles of clock genes in salivary glands from pSS patients and animal models, possibly using techniques such as quantitative polymerase chain reaction (qPCR) or gene expression microarrays. Additionally, the effects of melatonin administration on salivary gland function, inflammation, and clock gene expression may be assessed through histological analysis, cytokine measurements, and gene expression assays. Statistical methods are employed to determine significance levels and correlations between variables.	profile of clock genes in the salivary glands of pSS patients and animal models, suggesting a potential role of circadian dysregulation in pSS pathogenesis. Furthermore, melatonin administration demonstrates improvements in salivary gland function, inhibition of inflammation, and regulation of clock gene expression in the pSS animal model, indicating a therapeutic potential for melatonin in pSS treatment. These results contribute valuable insights into the pathogenesis and management of pSS.	considered. The study may face challenges related to translating results from animal models to human patients, as well as potential differences in the underlying mechanisms of pSS between species. Additionally, the specific mechanisms by which melatonin exerts its therapeutic effects in pSS require further elucidation, and long-term studies are needed to assess the sustained efficacy and safety of melatonin treatment in pSS patients. Addressing these limitations would enhance the clinical relevance and applicability of the study findings.
MacDonald et al. (2020)	The research question is pertinent and addresses the role of	The review methodology involves a comprehensive summary	Data analysis in the review involves synthesizing findings	The results highlight the complex role of melatonin in RA. Some	The primary limitation is the contradictory nature of existing

	melatonin in the treatment of rheumatoid arthritis (RA), a significant inflammatory joint disorder. It seeks to understand the dual nature of melatonin's effects—both proinflammatory and anti-inflammatory—and its potential as an adjuvant therapy. This is crucial given the limitations of current RA treatments.	of existing research on the immunopathogenic characteristics of melatonin in RA, including both animal models and clinical trials. It examines various studies to provide a balanced perspective on the effects of melatonin. However, being a review, it does not involve primary data collection or experimental work, relying on the robustness and quality of included studies.	from multiple studies to draw conclusions about melatonin's role in RA. This includes comparing proinflammatory and anti-inflammatory effects, as well as examining the impact on circadian rhythms and clock gene expression in RA joints. The analysis is qualitative and integrative, aimed at providing a holistic understanding of the topic.	studies suggest that melatonin enhances proinflammatory activities, potentially exacerbating RA symptoms, while others indicate significant anti-inflammatory and immunoregulatory properties. The review also points out the relationship between circadian rhythm disturbances and RA, noting melatonin's influence on clock gene expression.	research, making it difficult to draw definitive conclusions about melatonin's role in RA. The review also does not include new experimental data, and the reliance on existing studies may incorporate their inherent biases and limitations. Furthermore, the diverse methodologies and populations of the included studies may contribute to inconsistent findings. Further research is needed to clarify melatonin's effects and optimize its use in RA treatment.
Magro et al. (2018)	The research question is well-defined: to characterize Systemic Lupus Erythematosus (SLE) patients in Malta, estimate the prevalence and incidence of SLE, characterize clinical	The methodology involves a cross-sectional study of 107 SLE patients identified based on SLICC classification criteria. Patients underwent interviews, blood and urine tests,	The data analysis appears appropriate for the study objectives, utilizing descriptive statistics to estimate prevalence and incidence of SLE, and correlation analysis to	The results are presented clearly, providing estimates of SLE prevalence and incidence in Malta, as well as demographic and clinical characteristics of SLE	The study acknowledges several limitations, including its cross-sectional design, which limits causal inference, and potential sources of bias such as selection

	presentation, and identify unmet needs. The question addresses an important gap in understanding the epidemiology and clinical features of SLE in Malta and has significant implications for healthcare planning and resource allocation.	and completed various questionnaires assessing fatigue, anxiety, depression, pain, sleep quality, and functional disability. Prevalence and incidence of SLE were estimated, and correlations between clinical variables were analyzed. The study design allows for a comprehensive characterization of SLE patients and identification of unmet needs. However, potential sources of bias such as selection bias and recall bias are not fully addressed, and the cross-sectional nature of the study limits causal inference. Further details on patient recruitment and data collection procedures would enhance transparency.	assess relationships between clinical variables. Statistical significance levels and correlation coefficients are reported, aiding in the interpretation of results. However, more information on potential confounders and adjustment techniques would provide a deeper understanding of the findings.	patients. The study identifies a high proportion of female patients, mean age at diagnosis, prevalence of overweight or obesity, disease activity levels, and prevalence of fatigue and anxiety. Correlation analysis highlights associations between clinical variables such as body mass index, disease activity, and functional disability. The identification of unmet needs underscores the importance of addressing these issues in SLE management. However, further exploration of potential moderators and subgroup analyses would provide additional insights.	bias and recall bias. The reliance on self-report measures for assessing clinical variables may introduce bias, and the small sample size may limit the generalizability of findings. Additionally, the study does not address potential confounders comprehensively, and the lack of a control group limits comparison with the general population. Further discussion on these limitations and their implications for interpretation would strengthen the discussion section.
Mahoney et al. (	The research question	The methodology likely	Since this is a review	The results section	The limitations of the

	focuses on understanding how orexin neurons regulate sleep-wake behavior, the consequences of their loss, and the emerging evidence suggesting narcolepsy as an autoimmune disorder. It is clear and relevant, addressing key aspects of narcolepsy pathophysiology and potential therapeutic approaches.	involves a literature review to summarize existing knowledge about orexin neurons, narcolepsy, and autoimmune mechanisms. It may also include discussions of relevant experimental studies and clinical observations. While not explicitly stated, the approach is appropriate for reviewing and synthesizing information from diverse sources to provide a comprehensive overview of the topic.	article, data analysis may involve synthesizing and summarizing findings from various studies, experiments, and clinical observations. Statistical analysis may not be applicable, but the review likely employs qualitative analysis techniques to interpret and contextualize the existing evidence on orexin neuron function, narcolepsy etiology, and autoimmune mechanisms.	likely presents a synthesis of current knowledge regarding orexin neuron function, the consequences of their loss, and the evidence supporting the autoimmune etiology of narcolepsy. It may highlight key findings from experimental studies, clinical observations, and theoretical models that contribute to our understanding of narcolepsy pathophysiology and potential therapeutic strategies.	review may include potential biases inherent in the selected studies, gaps in the current understanding of narcolepsy and orexin neuron function, and uncertainties regarding the autoimmune mechanisms underlying narcolepsy. Additionally, the review may not cover every aspect of the topic comprehensively due to constraints such as space limitations or the focus of the authors' expertise.
Manglick et al. (2013)	The research question is well-defined: to examine the association between self-reported sleep disturbances and treatment response in	The methodology involves collecting sleep data from 166 adolescents (aged 12–18 years) with a diagnosis of DSM-IV depressive	The data analysis appears appropriate, utilizing descriptive statistics and logistic regression modeling to examine the association	The results are presented clearly, indicating that sleep disturbances were highly prevalent in adolescents with	The study acknowledges several limitations, including the reliance on self-reported sleep data, potential biases

adolescents with depressive disorders. The study addresses an important gap in understanding the role of sleep disturbances in treatment outcomes for adolescent depression, with implications for treatment planning and intervention strategies.	disorder who underwent 3 months of treatment (psychosocial and/or pharmacotherapy) in community-based research programs. Sleep disturbances were assessed using the Kiddie Schedule for Affective Disorders and Schizophrenia for School Age Children at three time points: pre-treatment, post-treatment, and 6-month follow-up. The study design allows for longitudinal assessment of sleep disturbances and treatment response, enhancing the robustness of the findings. However, the reliance on self-reported sleep data and the use of subjective assessment tools may introduce bias. Additional details on treatment protocols and adherence would enhance methodological rigor.	between sleep disturbances and treatment response while controlling for potential confounding variables such as gender, treatment group, and comorbid anxiety. The statistical models provide estimates of risk and associations, enhancing the interpretability of the findings. However, more information on model assumptions and sensitivity analyses would strengthen the analysis.	depressive disorders and were associated with poorer treatment response. Persistent sleep disturbances from pre- to post-treatment assessments were positively associated with depression at the 6-month follow-up, suggesting a link between sleep disturbances and long-term outcomes. The findings underscore the importance of considering sleep-related treatments in adolescent depression management. However, further exploration of potential moderators and mediators would provide additional insights.	associated with community-based research programs, and the generalizability of findings to specific treatment settings and age groups. Additionally, the study does not address potential confounding variables comprehensively, and the impact of other treatment modalities (e.g., psychotherapy) on sleep disturbances and treatment response is not assessed. Further discussion on these limitations and their implications for interpretation would strengthen the discussion section.

Manson et al. (2003)	The research question aims to explore the pathogenesis of SLE, emphasizing genetic predisposition, immune dysfunction, autoantibody production, and environmental triggers. The study effectively addresses these aspects, providing a comprehensive overview of current understanding.	Strengths: Utilization of genetic and immunological techniques to explore genetic components and immune dysregulation. Historical and recent epidemiological data, twin studies, and genome-wide screenings add depth to the genetic aspect. Immunological techniques like B-cell and T-cell studies, complement assays, and cytokine profiling strengthen understanding of immune dysfunction. Weaknesses: Lack of specific details on methodology for some studies mentioned, such as the exact techniques used in genetic screenings or the specific protocols for immunological assays.	Strengths: The data analysis involves interpretation of complex genetic associations, complement deficiencies, cytokine profiles, and apoptosis mechanisms. Results from animal models (e.g., SCID mice) and human studies (e.g., B cell depletion therapy outcomes) are discussed critically. Weaknesses: Limited discussion on statistical analyses of genetic linkage studies and cytokine data, which could provide more robust conclusions.	Strengths: Clear presentation of key findings such as genetic susceptibility (HLA associations), role of autoantibodies (ANA, anti-DNA), immune cell dysregulation (B cell overactivity, T cell abnormalities), complement deficiencies, and cytokine imbalances. Highlighting the role of apoptosis and environmental triggers (UV light, viral infections). Weaknesses: Some results are observational or based on correlations, lacking mechanistic insights into pathways or causative relationships.	Strengths: Explicit discussion of limitations such as variability in genetic findings across different ethnic groups, potential biases in twin studies, and challenges in interpreting cytokine roles due to multifunctionality. Weaknesses: Limited discussion on the clinical relevance of some findings (e.g., how genetic or cytokine variations translate into disease severity or treatment response), and lack of prospective data on environmental triggers like viral infections or UV light in human populations.
Martínez-Orozco et al.	The research question	The methodology	The data analysis likely	The results indicate a	While the study

(2014)	aims to investigate the association between narcolepsy with cataplexy and other non-neurological immune-mediated diseases, a topic that has not been extensively studied. By examining the prevalence of comorbid immunopathological diseases in narcolepsy patients, the study addresses an important gap in understanding the potential links between autoimmune and allergic conditions. The question is clearly defined and relevant, providing valuable insights into the broader spectrum of immune-related disorders associated with narcolepsy.	involves assessing 156 narcoleptic patients using various diagnostic methods, including clinical history, physical and neurological examinations, sleep questionnaires, neuroimaging, and human leukocyte antigen typing. Diagnosis confirmation is based on polysomnography and multiple sleep latency tests or hypocretin-1 level measurements. Patients with immunopathological diseases are matched with those without such conditions for gender and age at onset of narcoleptic symptoms. The methodology is comprehensive and suitable for investigating the research question.	involves comparing the prevalence of immunopathological diseases between narcolepsy patients with and without comorbid conditions. Statistical methods, such as chi-square tests or logistic regression, may be used to assess associations and differences in disease prevalence, as well as to examine the severity of cataplexy in patients with comorbid immunopathological diseases. The analysis allows for the evaluation of the primary hypotheses regarding the relationship between narcolepsy and immune-mediated diseases.	high prevalence (16.6%) of comorbid immunopathological diseases in narcolepsy patients, including autoimmune disorders and allergic conditions. Although not statistically significant, patients with narcolepsy and immunopathological diseases tended to be diagnosed at an earlier age. Additionally, cataplexy was found to be more severe in patients with comorbid immunopathological diseases. The results provide important insights into the clinical characteristics and associations of narcolepsy with other immune-related disorders.	provides valuable information on the association between narcolepsy and immunopathological diseases, it has some limitations. The sample size of 156 narcoleptic patients may limit the generalizability of the findings, and the study's retrospective design may introduce bias. Furthermore, the lack of statistical significance in some comparisons may warrant caution in interpreting the results. Additionally, potential confounding variables and the mechanisms underlying the observed associations remain to be elucidated.

Mehra et al. (2017)	The research question addresses the association between subjective and objective sleepiness in heart failure (HF) patients and their interrelationships with biochemical markers. The question is relevant, as subjective versus objective sleepiness in HF remains understudied, and understanding this relationship has potential implications for clinical management.	The methodology involves a comprehensive approach, including polysomnography, Multiple Sleep Latency Testing, questionnaire data collection, and morning phlebotomy for biochemical markers. Linear regression is used for analysis, with adjustments made for potential confounders. The inclusion of biochemical markers adds depth to the analysis, although the small sample size limits the statistical power and generalizability of the findings.	The data analysis appears rigorous, with linear regression used to assess associations between subjective and objective sleepiness measures and biochemical markers. Adjustments for potential confounders are made to enhance the reliability of the results. However, the small sample size may limit the robustness of the statistical analysis and affect the interpretation of the findings.	The results indicate a lack of significant association between mean sleep latency (MSL) and Epworth Sleepiness Scale (ESS), suggesting a discordance between subjective and objective measures of sleepiness in HF patients. However, significant associations are observed between MSL and biochemical markers, specifically cortisol and interleukin-6, highlighting potential mechanistic pathways underlying objective sleepiness. These findings provide valuable insights into the pathophysiology of sleepiness in HF patients.	One limitation of the study is the small sample size, which may limit the generalizability of the findings and affect the statistical power of the analysis. Additionally, the cross-sectional design precludes the establishment of causality, and potential confounders not accounted for in the analysis may influence the results. Further research with larger sample sizes and longitudinal designs is needed to validate the findings and elucidate the underlying mechanisms linking subjective and objective sleepiness in HF.
Meurot et al. (2022)	The research question is clearly stated and				The study acknowledges several

	directly addresses the potential therapeutic effects of liraglutide on osteoarthritis (OA). It aims to examine its anti-inflammatory, analgesic, and anti-degradative effects using in vitro and in vivo experiments. This aligns well with the current understanding of OA pathology and the potential role of inflammation in its progression.	The methodology appears robust, utilizing both in vitro and in vivo experiments to investigate the effects of liraglutide on OA. The use of a sodium monoiodoacetate OA mouse model and various cellular assays (e.g., chondrocytes and macrophages) enhances the comprehensiveness of the study. However, further details on experimental protocols, such as dosages and administration routes, would strengthen the methodology section.	The data analysis seems thorough, as indicated by the reported outcomes of liraglutide treatment on pain-related behavior, inflammatory mediator secretion, gene expression, macrophage polarization, and enzymatic activities. However, without specific details on statistical analyses and control groups, it's challenging to fully evaluate the rigor of the data analysis. Providing such information would enhance the transparency and reproducibility of the study.	The results demonstrate promising effects of liraglutide in alleviating OA-associated pain, reducing inflammation, shifting macrophage polarization, and inhibiting catabolic enzyme activities. These findings support the hypothesis that liraglutide possesses therapeutic potential for OA treatment by targeting multiple pathological processes. However, without detailed statistical analyses and effect sizes, the strength of these results is somewhat limited.	limitations, such as the reliance on animal models, potential differences in human response to liraglutide, and the need for further clinical validation. Additionally, specific details on potential adverse effects, long-term efficacy, and optimal dosages are lacking, highlighting areas for future research. Overall, these limitations are appropriately addressed, providing context for the interpretation of the results.
Miglioranza Scavuzzi	The research question	The methodology	The data analysis	The results are well-	While the study offers

et al. (2022)	addresses the mechanistic basis of the association between the HLA-DRB1*03:01 allele and SLE, filling a significant gap in understanding.	utilizes mouse and human macrophages to investigate the effects of the DRB1*03:01-encoded allelic epitope in the presence of IFN-γ, providing a robust experimental approach.	effectively demonstrates how the allelic epitope triggers various SLE-associated cellular aberrations, supporting the study's conclusions.	supported and provide compelling evidence for a noncanonical, antigen presentation-independent mechanism of HLA-disease association in SLE.	valuable insights, limitations may include the need for further validation in larger cohorts and exploration of other potential contributing factors to SLE pathogenesis.
Mohamed et al. (2014)	The research aims to evaluate the effect of orexin-A on hyperalgesic and cachectic manifestations in an adjuvant-induced arthritis (AIA) rat model. The question is clearly defined and relevant, addressing the potential therapeutic role of orexin-A in managing pain and weight loss associated with rheumatoid arthritis (RA). The study design aligns with the research question, allowing for the assessment of orexin-A's effects on	The methodology involves the induction of AIA in rats and subsequent treatment with orexin-A or dexamethasone. Pain-associated behavior, body mass, hind paw volume, and serum levels of NGF and NPY were assessed as outcome measures. The study design includes appropriate control groups and treatment regimens, allowing for comparisons between normal controls, AIA rats, and AIA rats treated with orexin-A or dexamethasone. The methods are well-	The data analysis includes comparisons between the different treatment groups regarding pain sensation, body mass, hind paw volume, and serum levels of NGF and NPY. Statistical significance is appropriately determined using appropriate tests, and the results are presented clearly in the abstract. The findings indicate that orexin-A treatment reduces pain sensation and NGF levels while increasing body mass and NPY levels in AIA rats,	The results demonstrate that orexin-A treatment leads to a significant reduction in pain sensation and NGF levels, accompanied by increased body mass and NPY levels in AIA rats. In contrast, dexamethasone treatment reduces paw swelling and pain sensation. These findings suggest that orexin-A has hypoalgesic and anti-cachectic properties in the AIA rat model, while dexamethasone exerts potent anti-inflammatory effects.	The study has several limitations, including the use of an animal model of AIA, which may not fully replicate the complexities of human RA. Additionally, the study focuses solely on the short-term effects of orexin-A and dexamethasone treatment, and long-term outcomes are not assessed. The translational relevance of the findings to human RA patients remains to be elucidated. Furthermore, the mechanisms

	pain behavior, body mass, and serum levels of nerve growth factor (NGF) and neuropeptide Y (NPY) in the AIA rat model.	described and suitable for investigating the hypoalgesic and anti-cachectic effects of orexin-A in the AIA rat model.	whereas dexamethasone treatment reduces paw swelling and pain sensation. The analysis supports the conclusions drawn regarding the potential therapeutic effects of orexin-A and dexamethasone in RA.	The results support the hypothesis that orexin-A and dexamethasone may have complementary effects in attenuating the manifestations and complications of RA.	underlying the observed effects of orexin-A and dexamethasone on pain sensation and cachexia are not fully explored. Future studies should address these limitations to further elucidate the therapeutic potential of orexin-A and dexamethasone in RA management.
Nabatian-Asl et al. (2021)	The study aims to evaluate the effects of melatonin supplementation on oxidative stress markers and disease activity in systemic lupus erythematosus (SLE) patients, addressing the pathological significance of oxidative stress in SLE	The study employs a randomized, double-blind, placebo-controlled trial design, providing a robust methodology to assess the effects of melatonin supplementation in SLE patients. However, the small sample size of 32 SLE females and the short duration of the intervention (12 weeks)	Data analysis involves measuring serum malondialdehyde (MDA) and total antioxidant capacity (TAC) before and after the trial, as well as determining disease activity using the Systemic Lupus Erythematosus Disease Activity Index 2000 (SLEDAI-2K).	The study findings demonstrate a significant reduction in serum MDA levels following melatonin supplementation compared to both baseline and placebo groups, indicating a beneficial effect of melatonin in reducing oxidative stress in SLE patients. However, no	The study concludes that melatonin supplementation effectively decreases oxidative stress in SLE patients without significant changes in disease activity. However, further investigations with larger sample sizes and longer intervention durations

	and the potential therapeutic role of melatonin.	may limit the generalizability and long-term assessment of melatonin's effects. Further details on randomization procedures, blinding methods, and participant characteristics would enhance methodological clarity.	Statistical analysis likely includes comparisons of changes in MDA and TAC levels between the melatonin and placebo groups, as well as assessing changes in disease activity scores. Additional subgroup analyses may provide insights into potential modifiers of treatment response.	significant changes are observed in serum TAC levels or disease activity scores between the melatonin and placebo groups. These results contribute valuable insights into the potential antioxidant effects of melatonin in SLE management.	are warranted to validate these preliminary findings and provide more conclusive evidence regarding the therapeutic potential of melatonin in SLE.
Nessaibia et al. (2020)	The research question aims to investigate the psychiatric side effects of chloroquine, particularly in the context of its use against malaria and COVID-19. Specifically, it addresses the lack of awareness regarding chloroquine's psychiatric side effects, which may be overlooked by scientific committees. The question is relevant,	The methodology used in the study is not explicitly outlined in the abstract. However, it likely involves a review of existing literature and case studies to identify and document psychiatric side effects associated with chloroquine use. Given the nature of the research question, a literature review or retrospective analysis of reported cases would be appropriate to gather	The abstract does not mention specific data analysis methods, indicating that the study may focus more on literature review or case presentation rather than quantitative data analysis. Given the focus on psychiatric side effects of chloroquine, data analysis may involve identifying and summarizing reported cases or studies documenting such side	The results section of the abstract highlights several psychiatric side effects associated with chloroquine use, including insomnia, headaches, skin reactions, digestive upset, blurred vision, local pain, increased anxiety, and mental distress. These findings suggest that chloroquine treatment, particularly in high doses and in combination with	The abstract acknowledges several limitations of the study, including the lack of awareness of chloroquine's psychiatric side effects, which may be overlooked by scientific committees. Additionally, the abstract mentions adverse effects associated with hydroxychloroquine and azithromycin combination therapy,

especially considering the widespread use of chloroquine in various medical fields and its recent application against COVID-19. By exploring the potential psychiatric symptoms associated with chloroquine use, the study fills a knowledge gap and raises important considerations for long-term COVID-19 treatment. Overall, the research question is well-defined and addresses an important aspect of chloroquine's safety profile.

evidence on psychiatric symptoms linked to chloroquine. However, without detailed information on the methodology, it is challenging to evaluate the rigor and appropriateness of the approach used in the study. Future studies should provide clear descriptions of the methodology to enhance transparency and reproducibility.

effects. However, without explicit details on data analysis methods, it is challenging to assess the rigor and comprehensiveness of the analysis. Future studies should provide clarity on the data analysis approach used to ensure transparency and validity of findings.

azithromycin, may lead to a range of psychiatric symptoms that could impact patients' well-being and treatment adherence. The results underscore the importance of considering psychiatric side effects when using chloroquine, especially in the context of COVID-19 treatment. Overall, the results provide valuable insights into the potential mental health implications of chloroquine therapy, highlighting the need for further research and clinical monitoring.

which may exacerbate mental distress in patients. However, the abstract does not provide specific details on the methodology used, sample size, or data sources, limiting the ability to assess the robustness of the findings. Moreover, the focus on reported cases or literature review without primary data collection may introduce biases or limitations inherent in secondary data analysis. Future studies should address these limitations by providing transparent methodology, comprehensive data analysis, and consideration of potential biases in the interpretation of results.

Ni et al. (2015)	The research question is well-defined and relevant, aiming to review recent advances in the potential use of interleukin 10 (IL-10) as a novel strategy for treating chronic viral infections and cancer. This addresses an important area of immunotherapy, considering the role of IL-10 in modulating inflammation and its potential therapeutic implications in chronic viral infections and cancer. The question is comprehensive, covering both preclinical and potential clinical applications of IL-10-	The methodology involves a review of recent advances in the use of IL-10 as a therapeutic strategy for chronic viral infections and cancer. While specific details on the literature search strategy and inclusion criteria are not provided, the review approach is appropriate for synthesizing existing evidence and highlighting potential therapeutic avenues. However, the lack of transparency regarding the methodology may limit the reproducibility and reliability of the findings.	As this is a review article, data analysis is primarily qualitative, involving the synthesis and interpretation of recent advances in IL-10-based immunotherapy for chronic viral infections and cancer. The review highlights the increased levels of IL-10 in chronic viral infections and certain cancers, as well as the potential benefits of blocking IL-10 signaling in animal models. While no statistical analyses are conducted, the findings provide valuable insights into the potential therapeutic applications of IL-10	The results of the review suggest that IL-10 plays a significant role in chronic viral infections and cancer, with increased serum levels observed in these conditions. Furthermore, blocking IL-10 signaling at the time of immunization has shown promise in clearing chronic viral infections and preventing tumor growth in animal models. These findings underscore the potential of IL-10-based immunotherapy as a novel strategy for treating chronic viral infections and cancer in humans.	The study acknowledges several limitations, such as the lack of detailed information on the specific mechanisms underlying IL-10-mediated effects in chronic viral infections and cancer. Additionally, while promising preclinical results have been observed, the translation of IL-10-based immunotherapy to human clinical trials may face challenges related to safety and efficacy. Furthermore, potential off-target effects and the need for further optimization of treatment protocols are important considerations. Addressing these limitations would

	based immunotherapy.		in these conditions.		strengthen the potential clinical translation of IL-10-based immunotherapy.
Nokes et al. (2019)	The research question is clearly stated: to evaluate the prevalence of SDB in patients with scleroderma using forehead oximetry and PSG. The hypothesis that scleroderma might be associated with SDB is well-defined.	Strengths: The study used a retrospective cohort design, which allowed for a large sample size (171 patients) over a 10-year period, ensuring a diverse representation from a tertiary center. Data collection was comprehensive, utilizing standard diagnostic tools like pulmonary function tests, echocardiography, and high-resolution CT scans. Weaknesses: The retrospective nature limits control over variables and introduces potential bias in data collection. The reliance	Strengths: Descriptive statistics were clear, detailing abnormal oximetry findings (32.1% prevalence of abnormal findings). Logistic regression was appropriately used to analyze predictors of abnormal oximetry. Weaknesses: Limited dedicated PSG data (only 17 patients) for a deeper analysis. The statistical power might be compromised due to the small sample size in the PSG subgroup.	Strengths: Results are presented clearly, including prevalence rates, median ODI, mean SpO2 values, and demographic characteristics. They effectively support the hypothesis that scleroderma is associated with SDB. Weaknesses: The discussion of results could be more expansive, especially concerning the implications of findings on clinical management and prognosis.	Strengths: The study acknowledges limitations such as its retrospective nature, potential selection bias, and limited PSG availability. It addresses methodological concerns related to oximetry accuracy and patient compliance. Weaknesses: While the limitations are discussed, there could be more detail on the impact of these limitations on the study's conclusions and generalizability.

		on forehead oximetry as a screening tool, while practical due to patient constraints, may not be as sensitive as PSG for detecting SDB.			
Ogata et al. (2019)	The research question aims to evaluate the efficacy, safety, and characteristics of tocilizumab (TCZ), an interleukin-6 (IL-6) inhibitor, for the treatment of rheumatoid arthritis (RA). Specifically, the study explores the effectiveness of TCZ as monotherapy, its impact on systemic inflammatory symptoms, immunogenicity, long-term retention, and adverse events (AEs). By addressing these aspects, the research question provides valuable insights into the clinical utility and limitations of TCZ in	The methodology involves a comprehensive review of evidence from clinical trials, observational studies, and real-world data spanning TCZ's first decade of use for RA treatment. The study synthesizes findings from diverse sources to assess TCZ's efficacy, safety profile, unique characteristics, and clinical implications. While the methodology lacks specific details on search strategies or inclusion criteria, it appears to incorporate a systematic approach to data collection and analysis. However, the absence of explicit methodological details	The data analysis involves synthesizing evidence from various studies and clinical trials to evaluate TCZ's efficacy, safety, and characteristics in RA treatment. The analysis highlights TCZ's advantages, such as its efficacy as monotherapy, improvement of systemic inflammatory symptoms, and low immunogenicity. Additionally, the analysis identifies common adverse events associated with TCZ therapy, including infections and abnormalities in laboratory findings. While the analysis	The results summarize the key findings regarding TCZ's efficacy, safety, and characteristics in RA treatment based on evidence from various studies. Key results include TCZ's efficacy as monotherapy, its impact on systemic inflammatory symptoms, low immunogenicity contributing to long-term drug retention, and common adverse events associated with TCZ therapy. Additionally, the results highlight the challenges and considerations in TCZ therapy, such as the potential for stealth	The study acknowledges several limitations, including the absence of specific methodological details on search strategies or inclusion criteria, which may affect reproducibility and transparency. Additionally, while the review synthesizes evidence from diverse sources, the absence of quantitative measures or statistical analysis limits the depth of data analysis. Furthermore, the review primarily focuses on summarizing existing evidence rather than presenting new findings or conducting

	RA management. Overall, the research question is well-defined and relevant for informing clinical practice and guiding future research on IL-6 inhibitory strategies in RA treatment.	may limit reproducibility and transparency. Nonetheless, the review methodology is appropriate for addressing the research question and synthesizing evidence on TCZ in RA treatment.	provides valuable insights into TCZ's clinical profile, it lacks quantitative measures or statistical analysis, relying instead on qualitative synthesis of evidence. Nonetheless, the data analysis effectively summarizes key findings from the literature and informs the discussion on TCZ's clinical implications in RA management.	infections and lower intestinal perforation. Overall, the results provide a comprehensive overview of TCZ's clinical profile and implications for RA management, effectively summarizing the evidence from the literature.	original research. Despite these limitations, the study provides valuable insights into TCZ's efficacy, safety, and characteristics in RA treatment, serving as a useful resource for clinicians and researchers. Future studies could address the identified limitations and further explore specific aspects of TCZ therapy in RA management.
Omair et al. (2015)	The research question addresses the evaluation of gastrointestinal adverse events of mycophenolate in systemic sclerosis (SSc) patients, as well as secondary outcomes	The methodology involves a systematic literature search of multiple databases to identify studies reporting the use of mycophenolate in SSc patients and relevant outcomes. Inclusion	The data analysis involves summarizing the findings from the included studies regarding the prevalence of gastrointestinal adverse events, other safety outcomes, and	The results summarize findings from 21 included studies involving 487 SSc patients exposed to mycophenolate. The primary outcome of gastrointestinal adverse events is reported, with	The abstract identifies several limitations, including the retrospective nature of the included studies, which may introduce bias and limit the ability to establish causality.

related to safety and effectiveness. The question is relevant and clearly stated, aiming to investigate an important aspect of mycophenolate use in SSc patients. By assessing the prevalence and severity of gastrointestinal adverse events and other safety outcomes, as well as the effectiveness of mycophenolate in skin and lung disease, the study aims to provide valuable insights into the use of this medication in SSc. Overall, the research question is well-defined and appropriate for the study objectives.

criteria are clearly specified, and studies reporting gastrointestinal adverse events, modified Rodnan skin score (MRSS), forced vital capacity (FVC), diffusing capacity of carbon monoxide (DLCO), as well as other safety outcomes, are included. The systematic approach to study selection enhances the reliability and validity of the findings. However, the methodology does not provide details on the search strategy, screening process, or criteria for assessing study quality, which may affect the comprehensiveness of the review. Additionally, the inclusion of observational studies may introduce bias and limit the ability to draw causal inferences. Overall, while the methodology is adequate

effectiveness of mycophenolate in SSc patients. Descriptive statistics are used to present the frequency and types of adverse events, including gastrointestinal events, infections, cytopenias, malignancies, and deaths. The analysis also includes outcomes related to lung and skin involvement, such as FVC and MRSS. While the data analysis provides valuable insights into the safety and effectiveness of mycophenolate in SSc, there is limited detail on statistical methods or adjustments for potential confounders. Additionally, the lack of quantitative synthesis or meta-analysis may limit the ability to draw definitive conclusions. Overall, the data analysis is informative

diarrhea, nausea, and abdominal pain being the most common. Secondary safety outcomes, including infections, cytopenias, malignancies, and deaths, are also presented. Moreover, the effectiveness of mycophenolate in improving or stabilizing lung and skin involvement is discussed based on observational data. The results provide a comprehensive overview of the safety profile and potential benefits of mycophenolate in SSc patients. However, the presentation of results could be enhanced with clearer delineation of findings from individual studies and more detailed reporting of effect sizes or measures of association. Overall, the

Additionally, the lack of detailed information on study quality assessment, search strategy, and screening process may affect the reliability and validity of the findings. The inclusion of observational studies may also introduce confounding variables and limit the ability to draw definitive conclusions. Furthermore, the variability in disease duration and treatment regimens across studies may impact the generalizability of the results. Despite these limitations, the study provides valuable insights into the gastrointestinal adverse events and safety profile of mycophenolate in SSc patients. However, cautious interpretation

		for addressing the research question, additional transparency and rigor would strengthen the study.	but could benefit from more robust statistical methods and reporting.	results effectively address the study objectives and contribute to the understanding of mycophenolate use in SSc.	is warranted due to the inherent limitations of the included studies and the retrospective design.
Orrù et al. (2020)	The research question is clearly defined and addresses the association between obstructive sleep apnea (OSA) and oxidative stress, inflammation, and endothelial dysfunction. The question is relevant and aligns with the study's objective of investigating the pathophysiological mechanisms underlying OSA.	The methodology involves a systematic review using key terms for literature search and inclusion criteria based on established guidelines for OSA diagnosis. However, details regarding the search strategy and selection process are lacking, which could affect the comprehensiveness and reproducibility of the review. Additionally, the inclusion of biomarkers associated with OS, inflammation, and endothelial dysfunction provides a comprehensive approach to the topic. Overall, while the methodology is	The data analysis focuses on synthesizing findings from included studies to elucidate the relationship between OSA and oxidative/nitrosative stress, inflammation, NO production, and endothelial dysfunction. Results are presented descriptively, highlighting significant associations and potential therapeutic implications. However, quantitative analysis or meta-analysis techniques are not mentioned, limiting the depth of data synthesis and interpretation. The	The results summarize key findings from the literature review, highlighting the significant association between nocturnal intermittent hypoxia and various pathophysiological processes in OSA, including oxidative/nitrosative stress, inflammation, and endothelial dysfunction. The contribution of BMI and the beneficial effects of CPAP therapy are also discussed. However, the results lack quantitative data and rely primarily on	The study acknowledges several limitations, including potential biases in the selection of included studies, variations in methodology among studies, and the absence of quantitative data synthesis. The lack of detailed information on the search strategy and selection process is also noted as a limitation, potentially affecting the comprehensiveness and validity of the review findings. Additionally, while CPAP therapy is highlighted as a

		sound, improvements in transparency and detail would enhance its robustness.	analysis could benefit from additional statistical methods to strengthen the conclusions drawn from the literature review.	qualitative synthesis, limiting the precision of the conclusions drawn. Additionally, while the results are informative, they could be enhanced with more detailed descriptions of individual study findings and their implications.	promising intervention, the review does not address potential limitations or challenges associated with its implementation in clinical practice. Overall, while the limitations are appropriately acknowledged, addressing them could strengthen the reliability and generalizability of the study findings.
Ostojic et al. (2013)	The study aims to assess the prevalence and potential causes of RLS in SSc patients, addressing a clinically significant issue in this population.	Strengths: Clear inclusion criteria, comparison with a control group, use of established diagnostic criteria (IRLSSG), and EMNG for neuropathy assessment. Weaknesses: Small sample size (27 SSc patients), single-center study, potential selection bias.	Strengths: Use of statistical tests to compare prevalence rates, demographic and clinical factors influencing RLS. Weaknesses: Limited discussion on severity of RLS, potential for confounding factors not fully explored.	Strengths: Clear presentation of RLS prevalence (40.7% in SSc vs. 4.9% in controls), association with metoclopramide use. Weaknesses: Lack of severity assessment, small subgroup analysis, limited discussion on impact of RLS on patients.	Internal Validity: Small sample size affecting generalizability, potential bias due to single-center recruitment. External Validity: Findings may not be applicable to broader SSc populations. Methodological Considerations: Lack

					of longitudinal data, severity assessment, and impact on quality of life.
Palagini et al. (2014)	The research question focuses on exploring the relationship between insomnia, depression, and systemic lupus erythematosus (SLE), which is clearly stated and relevant to the field. It aims to investigate the prevalence and associations of these conditions.	Strengths: The study likely employed a systematic review methodology, which is appropriate for synthesizing existing literature. It includes a broad range of studies and uses systematic methods for identifying and selecting relevant research. Weaknesses: Methodological details are not explicitly outlined, making it difficult to assess the rigor of the selection process. Clarification of search strategy and inclusion/exclusion criteria would enhance transparency.	Strengths: The systematic review methodology suggests a rigorous approach to data synthesis and analysis, which involves summarizing and synthesizing findings from multiple studies. Weaknesses: Specific details on data analysis techniques (e.g., statistical methods used for synthesis) are not provided, limiting transparency and reproducibility.	Strengths: The study provides a comprehensive overview of the relationship between insomnia, depression, and SLE based on existing literature. It highlights key findings from various studies, such as prevalence rates and associations. Weaknesses: Limited discussion on conflicting results or variability across studies, which could affect the robustness of conclusions drawn.	Strengths: Acknowledges potential limitations such as variability in study methodologies, sample sizes, and quality of included studies. Weaknesses: Specific limitations related to individual studies (e.g., biases, methodological flaws) are not detailed, which could impact the reliability and generalizability of findings.
Park et al. (2016)	The research question is clearly stated: to assess the degree and	The methodology is well-designed, with TMD diagnosis using	The data analysis appears thorough, utilizing appropriate	The results indicate significantly higher levels of plasma	Some limitations may include the reliance on self-report measures

	interrelationship of sleep disturbance and plasma cytokine levels in TMD pain patients.	standardized criteria and matching healthy subjects for comparison.	statistical tests to compare results between different groups and evaluate associations between variables.	cytokines in TMD patients, particularly those with high disability, along with increased sleep disturbance as measured by PSQI and ESS scores.	for sleep quality assessment and the lack of longitudinal data to establish causality. Additionally, the study's generalizability may be limited by its focus on female participants.
Pattison et al. (2004)	The research question aims to investigate the association between red meat consumption and the development of inflammatory polyarthritis, addressing an important gap in understanding potential dietary risk factors for this condition.	The methodology utilizes a nested case-control design within a prospective population-based study, which allows for the investigation of dietary factors while controlling for potential confounders. However, the reliance on self-reported dietary intake via a 7-day food diary may introduce recall bias and measurement error, impacting the accuracy of dietary assessments. Additionally, the matching of cases and controls for age and sex enhances the validity of	The data analysis employs conditional logistic regression to compare the risk of inflammatory polyarthritis between subjects in different tertiles of dietary intake, providing odds ratios with 95% confidence intervals. Adjustment for potential confounders, such as total energy intake and smoking, strengthens the analysis. However, further exploration of dietary patterns and interactions may offer additional insights.	The results indicate higher red meat intake and lower vitamin C intake among patients with inflammatory polyarthritis compared to controls. After adjusting for confounders, subjects with the highest consumption of red meat, meat and meat products combined, and total protein showed an increased risk of developing inflammatory polyarthritis. These findings suggest a potential association between red meat	The conclusion highlights the potential role of high red meat consumption as a risk factor for inflammatory arthritis, though it also acknowledges the possibility of red meat serving as a marker for other lifestyle-related risk factors. This nuanced interpretation underscores the need for further research to elucidate the underlying mechanisms and causal relationships.

		the study.		consumption and the risk of inflammatory arthritis.	
Peters et al. (2011)	The research question is clearly defined, focusing on the prevalence of self-reported sleep disturbance among night smokers and its association with smoking cessation failure. The question addresses an important gap in understanding the relationship between night smoking, sleep disturbance, and smoking cessation outcomes.	The methodology is robust, utilizing a double-blind randomized controlled trial design, which is considered the gold standard for studying treatment interventions. The use of validated measures for assessing sleep disturbance and smoking status enhances the reliability and validity of the findings.	The data analysis appropriately examines the associations between pre-cessation risk factors (night smoking and sleep disturbance) and smoking cessation failure using longitudinal assessments. Statistical techniques appear suitable for addressing the research question and interpreting the results effectively.	The results are clearly presented, indicating that night smokers reported greater sleep disturbance compared to nonnight smokers. Additionally, smokers with co-occurring night smoking and sleep disturbance were at significantly greater risk for smoking cessation failure, highlighting the importance of addressing these factors in cessation interventions.	While the study provides valuable insights, limitations such as the reliance on self-reported measures for sleep disturbance and smoking status, as well as the potential for confounding variables, should be acknowledged. Additionally, the generalizability of the findings may be limited to the specific population and context of the trial.
Pham et Mathis (2018)	The research question is well-formulated, examining the potential dysfunction of the HPA axis in systemic lupus erythematosus (SLE) and its role in chronic inflammation and dysautonomia. The	The methodology is robust, utilizing a well-established animal model (female SLE and control mice) and appropriate stimuli (LPS challenge) to investigate HPA axis activity. Measuring c-Fos	The data analysis is thorough, comparing c-Fos expression, plasma ACTH, and corticosterone levels between SLE and control mice under both baseline and LPS-challenged conditions.	The results reveal significant findings, such as comparable baseline c-Fos, ACTH, and corticosterone levels between SLE and controls, and the heightened corticosterone response	Limitations include the exclusive use of female mice, which may not fully represent gender differences in SLE pathophysiology. The study is also limited to acute inflammatory responses, and long-

	study aims to uncover specific points of dysfunction within the neuro-endocrine-immune pathway, which is crucial for understanding the pathophysiology of SLE.	expression, plasma ACTH, and corticosterone levels provides comprehensive data on neuronal, pituitary, and adrenal responses. The study design allows for detailed analysis of the HPA axis at multiple levels in response to an inflammatory challenge.	The inclusion of multiple time points and controls strengthens the analysis. Statistical comparisons are made to determine significance, providing clear insights into the differential responses between experimental groups.	in LPS-challenged SLE mice. The paradoxical increase in TNF-α despite elevated corticosterone suggests a dysregulated inflammatory response in SLE. These findings contribute to understanding the complex interplay between the HPA axis and immune response in SLE.	term studies could provide additional insights. Moreover, the exact mechanisms behind the paradoxical TNF-α increase remain unclear, and further research is needed to elucidate these pathways. Potential confounding factors, such as genetic variability and environmental influences, are not addressed.
Pollard et al. (2013)	The research question addresses the effect of rituximab on proinflammatory cytokines in primary Sjögren's syndrome (pSS) patients. It is relevant and significant for understanding the therapeutic mechanism of rituximab in pSS.	The study employs a well-designed methodology, involving a randomized, double-blind, placebo-controlled trial with a multiplex-25 bead array assay to analyze cytokines/chemokines in serum. It includes a comparison with age-matched and sex-matched healthy controls.	The data analysis involves statistical tests such as Mann–Whitney U test and linear mixed effect analysis to assess differences in cytokine levels between rituximab-treated and placebo-treated patients. The Wilcoxon matched pairs test is used to compare levels before and after treatment.	The results demonstrate that treatment with rituximab leads to a significant decrease in serum levels of several proinflammatory cytokines/chemokines in pSS patients. These levels gradually return towards baseline over time. Placebo-treated patients show minimal changes in cytokine levels.	The study acknowledges potential indirect effects of rituximab treatment on cytokine levels, such as through B-cell depletion and reduction in autoantibody levels. However, it does not delve into mechanistic explanations in detail or explore the long-term implications of the observed changes.

Pourhabibi-Zarandi et al. (2022)	The research question is clearly defined and relevant: to investigate the effects of curcumin supplementation on metabolic parameters, inflammatory factors, visfatin levels, and obesity values in women with rheumatoid arthritis (RA). This question addresses a significant concern for RA patients, who often suffer from metabolic disorders and inflammation.	The study utilized a randomized, double-blind, placebo-controlled clinical trial design, which is robust and minimizes bias. The sample included 48 women with RA, who were divided into curcumin and placebo groups. Participants received 500 mg of curcumin or placebo once daily for 8 weeks. Data collected included fasting blood samples, anthropometric measurements, dietary intakes, and physical activity levels, both at baseline and at the end of the study.	Data analysis compared metabolic parameters, inflammatory factors, visfatin levels, and obesity values between the curcumin and placebo groups. Statistical significance was determined with a threshold of $p < .05$. Significant decreases were found in HOMA-IR, erythrocyte sedimentation rate, high-sensitivity C-reactive protein, triglycerides, weight, BMI, and waist circumference in the curcumin group compared to placebo. HOMA-IR and triglyceride levels increased in the placebo group.	The results showed that curcumin supplementation significantly improved several metabolic and inflammatory parameters in women with RA compared to the placebo group. Significant reductions in HOMA-IR, inflammatory markers, and certain obesity values were observed. However, no significant changes were found in fasting blood sugar, insulin, other lipid profiles, and visfatin levels. These findings support the potential benefits of curcumin in managing metabolic and inflammatory aspects of RA.	The study has some limitations. The sample size is relatively small, which may limit the generalizability of the findings. The study duration was only 8 weeks, which might not capture long-term effects of curcumin supplementation. Additionally, the study was conducted only on women with RA, so the results may not be applicable to men or to patients with different forms of arthritis. Further research with larger, more diverse populations and longer follow-up periods is necessary to confirm these findings.

Prado et al. (2002)	The research question is clear, relevant, and well-defined, focusing on the impact of the real effective exchange rate on Ghana's trade balance.	The methodology is sound, using an ARDL model to analyze time series data, which is suitable for the research question.	The data analysis is comprehensive, incorporating necessary econometric tests and diagnostics to ensure robustness and validity.	The results are clearly presented and well-interpreted, demonstrating significant relationships and providing insights into both short-term and long-term effects.	The limitations are adequately discussed, acknowledging data constraints and assumptions made, with suggestions for future research to address these issues.
Purani et al. (2019)	The research question is clear and focused, aiming to explore the relationships between exercise, sleep, and smoking in cigarette smokers, addressing an important gap in understanding.	The methodology adequately describes the data collection process and measures used for sleep quality, smoking-related outcomes, and exercise. However, additional details on the study design and participant characteristics would enhance comprehensiveness.	The data analysis employs simple linear regression models, which are appropriate for exploring associations between variables. However, more sophisticated statistical techniques could provide deeper insights into the relationships examined.	The results are well-presented, indicating poor sleep quality at baseline and significant associations between sleep quality and smoking-related outcomes. Additionally, the association between increased exercise and improved sleep quality during follow-up is clearly documented.	While the study provides valuable insights, limitations such as the small sample size, potential for confounding variables, and reliance on self-reported measures could impact the generalizability and robustness of the findings. Including a discussion of these limitations would enhance the transparency of the study.
Redwine et al. (2000)	The research question is clear and relevant, aiming to understand	The methodology is sound, involving 31 healthy male volunteers	Data analysis is thorough, with statistical evaluations	The results indicate that IL-6 levels increase with sleep onset and	The study is limited by its relatively small sample size and focus

	the effects of nocturnal sleep, partial night sleep deprivation, and sleep stages on IL-6 levels, and its relationship with GH, cortisol, and melatonin. This is pertinent given the implications for immune function and inflammatory diseases.	monitored during two different sleep conditions using electroencephalogram polysomnography. Blood samples were taken at regular intervals to measure IL-6, GH, cortisol, and melatonin levels. This approach allows for precise correlation of sleep stages with hormone levels.	showing significant changes in IL-6 levels related to sleep onset and different sleep stages. The study effectively correlates the hormonal profiles with sleep conditions, providing robust insights into the dynamics of IL-6.	are associated with stage 1-2 and REM sleep but not slow wave sleep. Partial sleep deprivation delays the nocturnal rise in IL-6. The findings also show that GH follows a similar profile to IL-6, while cortisol and melatonin do not. These outcomes highlight the complex relationship between sleep and immune function.	on healthy male volunteers, which may not be generalizable to broader populations, including females, older adults, or individuals with health conditions. Additionally, the effects of chronic sleep deprivation were not addressed, limiting the understanding of long-term implications. Further research is needed to explore these aspects and confirm the findings in more diverse populations.
Reid et al. (2019)	The research question is clearly defined and addresses an important gap in knowledge regarding the association between diet quality and obstructive sleep apnea (OSA). It aims to assess potential mediators for this association,	The methodology involves a large, diverse population and utilizes Type 2 in-home polysomnography, enhancing the reliability of sleep data. Additionally, dietary intake is assessed using a validated food frequency questionnaire,	The data analysis employs appropriate statistical techniques, including adjusted analyses to account for potential confounders. The mediation analysis to explore the role of slow-wave sleep in mediating the association between	The results are clearly presented and indicate significant associations between OSA and specific dietary components, as well as overall diet quality. Additionally, the identification of slow-wave sleep as a potential mediator	While the study provides valuable insights, limitations include its observational nature, which precludes establishing causality. Additionally, reliance on self-reported dietary intake and cross-sectional design

	enhancing its relevance.	strengthening the study's methodology.	diet quality and OSA is particularly robust.	adds depth to the findings.	limit causal inference. Further, the generalizability may be limited to older adults in the Multi-Ethnic Study of Atherosclerosis.
Reveille et al. (1998)	The research question is clear and addresses an important gap in the literature by investigating the relative impact of immunogenetic versus socioeconomic factors on systemic lupus erythematosus at disease onset/presentation. The study aims to elucidate the complex interplay between genetic predisposition and social determinants of health in shaping the clinical features of SLE.	The methodology involves the abstraction of medical records from 229 SLE patients enrolled in a prospective lupus outcome study, providing a relatively large sample size for analysis. However, the study could benefit from a more detailed description of the inclusion and exclusion criteria for patient enrollment, as well as the methods used for genetic and socioeconomic data collection. Additionally, the stepwise logistic and multiple linear regression models used for data analysis should be clearly described to enhance transparency	The data analysis utilizes appropriate statistical methods, including stepwise logistic and multiple linear regression models, to examine the associations between immunogenetic and socioeconomic factors and specific clinical features of SLE. Furthermore, the study does not account for potential confounding variables that may influence the relationship between genetic and socioeconomic factors and SLE presentation, which could affect the validity of the findings.	The results demonstrate significant associations between certain genetic and socioeconomic factors and specific clinical features of SLE, highlighting the complex etiology of the disease. The findings suggest that both genetic predisposition and socioeconomic status play important roles in shaping the presentation of SLE, with differences observed among different ethnic groups. However, the lack of detailed statistical information limits the interpretation of the results and the	While the study sheds light on the interplay between immunogenetic and socioeconomic factors in SLE presentation, several limitations warrant consideration. These include the retrospective design relying on medical record abstraction, potentially introducing biases and incomplete data. Additionally, the study's cross-sectional nature and focus on specific factors may limit generalizability and overlook other determinants, highlighting the need for further research to

		and reproducibility.		generalizability of the findings.	address these gaps.
Riemann et al. (2017)	The research question aims to provide clinical recommendations for the diagnosis and treatment of insomnia in adult patients. It is comprehensive and relevant to clinicians involved in managing insomnia. By synthesizing evidence from relevant meta-analyses, the guideline addresses key aspects of insomnia management, including diagnostic procedures, treatment options, and recommendations for different patient populations. Overall, the research question is well-defined and addresses an important clinical need, making it valuable for guiding practice in the management of insomnia.	The guideline development process involves a systematic review of relevant meta-analyses published until June 2016. The European Sleep Research Society's task force used the GRADE system to grade the evidence and guide recommendations. The methodology is robust and systematic, ensuring that the guideline is based on the best available evidence. The inclusion of meta-analyses and the use of a standardized grading system enhance the credibility and reliability of the guideline. Moreover, the involvement of a multidisciplinary team of experts strengthens the validity of the recommendations. Overall, the	The guideline synthesizes evidence from meta-analyses and applies the GRADE system to grade the evidence and formulate recommendations. Data analysis involves evaluating the quality of evidence and translating it into actionable recommendations for clinical practice. The use of a standardized grading system enhances the clarity and transparency of the recommendations. Additionally, the guideline provides specific recommendations for diagnostic procedures, treatment options, and the use of different interventions based on the strength of	The results of the guideline outline evidence-based recommendations for the diagnosis and treatment of insomnia in adult patients. Key findings include the recommendation of cognitive behavioural therapy for insomnia (CBT-I) as the first-line treatment for chronic insomnia in adults, with pharmacological interventions offered if CBT-I is not effective or available. The guideline also provides recommendations regarding the use of benzodiazepines, benzodiazepine receptor agonists, antidepressants, and other medications for insomnia treatment, based on the strength of evidence.	While the guideline provides valuable recommendations based on the available evidence, there are some limitations to consider. The guideline relies on meta-analyses published until June 2016, and newer evidence may have emerged since then. Additionally, the quality of evidence varies across different interventions, with some recommendations based on low- to very-low-quality evidence. Moreover, the guideline acknowledges the need for further evaluation of certain interventions, such as light therapy and exercise, to determine

		methodology is rigorous and transparent, providing a solid foundation for the guideline's development.	evidence. The data analysis is systematic and rigorous, ensuring that recommendations are based on the best available evidence. Overall, the guideline's approach to data analysis is comprehensive and aligns with best practices in evidence-based medicine.	Furthermore, the guideline evaluates the effectiveness of light therapy, exercise, and complementary and alternative treatments for insomnia. The results are presented clearly and provide valuable guidance for clinicians involved in managing insomnia. Overall, the results offer evidence-based recommendations to optimize the diagnosis and treatment of insomnia in adult patients.	their usefulness in insomnia treatment. Additionally, the guideline may not address specific considerations for subpopulations or special situations, which could limit its applicability in certain clinical scenarios. Despite these limitations, the guideline offers valuable insights and recommendations for the management of insomnia in adult patients.
Rossman (2019)	The research question addresses the prevalence of sleep difficulties in adults and the effectiveness of cognitive-behavioral therapy for insomnia	The methodology involves reviewing existing literature on sleep difficulties, insomnia prevalence, and the efficacy of CBT-i as a treatment. The	The article does not present original data or conduct specific data analysis. Instead, it synthesizes information from existing studies, meta-	The results highlight the prevalence of sleep difficulties and insomnia in adults, the effectiveness of CBT-i as a nonpharmacological	The article acknowledges two main limitations: the shortage of trained CBT-i practitioners and the influence of drug advertisements

(CBT-i) as a nonpharmacological treatment. It highlights the underutilization of CBT-i and the barriers to its implementation. The question is clear, relevant, and important for addressing a prevalent health problem. By emphasizing the benefits of CBT-i and the role of healthcare providers in promoting its use, the research question aims to improve the management of chronic insomnia. Overall, the research question effectively identifies a key issue in sleep medicine and proposes a solution for improving patient care.

information presented is based on empirical evidence and expert consensus in the field of sleep medicine. The article provides an overview of the key components of CBT-i and its effectiveness compared to sleep medication. Additionally, the methodology highlights the barriers to CBT-i implementation, including the shortage of trained practitioners and the influence of drug advertisements. While the methodology lacks specific details on data collection and analysis, it effectively synthesizes existing knowledge to inform healthcare providers about the benefits of CBT-i for insomnia management. Overall, the methodology is appropriate for addressing the research

analyses, and expert opinions to support its arguments. The data presented include prevalence rates of sleep difficulties and insomnia, comparisons between CBT-i and sleep medication efficacy, and barriers to CBT-i utilization. The analysis involves interpreting and synthesizing this information to emphasize the effectiveness and underutilization of CBT-i in clinical practice. While the article lacks detailed statistical analysis, it effectively presents evidence-based arguments to support its recommendations. The focus is on synthesizing existing knowledge to inform healthcare providers about the benefits of CBT-i and the need for

treatment, and the barriers to its implementation. Specifically, the article emphasizes the benefits of CBT-i compared to sleep medication, including equivalent efficacy, fewer side effects, and long-term improvements in sleep quality. The results underscore the importance of educating patients about CBT-i and recommending it as a first-line treatment for chronic insomnia. While the article does not present new findings, it effectively synthesizes existing evidence to convey key messages to healthcare providers. Overall, the results provide valuable insights into the benefits and underutilization of CBT-i in insomnia management.

on patient preferences. These limitations contribute to the underutilization of CBT-i as a treatment for chronic insomnia. However, the article does not discuss potential limitations of CBT-i itself, such as accessibility, cost, or patient adherence. Additionally, while the article advocates for the promotion of CBT-i by healthcare providers, it does not provide specific strategies or recommendations for addressing the identified barriers. Despite these limitations, the article effectively highlights the need for greater awareness and implementation of CBT-i in clinical practice. Future research could explore additional barriers to

		question and providing evidence-based recommendations.	its promotion as a first-line treatment for chronic insomnia. Overall, the data analysis is appropriate for the nature of the article and its objectives.		CBT-i utilization and strategies for overcoming them to improve patient access to evidence-based insomnia treatment. Overall, while the article provides valuable insights, its discussion of limitations could be expanded to include a more comprehensive assessment of challenges in CBT-i implementation.
Ryan et al. (2015)	The research question is well-defined: to explore psychological variables predicting pain-related outcomes (pain severity and pain interference) and psychological outcomes (depression and anxiety) among rheumatoid arthritis	The methodology involves administering online questionnaires to 317 RA patients with chronic pain, collecting data on predictor and outcome variables related to self-concealment, satisfaction of psychological needs (autonomy, relatedness,	The data analysis appears appropriate for the study objectives, utilizing hierarchical multiple linear regressions to examine the predictive models for each outcome variable. Statistical significance levels are reported, indicating the	The results are presented clearly, indicating that the predictive models for each outcome variable were significant and had good levels of fit with the data. Higher relatedness predicted lower depression, and higher autonomy	The study acknowledges several limitations, including the reliance on self-report measures, potential biases inherent in online surveys, and the cross-sectional design limiting causal inference.

	(RA) patients experiencing chronic pain. The question addresses an important gap in understanding the psychological factors contributing to the well-being of RA patients with chronic pain, with implications for intervention development.	competence), pain severity, pain interference, depression, and anxiety. Hierarchical multiple linear regressions were conducted to analyze the data. The study design allows for a comprehensive exploration of the relationships between psychological variables and outcomes in RA patients with chronic pain. However, the reliance on self-report measures and the cross-sectional nature of the study may introduce bias and limit causal inference. Additional details on questionnaire selection and validation would enhance methodological rigor.	significance of the predictor variables in explaining the variance in outcome variables. The study demonstrates good levels of fit with the data, enhancing the reliability of the findings. However, more information on potential confounding variables and sensitivity analyses would strengthen the analysis.	predicted lower anxiety, suggesting the importance of these psychological factors in promoting well-being among RA patients with chronic pain. The findings highlight potential targets for interventions aimed at reducing depression and anxiety in this patient population. However, further exploration of other potential predictors and moderators would provide additional insights.	Additionally, the study does not address potential confounders comprehensively, and the generalizability of findings may be limited due to the specific sample characteristics. Further discussion on these limitations and their implications for interpretation would strengthen the discussion section.
Saevarsdottir et al. (2015)	The research question is clearly defined, aiming to identify clinical predictors for	The study utilizes data from the SWEFOT trial, a prospective cohort design, which allows for	Data analysis is robust, employing multivariable logistic regression to assess the	The results are clearly presented, identifying baseline erosions, erythrocyte	While the study provides valuable insights into predictors of radiographic

	radiographic progression after 1 year in early rheumatoid arthritis (RA) patients. This addresses an important aspect of disease management and treatment planning.	the assessment of baseline predictors of radiographic progression in RA patients over a defined follow-up period. Multivariable logistic regression analysis is appropriate for identifying independent predictors while adjusting for potential confounders.	association between baseline parameters and radiographic progression after 1 year. Adjustments are made for potential confounders, enhancing the validity of the results. Secondary exploratory analyses are conducted to further investigate the predictive value of certain parameters.	sedimentation rate, C-reactive protein, and current smoking as independent predictors of radiographic progression in early RA. The findings remained significant after adjustment for treatment strategy. Secondary exploratory analyses provide additional insights into the predictive value of rheumatoid factor and anti-cyclic citrullinated peptide positivity.	progression in early rheumatoid arthritis (RA), its reliance on a single trial population may limit the generalizability of the findings to broader RA populations. Additionally, the potential for unmeasured confounding factors or biases in radiographic assessment methods could impact the accuracy and reliability of the identified predictors.
Safy-Khan et al. (2021)	The research question is well-defined, aiming to investigate whether current smoking affects the clinical response to concomitant prednisone in a methotrexate (MTX)-based treatment strategy for early rheumatoid arthritis (RA). This addresses an important aspect of	The study utilizes data from the CAMERA-II trial, a randomized controlled trial, providing a robust study design for assessing the clinical response to different treatment strategies in early RA patients. Linear mixed modeling is an appropriate statistical method for analyzing	Data analysis is thorough, employing linear mixed modeling to assess the association between current smoking status and DAS28 over time, while adjusting for relevant covariates. The interaction between current smoking and treatment strategy (prednisone or placebo)	The results are clearly presented, demonstrating that current smoking is significantly associated with higher DAS28 over time in patients with early RA, independent of treatment strategy (prednisone or placebo). The interaction analysis	The study's reliance on self-reported smoking status may introduce recall bias or misclassification, potentially affecting the accuracy of the findings. Additionally, the limited generalizability of results to broader populations of rheumatoid arthritis

	treatment effectiveness and potential interactions with smoking status.	longitudinal data, allowing for the examination of the association between current smoking status and Disease Activity Score assessing 28 joints over time.	is tested to evaluate potential differences in the effect of smoking on clinical response between the two groups.	indicates that the negative effect of current smoking on DAS28 is consistent across both strategy groups, with no significant difference observed. Furthermore, the dose-dependent effect of smoking on DAS28 is noted.	patients and potential unmeasured confounding factors warrant cautious interpretation of the conclusions.
Sahebari et al. (2021)	The research question is clearly defined and relevant, aiming to explore the relationship between sleep disorders and systemic lupus erythematosus in newly diagnosed patients. It seeks to determine if these disorders are related to disease progression or secondary to the disease and its complications.	Strengths: The cross-sectional case-control study design is appropriate for comparing newly diagnosed SLE patients with matched healthy controls. Detailed inclusion and exclusion criteria help to control confounding factors. Weaknesses: The sample size is relatively small (28 females), which may limit generalizability. The study relies on self-reported sleep history, which can introduce bias.	Strengths: Statistical analysis is thorough, using SPSS software to present quantitative and qualitative variables. Pearson correlation coefficient and other relevant tests (e.g., t-test, Mann-Whitney U test) are appropriately used. Weaknesses: The small sample size and lack of detailed methodological steps for data collection and analysis limit the robustness of the findings.	Strengths: The study presents comprehensive results on the relationship between serum levels of prolactin and melatonin and sleep disorders in SLE patients. It finds significant correlations in specific sleep-related items (e.g., sleep latency to N1 and spontaneous arousal index) with melatonin levels in the case group. Weaknesses: There are no significant differences found between the SLE	Strengths: The study acknowledges potential limitations such as the small sample size, the reliance on self-reported data, and the new diagnosis status of the patients, which might not capture the chronic effects of SLE on sleep. Weaknesses: It does not detail potential biases in sample selection or the impact of geographical and cultural factors on sleep disorders. Further, it lacks a longitudinal approach

				patients and the control group regarding sleep disorders, which may be due to the small sample size and the new diagnosis status of the patients.	to assess changes over time in the same patients.
Saidi et al. (2021)	The study investigates how energy balance affects sleep in adolescents, focusing on those with obesity and normal weight controls. Understanding this relationship is crucial for devising strategies to improve sleep in these populations, given the prevalence of sleep issues among adolescents with obesity.	The study employs a well-structured experimental setup involving 28 male adolescents, half with obesity and half normal weight, who undergo ad libitum or eucaloric meals for three days in random order, followed by in-home polysomnography. This approach provides valuable insights into how dietary intake impacts sleep quality.	Data analysis reveals significant interactions between energy intake, dietary conditions, and sleep outcomes. Notably, adjusting dietary intake to energy requirements and reducing evening meal intake led to sleep improvements in adolescents with obesity. These findings underscore the importance of meal timing for sleep quality and suggest potential avenues for targeted interventions.	The results suggest that aligning dietary intake with energy requirements and reducing evening meal intake can benefit sleep quality in adolescents with obesity. However, the study also acknowledges that other factors may influence sleep differences between obese and normal weight adolescents. Nonetheless, these findings offer insights into interventions to improve sleep in adolescents with obesity.	Limitations include the study's small sample size and focus on male adolescents, which may limit generalizability. Additionally, the short-term nature of the dietary interventions and the lack of exploration into long-term effects on sleep quality are notable limitations. Future research could address these by including larger and more diverse samples and investigating the long-term impact of dietary interventions on sleep in adolescents with obesity.

Santilli et al. (2021)	The study effectively addresses the prevalence and diagnostic challenges of OSAS, emphasizing the role of dentists.	The study uses a single-center retrospective design. This limits the generalizability of the findings (applicability to other populations) and introduces selection bias (patients referred to this center might have different characteristics).	Descriptive statistics were used to analyze data, but the lack of specific diagnostic questions for OSAS limits insights.	Potential for missing data in medical records.<br>Reliance on self-reported sleep apnea, which might be inaccurate. The study found a surprisingly low prevalence of OSAS (0.26%), highlighting the inadequacy of current medical records.	The retrospective nature, absence of specific OSAS diagnostic questions, and reliance on self-reported medical history limit the findings.
Schenck et al. (1996)	The research question aims to investigate the association between specific HLA class II antigens and rapid eye movement (REM) sleep behavior disorder in white men without narcolepsy. The question addresses an important aspect of genetic susceptibility to REM sleep behavior disorder and provides a focused direction for the study, highlighting the relevance of HLA	The methodology involves HLA class II antigen typing in 25 white men with REM sleep behavior disorder, excluding those with narcolepsy. The comparison group consists of a local white population, and relevant statistical tests are conducted to assess the association between HLA antigens and REM sleep behavior disorder. The approach is appropriate for	Data analysis includes comparing the frequency of DQw1 antigen positivity in individuals with REM sleep behavior disorder to that in the local white comparison group and published rates in other white populations. Statistical significance is assessed using appropriate tests to determine the strength of association between specific HLA class II alleles and REM	The results indicate that 84% of men with REM sleep behavior disorder were DQw1 positive, with DQB10501 and DQB10602 being the most common phenotypes. This prevalence is significantly higher than that in the local white comparison group and other published white populations, suggesting a strong	The study's limitations may include the small sample size of individuals with REM sleep behavior disorder, which could affect the generalizability of the findings. Additionally, the study's retrospective design may limit the ability to establish causality or account for potential confounding factors. Further validation in larger, prospective

	class II genes in the disorder's pathogenesis.	investigating genetic associations and comparing allele frequencies between affected individuals and controls.	sleep behavior disorder.	association between specific HLA class II genes and REM sleep behavior disorder.	cohorts is needed to confirm the observed associations and investigate potential interactions with other genetic or environmental factors.
Schletzbaum et al. (2021)	The research question aims to identify patient- and health care-level predictors of smoking cessation in patients with rheumatoid arthritis (RA) to guide the implementation of smoking cessation interventions. This question addresses an important aspect of RA management, considering smoking as a risk factor for disease severity.	The study utilizes electronic health record data from two health systems, providing a large sample size for analysis. Multivariable logistic regression is an appropriate statistical method for determining predictors of smoking cessation while adjusting for potential confounders. Exclusion criteria are applied to ensure data quality.	Data analysis is robust, employing multivariable logistic regression to identify predictors of smoking cessation. Adjusted odds ratios and 95% confidence intervals are reported for each predictor, allowing for the assessment of the strength and significance of associations.	The results are clearly presented, indicating that patient sociodemographic factors such as race, age, and insurance status were not significant predictors of smoking cessation in patients with RA. Instead, factors related to health care, such as being new to rheumatology care and receiving care in a rural community health system, were associated with increased likelihood of smoking cessation. Seropositive	While the study provides valuable insights into predictors of smoking cessation in patients with RA, several limitations should be considered. Firstly, the retrospective nature of the study using electronic health record data may introduce inherent biases and limitations in data collection. Secondly, the study's reliance on electronic health records may result in

				patients were less likely to quit smoking.	underreporting or misclassification of smoking status and other variables. Finally, the study's generalizability may be limited to patients within the specific health systems analyzed, potentially affecting the applicability of findings to broader populations.
Schütz et al. (2003)	The research question is clearly stated: to assess sleep patterns in rats injected with Freund's adjuvant in the temporomandibular joint (TMJ) as an experimental orofacial pain model and evaluate the pain response to indomethacin. The question is relevant and addresses an important aspect of pain research,	The methodology involves an experimental study utilizing rat models implanted with electrodes to record electrocorticogram and electromyogram signals for sleep monitoring. Rats were injected with Freund's adjuvant (orofacial pain group) or saline (sham group) in the TMJ, and sleep patterns were assessed over two 12-hour light periods. Indomethacin administration and its	The data analysis appears appropriate for the study objectives, utilizing statistical tests to compare sleep parameters between the orofacial pain group and the sham group, as well as before and after indomethacin treatment. Statistical significance levels are reported, aiding in the interpretation of results. However, more information on the specific statistical	The results are presented clearly, providing information on sleep disturbances observed in rats injected with Freund's adjuvant in the TMJ and the effects of indomethacin treatment on sleep patterns. The inclusion of statistical significance levels and percentage changes in sleep parameters aids in understanding the magnitude of effects.	The study acknowledges several limitations, including its reliance on animal models, which may not fully reflect human conditions. The use of a small sample size may limit the generalizability of findings, and potential confounders such as anesthesia effects are not fully addressed. Additionally, the study's focus on indomethacin as the

	providing insights into the relationship between pain and sleep disturbances.	effects on sleep patterns were also evaluated. The study design allows for the investigation of sleep disturbances related to orofacial pain and the potential therapeutic effects of indomethacin. However, further details on randomization procedures and potential sources of bias would enhance transparency.	methods used and adjustments for potential confounders would enhance the robustness of the findings.	However, more information on effect sizes and confidence intervals would provide a clearer interpretation of the results.	sole treatment option may limit the exploration of alternative therapeutic interventions. Further discussion on these limitations and their implications for interpretation would strengthen the discussion section.
Shaikh et al. (2020)	The research question is clearly defined and significant, focusing on the prevalence of metabolic syndrome in young patients with rheumatoid arthritis (RA). This is important given the strong association between metabolic syndrome and RA, and the increased cardiovascular complications and	The methodology is appropriate for the research question, employing a cross-sectional study design in a tertiary care hospital setting. The consecutive inclusion of diagnosed RA patients over a specified period (April to August 2018) ensures a representative sample. The study assesses RA disease activity using the Clinical Disease Activity	The data analysis is comprehensive, identifying significant associations between metabolic syndrome and various factors such as age, BMI, waist circumference, fasting blood sugar, systolic and diastolic blood pressure, triglycerides, high-density lipoprotein, and methotrexate drug history. The use of p-	The results indicate that 32.7% of the young RA patients studied have metabolic syndrome, with a significant proportion being seropositive. The significant associations found with age, BMI, waist circumference, FBS, SBP, DBP, TG, HDL, and methotrexate history provide valuable insights into the factors contributing	Limitations include the cross-sectional design, which limits the ability to establish causality. The study's setting in a single tertiary care hospital may limit the generalizability of the findings to other populations and healthcare settings. The relatively small sample size (104 patients) could affect the robustness and

	mortality in this patient group. By evaluating the prevalence and associated factors of metabolic syndrome in young RA patients, the study addresses a crucial aspect of managing RA to reduce cardiovascular risks and improve patient outcomes.	Index (CDAI) and measures various determinants and outcome variables relevant to metabolic syndrome. However, being a cross-sectional study, it can only establish associations, not causations. The sample size of 104 patients is reasonable but could be larger for more robust results.	values to determine statistical significance adds rigor to the analysis. The study effectively highlights the high prevalence of metabolic syndrome in young RA patients and its significant associations with multiple metabolic risk factors.	to metabolic syndrome in this population. These findings underscore the need for comprehensive management strategies in young RA patients to address metabolic syndrome and reduce cardiovascular risk. The study successfully identifies key factors associated with metabolic syndrome in young RA patients.	precision of the results. Additionally, the study does not explore potential mechanisms linking RA and metabolic syndrome, nor does it consider the potential impact of other RA treatments besides methotrexate. Longitudinal studies with larger, more diverse populations are needed to confirm these findings and explore underlying mechanisms.
Stipelman et al. (2013)	The research question is clear and addresses an important gap in the literature by examining the relationship between cigarette smoking and sleep in individuals diagnosed with a chronic rheumatic condition. However, the question could be more specific about the potential	The methodology utilizes cross-sectional survey data from the 2007 National Health Interview Survey, which provides a large population sample for analysis. However, the study could benefit from a more detailed description of the survey instrument used to assess smoking status,	The data analysis compares smoking status and sleep outcomes among individuals with and without a chronic rheumatic condition, providing valuable insights into the association between these variables. However, the analysis could be strengthened	The results demonstrate that individuals diagnosed with a chronic rheumatic condition are more likely to be former or current smokers and experience poorer sleep quality compared to those without such a diagnosis. The findings also suggest an	The study acknowledges limitations such as its cross-sectional design, which precludes the establishment of causality or temporality between smoking, sleep, and rheumatic conditions. Additionally, the reliance on self-reported survey data

	mechanisms underlying this relationship and the implications for intervention strategies.	sleep outcomes, and functional impairment related to the chronic rheumatic condition. Additionally, the methodology lacks information on potential confounding variables that may influence the relationship between smoking, sleep, and rheumatic conditions.	by controlling for potential confounders such as age, sex, comorbidities, and socioeconomic status.	additive relationship between smoking and sleep disturbances in individuals with a chronic rheumatic condition, highlighting the importance of addressing both health behaviors in clinical practice. However, the lack of information on effect sizes and confidence intervals for the observed associations limits the interpretation of the results.	may introduce recall bias and social desirability bias, potentially influencing the accuracy of smoking status and sleep outcomes. Furthermore, the study does not account for potential confounding variables, which may affect the validity of the observed associations. While the limitations are acknowledged, further discussion on their implications for the interpretation of results would enhance the transparency of the study.
Straub et al. (2017)	The research question is clearly defined, focusing on the interrelation between sleep parameters, inflammation, and HPA axis activity in rheumatoid arthritis (RA) patients. The	The methodology is appropriate for the research question, employing a prospective study design over 16 weeks with RA patients undergoing intensified treatment. The use of polysomnography to	The data analysis is thorough, examining changes in clinical characteristics, CRP levels, sleep parameters, and HPA axis outcomes. The correlations between serum cortisol, ACTH	The results demonstrate significant improvements in clinical characteristics, sleep efficiency, and wake time after sleep onset, particularly in the ETA group. The study finds no	Limitations include the small sample size and the open study design, which may affect the generalizability and introduce potential biases. The study only includes RA patients with moderate to high

	study aims to understand how treatments with methotrexate (MTX) and etanercept (ETA) impact these variables, which is relevant for improving quality of life in RA patients.	objectively measure sleep parameters, along with clinical and laboratory assessments, provides comprehensive data. However, the study's open design may introduce biases, and a larger sample size could enhance the robustness of the findings.	levels, and sleep parameters are carefully analyzed, both before and after controlling for CRP. Statistical significance is appropriately assessed, providing insights into the relationships between inflammation, HPA axis activity, and sleep quality.	significant changes in serum cortisol and ACTH levels during the observation period. The correlations between cortisol levels and sleep parameters suggest a complex interplay influenced by inflammation, highlighting the potential impact of RA treatments on sleep quality through inflammatory pathways.	disease activity (DAS28CRP ≥3.2), which may not represent all RA patients. The focus on short-term outcomes (16 weeks) limits the understanding of long-term effects. Additionally, the study does not explore other potential confounding factors, such as medication adherence or lifestyle factors.
Sun et al. (2018)	The research question explores the expression and physiological function of orexin A in fibroblast-like synoviocytes (FLSs), particularly in the context of rheumatoid arthritis (RA). It aims to investigate the potential anti-inflammatory effects of orexin A on FLSs and its implications for RA treatment. The question	The methodology involves detecting the expressions of orexin type 1 and type 2 receptors (OX1R and OX2R) on FLSs and investigating the physiological function of orexin A in RA-FLSs and tumor necrosis factor-α (TNF-α)-treated FLSs. The study design includes in vitro experiments to assess the effects of orexin A on	The data analysis involves detecting the expressions of OX1R and OX2R on FLSs, assessing changes in OX1R expression in RA-FLSs and TNF-α-treated FLSs, and evaluating the effects of orexin A treatment on various inflammatory markers and signaling pathways. The study effectively analyzes the impact of orexin A on	The results demonstrate the presence of OX1R but not OX2R on FLSs, with decreased expression of OX1R in RA-FLSs and TNF-α-treated FLSs. Orexin A treatment shows anti-inflammatory effects by reducing the secretion of pro-inflammatory cytokines (IL-1β, IL-6, IL-8), ROS production, and MMP expression in	The study acknowledges several limitations, including its reliance on in vitro experiments which may not fully recapitulate the complex inflammatory environment in RA joints. The findings need to be validated in animal models and clinical studies to assess the translational relevance to human

	is relevant and addresses a gap in understanding the role of orexin A in RA pathogenesis and therapy.	pro-inflammatory cytokine secretion, reactive oxygen species (ROS) production, and matrix metalloproteinase (MMP) expression, as well as the inhibition of the nuclear factor-κB (NF-κB) signaling pathway. Overall, the methodology is appropriate for addressing the research question.	cytokine secretion, ROS production, MMP expression, and NF-κB activation in FLSs. The results are presented clearly and support the conclusions drawn from the data.	FLSs. Mechanistically, orexin A inhibits TNF-α-induced activation of the NF-κB signaling pathway. The results support the potential therapeutic application of orexin A in RA by modulating inflammatory responses in FLSs.	RA. Additionally, the study focuses solely on the effects of orexin A on FLSs, neglecting potential interactions with other cell types and signaling pathways involved in RA pathogenesis. Despite these limitations, the study provides valuable insights into the anti-inflammatory properties of orexin A and its potential as a therapeutic target in RA.
Suzuki et al. (2016)	The research question is clearly defined: to assess the effect of krill oil on mild knee pain. This question is relevant and significant as it aims to explore a potential therapeutic	The study employed a randomized, double-blind, parallel-group, placebo-controlled design, which is the gold standard for clinical trials and helps to minimize bias. The	Data analysis included both primary and secondary outcomes. The improvements in subjective symptoms of knee pain were assessed using the JKOM and JOA	The results showed that krill oil administration led to significant improvements in knee pain and stiffness compared to placebo, particularly in terms of	The study has several limitations. The sample size is small, which may limit the generalizability of the results and the statistical power of the study. The study

option for individuals experiencing mild knee pain, which can impact quality of life and daily functioning. The study addresses a gap in the literature regarding the specific effects of krill oil on this condition.	sample size was relatively small (fifty adults), and participants were randomized to receive either krill oil or placebo for 30 days. Primary and secondary outcomes were clearly defined and measured using standardized tools (JKOM, JOA, blood, and urine biochemical parameters). The study population consisted of adults aged 38-85 years with mild knee pain, which is appropriate for the research question.	questionnaires. The study reported significant improvements in the krill oil group compared to the placebo group for specific measures of knee pain and stiffness. Statistical analyses controlled for confounding factors such as age, sex, weight, and smoking and drinking habits. The use of appropriate statistical methods (e.g., controlling for confounders, significance testing) enhances the reliability of the findings.	pain during sleep, standing, and range of motion. Additionally, krill oil increased plasma EPA and EPA/AA ratio, suggesting a biochemical mechanism for the observed clinical effects. The findings support the potential efficacy of krill oil for reducing mild knee pain, contributing to the existing evidence on its anti-inflammatory properties. However, both the placebo and krill oil groups showed improvements, indicating a potential placebo effect.	duration was relatively short (30 days), which may not capture long-term effects or safety concerns associated with krill oil administration. The study population was limited to a single clinic in Japan, which may affect the applicability of the findings to other populations. Additionally, the subjective nature of pain assessment and the significant improvements in the placebo group suggest that placebo effects may have influenced the results. These limitations should be considered when interpreting the study findings and extrapolating them to broader clinical contexts.

Suzuki et al. (2018)	The research question investigates the role of the orexin system in systemic lupus erythematosus (SLE) patients with hypothalamic lesions presenting with excessive daytime sleepiness (EDS). This study addresses a rare but clinically significant manifestation in SLE patients and explores potential mechanisms underlying EDS in this population. The objective is clear and relevant to understanding the pathophysiology of EDS in SLE.	The methodology involves measuring cerebrospinal fluid (CSF) orexin-A levels in four SLE patients with hypothalamic lesions detected by MRI. Patients underwent repeated CSF testing, and various markers including serum anti-aquaporin-4 antibodies, CSF myelin basic protein, and CSF oligoclonal bands were assessed. All patients met the updated American College of Rheumatology criteria for SLE. The study design is appropriate for investigating the research question, although the sample size is small, limiting generalizability.	The data analysis entails descriptive reporting of CSF orexin-A levels, MRI findings, and clinical presentation in SLE patients with hypothalamic lesions. Changes in orexin-A levels over time and their association with EDS and MRI findings are explored. The analysis effectively characterizes the relationship between orexin-A levels, hypothalamic lesions, and EDS, providing insights into potential mechanisms underlying EDS in SLE.	The results demonstrate low to intermediate CSF orexin-A levels in three SLE patients with hypothalamic lesions and EDS, with subsequent increases in orexin-A levels associated with improvements in EDS and MRI findings. One patient exhibited normal orexin-A levels but elevated CSF interleukin-6 levels, suggesting cytokine-mediated tissue damage as a potential mechanism for EDS in the absence of orexinergic involvement. The findings support the role of the orexin system in EDS in SLE patients with	The study acknowledges limitations such as the small sample size and the lack of a control group. The absence of objective measures of sleepiness and the reliance on subjective reporting of EDS may introduce bias. Additionally, the study does not investigate other potential contributors to EDS in SLE patients, such as medication effects or comorbid conditions. Despite these limitations, the findings provide valuable insights into the pathophysiology of EDS in SLE and suggest avenues for further research.

				hypothalamic lesions and highlight the heterogeneity of underlying mechanisms.	
Tan et al. (2013)	The study addresses the significant question of how exercise and diet interventions impact obesity-related sleep disorders, while also exploring the role of gut microbiota and hormone levels. By investigating these factors, the study offers valuable insights into alternative approaches for managing chronic sleep disorders.	The study's methodology is robust, involving a diverse sample of Finnish men, randomized into intervention and control groups. It employs objective and subjective sleep measurements, along with assessments of various secondary outcomes, providing a comprehensive evaluation of the intervention's effects.	The study collects a wide range of data, including sleep measurements, anthropometry, gut microbiota composition, and hormone levels. These data will be analyzed using appropriate statistical methods to identify relationships between lifestyle interventions and sleep quality. Integrating objective and subjective measures enhances the credibility of the findings.	The study anticipates demonstrating improvements in sleep quality post-intervention, supported by both subjective and objective measures. It aims to establish connections between changes in sleep quality and specific biological markers, offering valuable insights into the mechanisms underlying lifestyle interventions for sleep disorders.	Challenges may include participant recruitment and retention, especially for the intervention groups over a six-month period. The study's focus on Finnish men may limit generalizability. Additionally, while the study investigates multiple factors related to sleep quality, it may not capture all variables. Addressing these limitations through careful participant selection and comprehensive analysis will enhance the validity of the findings.

Tanaka et al. (2014)	The research question aims to investigate immunological alterations in narcolepsy patients, specifically focusing on cytokines, which is relevant to understanding the pathophysiology of the condition.	The methodology is well-described, with the selection of narcolepsy patients with cataplexy and healthy controls matched for age, sex, and body mass index. The use of Bioplex Pro Human Cytokine 17-Plex Assays for measuring plasma cytokines enhances the reliability of the results.	The data analysis appears robust, with statistical comparisons made between the narcolepsy group and healthy controls to identify significant differences in plasma cytokine levels. Additionally, correlations between cytokine levels and disease duration are examined, providing further insights into the relationship between cytokines and narcolepsy progression.	The results indicate elevations of IL-6, IL-8, G-CSF, and tumor necrosis factor-alpha in the narcolepsy group compared to healthy controls, with G-CSF levels significantly correlated with disease duration in narcolepsy patients. These findings suggest a potential role for these cytokines in the pathophysiology of narcolepsy, particularly IL-8 and G-CSF in relation to neutrophil activation and respiratory diseases.	One limitation is the relatively small sample size, which may limit the generalizability of the findings. Additionally, while the study identifies correlations between cytokine levels and disease duration, it does not establish causality. Further research with larger sample sizes and longitudinal studies could provide more comprehensive insights into the role of cytokines in narcolepsy. Moreover, considering other factors such as medication use or comorbidities could enhance the interpretation of the results.
Tasali et al. (2022)					The study acknowledges several limitations, including potential biases related

The research question is clear and relevant, aiming to investigate whether extending sleep duration can mitigate the risk of obesity among adults with overweight who habitually curtail their sleep duration. This addresses an important gap in understanding the relationship between sleep duration and obesity risk and has implications for obesity prevention and weight loss strategies.

The methodology is well-designed, employing a single-center, randomized clinical trial over a substantial period (6 years) to assess the effects of a sleep extension intervention. The inclusion criteria are clearly defined, and objective measures, including doubly labeled water method for total energy expenditure and dual-energy x-ray absorptiometry for body composition, enhance the rigor of the study. However, potential confounders and blinding procedures could be further addressed to strengthen the methodology.

The data analysis appears comprehensive, utilizing intention-to-treat analysis and objective measures to assess changes in sleep duration, energy intake, energy expenditure, and body weight. Statistical comparisons between the sleep extension and control groups are appropriately conducted, and effect sizes and confidence intervals are provided. However, additional details on statistical methods and adjustments for potential confounders could enhance the transparency of the analysis.

The results demonstrate that sleep extension led to a significant increase in sleep duration and a significant decrease in energy intake among adults with overweight. This resulted in a negative energy balance and subsequent weight reduction in the sleep extension group compared to the control group. The findings support the hypothesis that improving and maintaining healthy sleep duration may be beneficial for obesity prevention and weight loss efforts.

to self-reported dietary intake, the relatively small sample size, and the lack of long-term follow-up beyond the intervention period. Additionally, while efforts were made to objectively assess energy intake and expenditure, other factors influencing weight regulation, such as physical activity levels and dietary composition, were not fully accounted for. Addressing these limitations would strengthen the validity and applicability of the findings.

Taylor-Gjevre et al. (2013)	The study effectively addresses the relationship between rheumatic diseases and sleep apnoea, particularly focusing on RA. It aims to determine the prevalence, risk factors, and potential implications of co-existing sleep apnoea in rheumatic disease patients.	The methodology appears robust, employing a combination of literature review, observational studies, and PSG assessments to investigate the association between rheumatic diseases and sleep apnoea. However, more details on the selection criteria for participants and control groups could enhance transparency.	The data analysis seems comprehensive, utilizing PSG recordings, screening questionnaires, and objective sleep assessments to evaluate the prevalence and severity of sleep apnoea in rheumatic disease patients. However, details on statistical methods used for data analysis would strengthen the study's rigor.	The results provide valuable insights into the prevalence of sleep apnoea in rheumatic diseases, with specific emphasis on RA. They highlight the significant association between RA and sleep apnoea, as well as potential implications for disease management and treatment outcomes. However, more specific quantitative results and effect sizes could enhance clarity.	While the study presents compelling findings, several limitations should be noted. These include potential selection bias in participant recruitment, reliance on self-reported symptoms in some cases, and the lack of longitudinal follow-up to assess treatment outcomes and disease progression. Additionally, the generalizability of findings to other rheumatic diseases beyond RA may be limited. Further research with larger sample sizes and more diverse populations could address these limitations.
Tench et al. (2001)	The research question is clearly defined: to measure aerobic	The methodology involves recruiting 93 patients with SLE and 41	The data analysis appears appropriate for the study objectives,	The results are presented clearly, demonstrating	The study acknowledges several limitations, including

fitness, muscle strength, fatigue, and physical disability in patients with systemic lupus erythematosus (SLE) compared to sedentary controls. The question addresses an important gap in understanding the physical and symptomatic manifestations of SLE and has significant implications for the management and treatment of the disease.

sedentary controls into the study and assessing various physical and symptomatic measures including aerobic fitness, muscle strength, fatigue, mood, sleep, and functional incapacity. Aerobic fitness was assessed through a treadmill-walking test, while muscle strength was measured using voluntary isometric quadriceps contraction. Symptomatic measures were obtained through self-report questionnaires. The study design allows for a comprehensive evaluation of physical and symptomatic manifestations in SLE patients compared to controls. However, potential sources of bias such as selection bias and confounding variables are not fully addressed, and the lack of blinding may

utilizing appropriate statistical tests (e.g., t-tests, linear regression models) to compare physical and symptomatic measures between SLE patients and controls and assess correlations between variables. Statistical significance levels are reported, aiding in the interpretation of results. However, more information on potential confounders and adjustment techniques would provide a deeper understanding of the findings.

significant differences between SLE patients and sedentary controls in aerobic fitness, exercise capacity, muscle strength, lung function, fatigue, mood, sleep quality, and functional incapacity. The study highlights the physical and symptomatic burden experienced by SLE patients compared to controls. Correlation analysis suggests relationships between physical disability and aerobic fitness, fatigue, body mass index, and depression, as well as between fatigue and depression. The inclusion of statistical significance levels enhances the reliability of the findings. However, further exploration of potential moderators and subgroup analyses would provide

potential sources of bias such as selection bias and the lack of blinding. The small sample size of sedentary controls may limit the generalizability of findings, and the cross-sectional design limits causal inference. Additionally, the study does not address potential confounders comprehensively, and the lack of objective measures for some variables (e.g., mood, sleep quality) may introduce bias. Further discussion on these limitations and their implications for interpretation would strengthen the discussion section.

		introduce bias in outcome assessment. Further details on participant recruitment and data collection procedures would enhance transparency.		additional insights.	
Theander et al. (2010)	The research question focuses on identifying treatable causes of fatigue in primary Sjögren's syndrome (pSS) patients. It is clearly defined and relevant to the clinical management of the condition, aiming to improve quality of life for patients.	The methodology appears robust, likely involving observational and possibly interventional approaches based on the description. The use of combined evaluation instruments and statistical analysis suggests a comprehensive approach.	Data analysis is likely sound, given the acknowledgment of a biostatistician's involvement. This indicates appropriate handling of statistical methods to interpret findings regarding subjective distress factors like sicca, pain, and fatigue.	The results seem informative, emphasizing that fatigue, musculoskeletal pain, and psychological factors significantly contribute to fatigue in pSS. Various treatment modalities are mentioned, suggesting a practical approach to managing these symptoms.	Possible limitations could include sample size, potential biases in self-reported outcomes, and the scope of treatments evaluated. These factors could influence the generalizability and applicability of the findings to broader pSS populations.
Tibuakuu et al. (2017)	The research question is well-defined, aiming to identify sensitive biomarkers of tobacco-induced subclinical cardiovascular damage by examining the associations between	The study utilizes a cross-sectional design with a large sample size (2,702 participants) from the GENOA study, enhancing the statistical power and generalizability of the	Data analysis employs appropriate statistical techniques, including generalized estimating equations (GEE) to model biomarkers as geometric mean ratios. Adjustments are made	The results are clearly presented, demonstrating significant associations between smoking and biomarkers of inflammation, particularly high	Although the study provides valuable insights, limitations include its cross-sectional design, which precludes establishing causality and temporality.

	smoking and 17 biomarkers of inflammation. This addresses a significant gap in understanding the cardiovascular effects of smoking and informs the study and regulation of emerging tobacco products.	findings. Smoking exposure is comprehensively assessed, including smoking status, intensity, burden, and time since quitting. Biomarkers of inflammation are measured using validated methods, strengthening the reliability of the methodology.	for potential confounders, enhancing the validity of the results. Significance thresholds are adjusted for multiple comparisons using the Bonferroni method, reducing the risk of type I errors.	sensitivity C-reactive protein. Current smokers exhibit significantly higher levels of hsCRP compared to never smokers, with each pack-year of smoking associated with a 0.4% increase in hsCRP levels. Moreover, each 5-year lapsed since quitting is associated with a 4% decrease in hsCRP levels.	Additionally, reliance on self-reported smoking status and pack-years may introduce recall bias. Further research, including longitudinal studies, is needed to confirm these findings and explore potential mechanisms underlying the observed associations.
Toor et al. (2021)	The research question is focused on exploring the therapeutic potential of orexin therapies across various biological functions and medical conditions. It aims to investigate how orexin modulation can impact the sleep-wake cycle, cognitive functioning, and other physiological processes. The question is broad but clearly defined, addressing an	The methodology likely involves reviewing existing literature and preliminary studies on orexin modulation and its therapeutic implications. This may include preclinical and clinical studies investigating the effects of orexin agonists and antagonists on sleep, cognition, and other biological functions. The methodology may also involve evaluating the	The data analysis likely involves synthesizing information from various sources, including preclinical studies, clinical trials, and mechanistic research on orexin modulation. Analytical methods may include systematic review and meta-analysis techniques to summarize findings across studies and assess the overall	The results of the study are expected to provide insights into the diverse therapeutic potential of orexin therapies across different biological functions and medical conditions. This may include improvements in sleep quality, cognitive functioning, daytime performance, and potentially the management of conditions such as	While the study presents valuable insights into the therapeutic potential of orexin therapies, it may have limitations inherent to literature reviews and synthesis studies. These may include biases introduced by selective reporting in the literature, heterogeneity across studies in methodologies and

	area of significant interest and potential clinical relevance. The study seeks to elucidate the scope of orexin therapies' therapeutic benefits, which is an important aspect for further research and clinical development.	safety, efficacy, and mechanisms of action of orexin-based therapies. Overall, the methodology is appropriate for synthesizing and analyzing existing evidence on orexin therapies' therapeutic potential.	efficacy and safety profile of orexin therapies. Additionally, qualitative analysis may be conducted to identify key themes and trends in the literature regarding orexin modulation and its therapeutic effects.	narcolepsy, anorexia nervosa, and neurodegenerative diseases. The results highlight the promising avenues for further research and clinical development of orexin-based therapies.	patient populations, and the lack of direct clinical evidence for some therapeutic applications. Additionally, the study may not fully capture emerging research in the field or potential future developments in orexin-based therapies.
Trotti et al. (2012)	The study aims to assess the association between periodic leg movements (PLMs) and systemic inflammation in restless legs syndrome (RLS) patients to further understand their cardiovascular risk. This addresses a gap in the literature and has clinical	The study included 137 RLS patients and measured PLMs while they were unmedicated for RLS. Banked plasma was assayed for high sensitivity C-reactive protein (CRP), interleukin-6 (IL-6), and tumor necrosis factor-alpha (TNF-alpha). Statistical analysis involved correlational	The data analysis indicates that PLMs were modestly correlated with logCRP, but not with TNF-alpha or IL-6. Patients with at least 45 PLMs per hour had significantly higher odds of elevated CRP levels compared to those with fewer PLMs. After adjusting for	The study findings suggest that PLMs are associated with increased inflammation in RLS patients, as evidenced by higher CRP levels. Patients with a higher PLM index had more than triple the odds of elevated CRP levels compared to those with fewer PLMs. These	One limitation is the lack of a control group, which limits the ability to compare PLM and inflammation levels between RLS patients and non-RLS individuals. Additionally, the observational nature of the study precludes causal inference, and residual confounding

	relevance given the potential implications for cardiovascular health in RLS patients.	analyses and logistic regression to assess the relationship between PLMs and inflammation while adjusting for potential confounders. The methodology appears robust and appropriate for the research objectives.	multiple potential confounders, the association between PLMs and elevated CRP remained significant. The statistical analysis is comprehensive and accounts for potential confounders, enhancing the reliability of the results.	results underscore the potential cardiovascular implications of PLMs in RLS patients and warrant further investigation into the relationship between PLMs and inflammation.	may have influenced the results despite adjustments. Further research with longitudinal designs and control groups is needed to confirm the findings and elucidate underlying mechanisms.
Urashima et al. (2020)	The research question is well-defined: to determine the association between disease activity of rheumatic diseases and sleep-related problems, including the prevalence of restless legs syndrome (RLS) among Japanese outpatients. The question is relevant and specific to the study population.	Strengths: The study used a cross-sectional design, which is appropriate for examining associations between variables. It included a well-defined patient selection process based on clear eligibility criteria, ensuring consistency and relevance to rheumatic diseases. Weaknesses: There's potential for selection bias as the study only included patients from a single outpatient clinic, which may limit	The data analysis utilized appropriate statistical methods including non-parametric tests like Wilcoxon rank sum test and Fisher's exact test, suitable for the non-normal distribution of some variables. CART analysis was used effectively to identify predictors of RLS, enhancing understanding of the factors associated with this condition in the context of rheumatic diseases.	Strengths: Clear presentation of results with detailed demographic and clinical characteristics of patients, including prevalence rates of RLS and insomnia symptoms. Effective use of figures (e.g., histograms) to visually represent data. Weaknesses: Limited discussion on the potential mechanisms underlying the observed associations, such as the interaction between disease	The study acknowledged several limitations, such as the single-center design, which limits generalizability, and the exclusion of patients with comorbidities, potentially affecting the representativeness of the sample. Additionally, the reliance on self-reported measures for sleep assessment could introduce recall bias. The study also did not explore longitudinal

		generalizability. Additionally, the exclusion criteria might have excluded patients with relevant comorbidities, impacting the broader applicability of findings.		activity markers and sleep disturbances.	associations between disease activity and sleep disturbances, which could provide insights into causality.
Uslu et al. (2021)	The study aims to investigate the role of inflammatory processes in the pathogenesis of primary restless legs syndrome (RLS) by comparing circulating levels of hsCRP, IL-1β, IL-6, and TNF-α in patients with RLS and healthy controls. This addresses a gap in the literature and has implications for understanding the underlying mechanisms of RLS.	The study prospectively included 29 patients with primary RLS and 65 age-sex matched healthy controls. RLS diagnosis was established using international guidelines, and severity was evaluated using the IRLSSG Severity Scale. Plasma levels of inflammatory markers were measured in all participants. The methodology appears rigorous and appropriate for the research objectives.	The data analysis indicates statistically significantly higher levels of IL-1β, IL-6, and TNF-α in patients with RLS compared to healthy controls, while hsCRP levels did not differ between groups. Additionally, IL-6 levels were significantly higher in patients with severe RLS compared to those with mild or moderate RLS. The statistical analysis appears appropriate, with comparisons made using relevant tests and adjustments for multiple comparisons.	The study findings suggest a potential role of inflammation in the pathogenesis of primary RLS, as evidenced by higher circulating levels of inflammatory cytokines in patients with RLS compared to healthy controls. Moreover, IL-6 levels were particularly elevated in patients with severe RLS. These results contribute to our understanding of RLS pathophysiology and highlight inflammation as a potential therapeutic target.	One limitation is the relatively small sample size, which may limit the generalizability of the findings. Additionally, the observational nature of the study precludes causal inference, and potential confounding factors not addressed in the analysis could influence the results. Further research with larger cohorts and longitudinal designs is needed to confirm the findings and elucidate underlying mechanisms.

Vadell et al. (2020)	The research question addresses an important issue: whether an anti-inflammatory diet can reduce disease activity in patients with rheumatoid arthritis (RA). The study aims to investigate the efficacy of a specific dietary intervention in managing RA symptoms, which is relevant given the reported effects of certain foods on RA symptoms. The question is clearly articulated and has practical implications for dietary recommendations in RA management.	The study employed a single-blinded crossover trial design involving 50 patients with RA who were randomly assigned to either an intervention diet containing anti-inflammatory foods or a control diet similar to the general dietary intake in Sweden, for 10 weeks each. Food equivalent to approximately 50% of energy requirements was delivered weekly to participants' homes, and they were encouraged to consume similar foods for remaining meals. Disease activity was assessed using the Disease Activity Score in 28 joints-Erythrocyte Sedimentation Rate (DAS28-ESR) as the primary outcome measure. The methodology appears robust and well-designed to evaluate the effect of the anti-inflammatory diet on	The data analysis involved linear mixed ANCOVA models and Wilcoxon's Signed Rank test to compare changes in disease activity between the intervention and control periods. The primary outcome, DAS28-ESR, was analyzed, along with secondary outcomes including components of DAS28-ESR. The statistical methods used appear appropriate for the study design, and the results were analyzed rigorously.	The main analysis did not show a significant difference in DAS28-ESR between the intervention and control periods. However, unadjusted analyses suggested a significant decrease in DAS28-ESR during the intervention period, with a significantly lower score after the intervention compared to the control period in participants who completed both periods. This indicates potential positive effects of the anti-inflammatory diet on disease activity in patients with RA, although further studies are needed to confirm	While the study provides valuable insights into the potential benefits of an anti-inflammatory diet in RA management, several limitations should be considered. These include the relatively small sample size, which may limit the generalizability of the findings, and the short duration of the intervention period. Additionally, the study relied on self-reported dietary intake and did not include objective measures of dietary adherence. Further research with larger sample sizes and longer follow-up periods is needed to validate the efficacy of the anti-inflammatory diet in RA management.

		disease activity in patients with RA.		these findings.	
Vahid et al. (2020)	The study addresses an important research question concerning the relationship between dietary inflammation and obesity/overweight in adolescent boys, highlighting the potential role of diet in the development of obesity-related chronic diseases. By comparing the dietary inflammatory index between normal weight and overweight adolescents, the study	The methodology involves recruiting a substantial sample size of adolescent boys from two schools in Tehran, Iran, and employing validated instruments to assess weight, body composition, and dietary inflammatory index. The use of a semi-quantitative Food Frequency Questionnaire enhances the comprehensiveness of dietary assessment. However, the study could benefit from	Data analysis includes statistical modeling to examine the association between DII® and obesity/overweight, adjusting for potential confounders. The results demonstrate a positive association between DII® and obesity, with subjects having higher DII® scores showing increased odds of obesity and overweight. The use of multivariable adjustment strengthens	The results highlight the significant association between dietary-induced inflammation, as measured by DII®, and obesity/overweight in adolescent boys. The findings underscore the importance of dietary factors in shaping obesity risk during adolescence, suggesting that diets with lower DII® scores, rich in fruits, vegetables, fiber, flavonoids, and certain	Limitations of the study include potential confounding factors that were not fully accounted for in the analysis, such as physical activity levels, socioeconomic status, and genetic predisposition to obesity. Additionally, the cross-sectional design precludes the establishment of causality and temporality between dietary inflammation and obesity,

	aims to elucidate the association between dietary-induced inflammation and obesity during adolescence. Overall, the research question is relevant and contributes to our understanding of the dietary factors influencing obesity in this population.	additional measures to control for potential confounding factors, such as physical activity levels, socioeconomic status, and family history of obesity. Nevertheless, the methodology provides a solid foundation for investigating the association between dietary inflammation and obesity in adolescent boys.	the robustness of the findings by controlling for potential confounding variables. However, the study could benefit from additional analyses to explore the dose-response relationship between DII® and obesity and assess the contribution of specific dietary components to inflammatory status.	minerals, may support healthy weight management. The results provide valuable insights into potential dietary interventions for preventing and managing obesity-related chronic diseases in this population.	highlighting the need for longitudinal studies to confirm the observed associations. Moreover, the reliance on self-reported dietary data may introduce recall bias and affect the accuracy of dietary assessment. Despite these limitations, the study provides important preliminary evidence regarding the role of dietary-induced inflammation in adolescent obesity.
Varma et al. (2007)	The research question is well-defined and significant, aiming to examine the association of visfatin (VF) with insulin sensitivity, intramyocellular lipids (IMCL), and inflammation in humans. The study seeks to understand the differential expression of VF in subcutaneous	The methodology is robust and appropriate for the research question. The study utilizes paired samples of VAT and SAT, as well as muscle tissue biopsies from well-characterized subjects with varying glucose tolerance, BMI, and insulin sensitivity. This allows for a comprehensive analysis	The data analysis is thorough, examining the relationships between VF mRNA expression in different adipose tissue depots and various metabolic parameters such as BMI, insulin sensitivity, IMCL, and markers of inflammation. The statistical methods used are appropriate	The results indicate that VF mRNA expression differs between SAT and VAT, with SAT VF being positively associated with insulin sensitivity and negatively with IMCL and inflammatory markers. In contrast, VAT VF mRNA is positively associated with BMI.	Limitations include the small sample size and potential lack of generalizability to a broader population. The cross-sectional nature of the baseline data limits the ability to infer causality. Additionally, the study does not explore the mechanistic pathways underlying

	adipose tissue (SAT) and visceral adipose tissue (VAT), and how these relate to metabolic health markers, providing valuable insights into the role of VF in metabolic regulation and its potential impact on conditions like insulin resistance and inflammation.	of VF mRNA expression across different tissues and its correlation with metabolic health markers. The use of pioglitazone or metformin treatment in impaired glucose tolerance subjects adds a valuable interventional aspect to the study. However, the sample size and generalizability to a broader population are potential limitations.	for determining significant associations and independent relationships. The study effectively highlights the opposing regulation of VF mRNA in SAT and VAT with respect to BMI and its independent association with insulin sensitivity. The analysis also explores the impact of insulin sensitizer treatments on VF expression, adding depth to the findings.	The study also finds that insulin sensitizer treatments (pioglitazone) do not significantly alter SAT VF mRNA levels despite improving insulin sensitivity. These findings suggest that SAT VF is highly expressed in lean, insulin-sensitive individuals and is downregulated in the presence of higher IMCL, lower insulin sensitivity, and increased inflammation, indicating differential roles of VF in SAT and VAT with metabolic implications.	the differential expression and regulation of VF in SAT and VAT. The reliance on biopsy samples may introduce variability, and the study's findings need to be confirmed in larger, more diverse populations and through longitudinal studies to understand the long-term implications of VF expression on metabolic health.
Vgontzas et al. (1997)	The research question is clear and focused on investigating the role of TNF alpha, IL-1beta, and IL-6 in excessive daytime sleepiness (EDS) and fatigue in sleep disorder patients.	The methodology appears appropriate with the measurement of plasma levels of TNF alpha, IL-1beta, and IL-6 in different groups of patients and controls. However, the sample	The data analysis seems appropriate, with statistical comparisons made between the different groups to assess differences in plasma cytokine levels. The	The results indicate that TNF alpha is significantly elevated in sleep apneics and narcoleptics compared to normal controls, while IL-6 is markedly elevated in sleep	One limitation is the small sample size, which may limit the generalizability of the findings. Additionally, the study only measured morning plasma levels,

		size is relatively small, which could affect the generalizability of the results.	primary factors influencing TNF alpha and IL-6 levels are identified.	apneics. However, IL-1beta levels did not show significant differences between patient groups and controls.	potentially missing fluctuations throughout the day. Further research with larger sample sizes and comprehensive cytokine monitoring could provide more robust insights. Additionally, other factors influencing cytokine levels, such as comorbidities or medication use, should be considered in future studies.
Vgontzas et al. (1999)	The research question addresses the relation between plasma IL-6 levels and sleep parameters, as well as the effects of sleep deprivation on the nyctohemeral pattern of IL-6 secretion. This is relevant for understanding the physiological mechanisms linking sleep and inflammation.	The methodology involves serial measurements of plasma IL-6 and polysomnography in healthy young male volunteers under baseline and sleep-deprived conditions. This design allows for the examination of IL-6 secretion patterns in relation to sleep quantity and quality. However, the sample size is small,	The data analysis appears thorough, with statistical analyses revealing significant correlations between sleep parameters and IL-6 secretion patterns. The comparison of IL-6 levels before and after sleep deprivation provides valuable insights into the effects of sleep disruption on inflammation.	The results demonstrate a biphasic circadian pattern of IL-6 secretion, with nadirs at 0800 and 2100 and zeniths at 1900 and 0500. Sleep parameters, such as the amount and depth of sleep at baseline, are negatively correlated with daytime IL-6 secretion and post-deprivation increase, respectively. Sleep deprivation alters	One limitation is the small sample size of only eight healthy young male volunteers, which may limit the generalizability of the findings and raise questions about applicability to broader populations, including females and older individuals. Additionally, the study's focus on acute

		limiting the generalizability of the findings.		the temporal pattern of IL-6 secretion, leading to daytime oversecretion and nighttime under-secretion.	sleep deprivation may not fully capture the long-term effects of chronic sleep disturbances on IL-6 secretion and inflammation. Future research with larger and more diverse samples, as well as longitudinal designs, could provide further insights into the complex relationship between sleep and inflammation.
Vina et al. (2013)	The research question is well-defined, focusing on the relationship between psychosocial variables (depression, anxiety) and sleep quality in patients with systemic lupus erythematosus (SLE). It also aims to identify specific sleep disturbances prevalent in SLE patients and the contributing factors. This is a relevant and	The study employs a cross-sectional design, utilizing the MOS Sleep Scale to assess sleep disturbances and psychosocial variables like depression (measured by BDI) and anxiety (measured by STAI). Polysomnography is also mentioned for measuring respiratory abnormalities during sleep. While the methodology is	The data analysis appears robust, with depression and anxiety being moderately and significantly correlated with various MOS Sleep subscale scores. The use of multiple regression analysis to control for confounding factors is appropriate. However, the text notes the difficulty in determining the directionality of the	The results highlight the significant role of depression and anxiety in mediating sleep disturbances in SLE patients. Depression is found to be independently associated with sleep adequacy and has a strong correlation with various sleep subscale scores. Anxiety is also correlated with certain sleep disturbances like	Internal Validity: The cross-sectional design limits the ability to infer causation, meaning the study can identify correlations but cannot establish cause-and-effect relationships between psychosocial factors and sleep disturbances. External Validity: The sample is drawn from a single academic, urban

significant question given the known impacts of SLE on sleep and overall quality of life.	appropriate for identifying correlations, the cross-sectional nature limits the ability to establish causation. The recruitment of participants from a single academic institution might introduce bias.	associations due to the study's design, which is a limitation.	snoring and daytime somnolence. These findings are consistent with previous research and add to the understanding of sleep issues in SLE.	institution, which might not be representative of the broader SLE population. Only non-hospitalized patients were included, which may not capture the full range of SLE severity and its impact on sleep. Methodological Considerations: The study lacks gender-, age-, and race/ethnicity-stratified MOS Sleep scores for the general population, limiting the ability to make comparative analyses. Additionally, the reliance on self-reported measures may introduce reporting bias, and there is no mention of control for potential confounders such as medication use, comorbid conditions, or lifestyle factors.

Wali et al. (2021)	The research question aims to evaluate the association between recently diagnosed obstructive sleep apnea (OSA) and inflammatory markers (TNF-α and IL-6), as well as the effect of short-term continuous positive airway pressure (CPAP) therapy on these markers. The question is clearly defined and relevant to the study's objectives.	The methodology employs a prospective, open-label, controlled trial design, which is appropriate for investigating the association between OSA and inflammatory markers and assessing the impact of CPAP therapy. Patient groups are well-defined based on PSG results, and repeated measures ANOVA and ANCOVA are utilized for data analysis, accounting for within-subject effects and adjusting for covariates. Overall, the methodology is robust and aligns with the study's objectives.	The data analysis effectively compares baseline and post-intervention levels of TNF-α and IL-6 among the three patient groups. Repeated measures ANOVA and ANCOVA are appropriate statistical techniques for examining changes over time and between groups while controlling for covariates. The presentation of statistical results is clear and concise, facilitating interpretation of findings.	The results indicate significantly higher baseline levels of IL-6 and TNF-α in OSA patients compared to non-OSA controls. However, there are no significant changes in these inflammatory markers following 1-month CPAP therapy. The absence of interaction effects between group and time suggests that CPAP therapy did not influence IL-6 and TNF-α levels differently across patient groups. Results are presented logically and support the study's conclusions.	The study acknowledges several limitations, including the relatively short duration of CPAP therapy, which may not have been sufficient to induce changes in inflammatory markers. Additionally, the study does not address potential confounding factors such as comorbidities or medication use that could influence inflammatory marker levels. While these limitations are acknowledged, addressing them could strengthen the study's findings and implications.
Wang et al. (2015)		The methodology section outlines the systematic search strategy and inclusion criteria for identifying relevant studies. PubMed, OVID,	The data analysis involves estimating pooled relative risks (RRs) of adverse symptoms associated with anti-TNF-α	The results section presents findings from the meta-analysis of 23 RCTs involving 7325 patients with IBD. Adverse symptoms	The study acknowledges several limitations, including the reliance on RCTs with placebo arms, which may not fully

The research question is clearly defined and aims to assess the duration-response relationship between anti-TNF-α agents and the risk of adverse symptoms in inflammatory bowel disease (IBD) patients. The study seeks to fill a gap in the literature by conducting a meta-analysis of randomized controlled trials (RCTs) comparing anti-TNF-α therapy with placebo in adults with IBD. Specifically, the study investigates the occurrence of adverse symptoms such as headache, nausea/vomiting, abdominal pain, fever, arthralgia, and fatigue. By addressing this and Cochrane Library databases were searched up to January 2015, and RCTs comparing anti-TNF-α therapy with placebo in adults with IBD were included. The meta-analysis estimated pooled relative risks (RRs) of adverse symptoms for anti-TNF-α therapy and examined both non-linear and linear duration-response relationships between therapy duration and significant adverse symptoms. While the methodology provides sufficient details on study selection and data analysis, it lacks information on potential sources of bias and quality assessment of included studies. Nonetheless, the methodology is appropriate for addressing the research question and conducting a meta-analysis of RCTs therapy and examining the duration-response relationship between therapy duration and fatigue. Adverse symptoms such as headache, nausea/vomiting, abdominal pain, fever, and arthralgia showed no significant relationship with anti-TNF-α therapy. However, fatigue was significantly associated with anti-TNF-α therapy, particularly in trials with long therapy duration (>30 weeks) and without azathioprine (AZA) combination. Subgroup analysis and linear regression were used to assess the relationship between therapy duration and fatigue risk. The data analysis provides comprehensive insights into the association between anti-TNF-α such as headache, nausea/vomiting, abdominal pain, fever, and arthralgia showed no significant relationship with anti-TNF-α therapy. However, fatigue was significantly associated with anti-TNF-α therapy, with long therapy duration and absence of AZA combination identified as risk factors. Subgroup analysis and linear regression revealed a linear duration-response relationship between therapy duration and fatigue risk in trials without AZA combination. The results are clearly presented and supported by appropriate statistical analyses, providing valuable insights into the safety profile of anti-TNF-α therapy in capture real-world treatment scenarios. Additionally, the inclusion of studies up to January 2015 may limit the generalizability of findings to more recent anti-TNF-α agents and treatment practices. Furthermore, the study does not address potential sources of bias or quality assessment of included studies, which could affect the reliability of results. While subgroup analysis and linear regression were used to explore duration-response relationships, residual confounding and unmeasured variables may influence the observed associations. Despite these limitations, the study provides valuable insights into the safety

	question, the study provides valuable insights into the safety profile of anti-TNF-α therapy in IBD patients and identifies potential risk factors associated with treatment duration. Overall, the research question is relevant, well-formulated, and addresses an important aspect of IBD management.	in IBD patients receiving anti-TNF-α therapy.	therapy and adverse symptoms, as well as the impact of treatment duration on fatigue occurrence. Overall, the data analysis is robust and supports the study findings effectively.	IBD patients. Overall, the results contribute to a better understanding of the relationship between treatment duration and adverse symptoms in IBD management.	profile of anti-TNF-α therapy in IBD patients and identifies areas for further research, such as the impact of newer agents and treatment strategies on adverse symptoms. Future studies should address these limitations to enhance the robustness and applicability of findings.
Wang et al. (2020)	The research question addresses the association between ankylosing spondylitis (AS) and sleep apnea–hypopnea syndrome (SAHS), which is a novel and relatively unexplored topic in the literature. By presenting a case study of SAHS in a patient	The methodology involves a case study approach, where a single patient with AS and SAHS is presented, and the treatment outcomes are described. The interventions implemented for both AS and SAHS are outlined, including pharmacological	The abstract does not provide specific details on data analysis, as it primarily focuses on describing the patient's clinical presentation, diagnosis, interventions, and outcomes. However, data analysis likely involved assessing changes in symptoms	The results section summarizes the outcomes of the interventions implemented for AS and SAHS. Six months after the initiation of treatment, significant improvements in the clinical manifestations of both AS and SAHS are reported. This	The abstract does not explicitly discuss limitations of the study. However, potential limitations include the lack of generalizability due to the case study design, the absence of control group comparisons, and the reliance on subjective reports for

	with AS and discussing the possible underlying mechanisms, the study aims to increase awareness of this association and highlight the importance of assessing SAHS symptoms in patients with AS. Overall, the research question is relevant and fills a knowledge gap in the field of rheumatology and sleep medicine.	treatment and nasal continuous positive airway pressure (CPAP) therapy. While the case study design provides valuable clinical insights and allows for in-depth examination of the patient's condition, it has limitations in terms of generalizability and establishing causality. Future research could benefit from larger-scale studies to confirm the association between AS and SAHS and explore underlying mechanisms through experimental or observational research designs.	and disease severity following the implemented treatments for AS and SAHS. The evaluation of treatment outcomes may have included clinical assessments, laboratory tests, and subjective reports from the patient. While the case study design limits the scope of quantitative data analysis, it allows for a detailed examination of individual patient responses to treatment.	includes improvements in symptoms and disease severity, indicating a positive response to the prescribed medications and CPAP therapy. The results support the hypothesis that patients with AS may be prone to SAHS due to various factors, including airway compression, central depression of respiration, and abnormal inflammatory responses. Overall, the results highlight the efficacy of the treatment approach and the importance of considering SAHS symptoms in patients with AS.	assessing treatment outcomes. Additionally, the underlying mechanisms proposed for the association between AS and SAHS are speculative and require further investigation through experimental research. Future studies should address these limitations by employing larger sample sizes, control groups, objective outcome measures, and mechanistic studies to provide robust evidence on the relationship between AS and SAHS.
Ward et al. (2018)	The research question addresses the feasibility of a relaxation-based	The methodology involves recruiting participants from a	Data analysis involves assessing feasibility indicators such as	The results indicate that the relaxation-based yoga	Several limitations should be considered, including the

	yoga intervention for rheumatoid arthritis (RA) and is clearly defined. The study aims to determine feasibility based on various criteria such as recruitment rates, retention, protocol adherence, participant satisfaction, adverse events, and secondary outcomes related to physical and psychosocial aspects. The question aligns with the study's objectives and provides valuable insights into the potential benefits of yoga for RA management.	hospital database and randomizing them into either a yoga intervention group or a usual care control group. Feasibility is assessed based on predefined criteria, and physical and psychosocial outcomes are measured using self-reported questionnaires at multiple time points. The study design appears appropriate for evaluating the feasibility and safety of the relaxation-based yoga intervention in participants with RA, following Delphi recommendations for yoga interventions for musculoskeletal conditions.	recruitment rates, retention, protocol adherence, participant satisfaction, and adverse events, as well as analyzing secondary outcomes related to physical and psychosocial aspects using self-reported questionnaires. The presentation of results appears comprehensive, with relevant statistical measures provided to support the findings.	intervention was feasible and safe for participants with RA-related pain and functional disability. High retention rates, protocol adherence, and participant satisfaction were observed, with no serious adverse events related to the intervention. However, there were no significant group effects of yoga compared with usual care on secondary outcomes. The results provide valuable insights into the feasibility and safety of yoga for RA management.	relatively small sample size and the use of self-reported questionnaires for outcome assessment, which may introduce bias. Additionally, the lack of significant group effects on secondary outcomes suggests that further research is needed to explore the efficacy of yoga for RA management. Despite these limitations, the study provides a valuable framework for larger intervention studies and supports further exploration of yoga as a complex intervention for RA.
Weinberger et al. (2015)	The research question is clearly defined, aiming to investigate the impact of tumor	The methodology involves a prospective study in which 36 patients with treatment-	The data analysis likely involves comparing sleep parameters between infliximab-	The results indicate that TNF blockade with infliximab does not lead to significant	The study may have several limitations, including the relatively small sample

	necrosis factor (TNF) blockade on sleep parameters in depressed patients, particularly those with increased inflammation. This addresses an important gap in the literature, considering the association between inflammation and sleep disturbances in depression. The question is comprehensive, exploring the potential therapeutic effects of TNF blockade on sleep alterations in depressed patients with increased inflammation.	resistant major depression receive either the TNF antagonist infliximab or placebo, with sleep parameters measured using polysomnography at baseline and 2 weeks after treatment. Markers of inflammation, including c-reactive protein (CRP) and TNF, are also assessed alongside depression severity using the Hamilton Depression Rating Scale. The study design allows for the evaluation of sleep changes following TNF blockade in relation to inflammation levels.	treated and placebo-treated groups, particularly focusing on changes in sleep parameters from baseline to week 8. Subgroup analyses may also be conducted based on inflammation levels, comparing sleep changes between patients with high and low inflammation. Correlation analyses between changes in inflammatory markers and sleep parameters may further elucidate the relationship between inflammation and sleep.	changes in overall sleep parameters compared to placebo in depressed patients. However, subgroup analyses reveal that patients with high inflammation levels show improvements in sleep parameters following infliximab treatment, including decreased wake after sleep onset (WASO) and increased sleep efficiency. Correlation analyses further support the association between changes in inflammatory markers and sleep improvements.	size and the focus on patients with treatment-resistant major depression, which may limit the generalizability of the findings. Additionally, the short follow-up period of 2 weeks after treatment may not capture long-term effects of TNF blockade on sleep. Furthermore, other factors influencing sleep, such as medication use and comorbidities, are not fully accounted for in the analysis. Addressing these limitations would strengthen the validity and applicability of the results.
Wu et al. (2016)	The research question	The methodology	Data analysis involves	The results indicate	The study's limitations

is clearly defined, aiming to investigate the potential associations between specific single-nucleotide polymorphisms (SNPs) in the 5-hydroxytryptamine receptor 2A (5-HTR2A) and interleukin-6 (IL-6) genes and obstructive sleep apnea-hypopnea syndrome (OSAHS). The question addresses an important aspect of genetic susceptibility to OSAHS and provides a focused direction for the study.	involves collecting cases and controls for genotyping of specific SNPs (rs6311 in 5-HTR2A and rs1800796 in IL-6) and assessing their association with OSAHS risk. Multivariate unconditional logistic regression analyses are conducted, adjusting for gender and age. The approach is appropriate for investigating genetic associations and controlling for potential confounding variables.	computing odds ratios (OR) and 95% confidence intervals (CI) from logistic regression analyses to evaluate the association between SNPs and OSAHS risk, considering gender and age as covariates. Subgroup analyses are performed to assess genotype and allele distributions in different OSAHS severity groups. The analysis techniques are suitable for examining genetic associations and exploring potential differences based on disease severity and gender.	that genotype and allele frequencies of rs6311 and rs1800796 SNPs are not significantly different between cases and controls overall. However, gender-specific comparisons reveal a protective effect of the ‘C’ allele of rs6311 against OSAHS in males. Additionally, significant differences in genotype distributions of rs6311 and rs1800796 are observed between OSAHS severity groups, suggesting relevance to disease severity.	may include the relatively small sample size of cases and controls, which could affect the statistical power to detect significant associations. Additionally, the study's findings may be influenced by potential confounding factors not accounted for in the analysis. The study's retrospective design and reliance on self-reported data may introduce recall bias and limit the accuracy of genotype-phenotype associations. Further validation in larger, prospective cohorts is needed to confirm the observed associations.

Wu et al. (2019)	The research question addresses a significant gap in knowledge by investigating the effect of physical activity counseling on physical activity levels and its association with fatigue, quality of sleep, and quality of life in women with systemic lupus erythematosus (SLE). It is clear, relevant, and contributes to understanding non-pharmacological interventions for managing SLE symptoms and improving overall well-being.	The study employed a randomized, controlled, single-blind trial design, which is appropriate for assessing the impact of physical activity counseling on SLE patients. Random assignment of participants to intervention and control groups minimizes bias, and blinding reduces the risk of subjective influences on outcome assessments. The intervention involved structured physical activity counseling sessions and follow-ups, enhancing the rigor of the methodology. Overall, the methodology is robust for evaluating the effects of physical activity counseling on various outcomes in SLE women.	Data analysis involved comparing outcomes between the intervention and control groups at baseline, 8 weeks, and 12 weeks using appropriate statistical tests. The study reported significant improvements in daily steps, quality of sleep, vitality, and mental health in the intervention group compared to the control group. Additionally, correlations between changes in physical activity and changes in vitality and mental health were analyzed. The data analysis appears thorough and appropriate for the research objectives.	The results demonstrate that physical activity counseling led to significant improvements in physical activity levels, quality of sleep, vitality, and mental health in SLE women compared to the control group. The findings support the effectiveness of physical activity counseling in enhancing various aspects of well-being in SLE patients. Moreover, the positive correlation between physical activity changes and improvements in vitality and mental health provides valuable insights into the relationship between physical activity and quality of life in SLE.	While the study provides valuable insights, several limitations should be considered. These include the relatively small sample size, which may limit the generalizability of the findings. Additionally, the study's duration of 12 weeks may not capture long-term effects of physical activity counseling on SLE outcomes. Furthermore, potential confounding variables and biases should be addressed to strengthen the validity of the results. Future research with larger sample sizes and longer follow-up periods is warranted to confirm the effectiveness of physical activity counseling in SLE management.

Xia et al. (2021)	The research question is explicitly stated: whether associations exist between insomnia and overlooked inflammatory factors (SAA, TNF-α, GM-CSF, and RANTES) to understand changes in inflammatory factors in CID patients. The question is specific, relevant, and aligns with the study's objectives.	The methodology section describes the sample recruitment process, inclusion and exclusion criteria, data collection methods (including assessment scales and blood sample analysis), and statistical analysis procedures. It demonstrates a systematic approach to participant selection, data collection, and analysis, ensuring rigor and reliability in the study.	The data analysis section employs appropriate statistical methods (t-tests, Mann–Whitney U-tests, Spearman correlation analysis, partial correlation analysis, stepwise linear regression, and ROC curve analysis) to analyze the relationship between inflammatory biomarkers and insomnia severity. The results are presented clearly with statistical values and interpretations, facilitating understanding and interpretation of the findings.	The results section presents findings on demographic and clinical characteristics, serum levels of inflammatory biomarkers, correlations with mood and sleep quality/insomnia severity, and ROC curve analysis results. The tables provide comprehensive data summaries, and the text offers detailed explanations and interpretations of the findings, including comparisons with previous studies and implications of the results. The results are logically organized, addressing each research objective effectively.	The limitations section acknowledges potential shortcomings of the study, including the cross-sectional design, small sample size, limited statistical power, and focus on serum levels of inflammatory factors without tracking changes during treatment or assessing associations with insomnia-related complications. The limitations are transparently presented, indicating areas for future research improvement and caution in interpreting the study findings.

Yoshizawa et al. (	The research question addresses the possible influence of genetics on obstructive sleep apnea syndrome (OSAS) by investigating HLA-A, B, C, and DR antigens in Japanese subjects with OSAS. The question is clearly defined and focuses on exploring the association between specific HLA antigens and OSAS susceptibility.	The methodology involves studying HLA-A, B, C, and DR antigens in 32 Japanese subjects with OSAS and comparing their frequencies with normal controls and the Japanese population. The approach is appropriate for investigating genetic associations with OSAS, and the comparison with population frequencies strengthens the validity of the findings. However, the small sample size limits the study's power and generalizability, and the methodology does not account for potential confounding factors or interactions with other genetic or environmental factors.	Data analysis includes comparing the frequencies of HLA antigens between OSAS patients, normal controls, and the Japanese population. Statistical significance is assessed using appropriate tests, and correction for multiple comparisons is applied ($Pc < 0.035$ and $Pc < 0.007$). The analysis method is suitable for investigating genetic associations and identifying significant differences in HLA antigen frequencies between groups.	The results indicate a markedly increased frequency of HLA-A2 antigen in OSAS patients compared to normal controls and the Japanese population. Similarly, HLA-B39 is found more frequently in OSAS patients than in the Japanese population. However, no significant deviation is observed in the frequencies of HLA-C and DR antigens between OSAS patients and controls. The findings suggest a potential role of specific HLA antigens, particularly HLA-A2 and HLA-B39, in the development of OSAS in Japanese individuals.	The study's limitations include the small sample size, which may limit the generalizability of the findings. Additionally, the lack of adjustment for potential confounding factors or consideration of gene-gene or gene-environment interactions limits the ability to establish causal relationships between HLA antigens and OSAS. Further studies with larger sample sizes and comprehensive analyses are needed to confirm the observed associations and explore the underlying mechanisms contributing to the genetic predisposition to OSAS.

Yottasan et al. (2022)	The research question is well-defined and significant, aiming to determine the prevalence and factors related to sleep impairments in Thai adolescents with systemic lupus erythematosus, and the associations of these impairments with health-related quality of life. This is important given the susceptibility of adolescents with SLE to sleep impairments and the potential impact on their HRQOL. By assessing these factors, the study addresses a crucial aspect of managing SLE in adolescents and improving their overall well-being.	The methodology is appropriate for the research question, employing standardized questionnaires such as the Pittsburgh Sleep Quality Index, Patient Health Questionnaire for Adolescents, and Pediatric Quality of Life Inventory™ 4.0 Core Scales to assess sleep, depression, and HRQOL, respectively. The study includes 57 participants with SLE aged 13-18 years, providing a representative sample for the study population. However, the cross-sectional design limits the ability to establish causality, and the use of self-reported measures may introduce bias.	The data analysis is thorough, identifying significant associations between sleep impairments and factors such as high body mass index and depression scores. The odds ratios provide quantitative measures of these associations, adding rigor to the analysis. Additionally, the comparison of HRQOL scores between good sleepers and poor sleepers across different sub-categories effectively highlights the impact of sleep impairments on emotional functioning. The use of statistical tests to determine significance adds credibility to the findings.	The results indicate that a significant proportion (31.6%) of adolescents with SLE experience sleep impairments, which are associated with obesity and depression. These impairments negatively impact HRQOL, particularly emotional functioning. The study underscores the importance of addressing sleep impairments in adolescents with SLE to improve their overall well-being. The findings provide valuable insights into the factors contributing to sleep impairments and their consequences on HRQOL in this population.	Limitations include the cross-sectional design, which limits the ability to establish causality and temporality of associations. The use of self-reported measures may introduce bias, and the small sample size (57 participants) may affect the generalizability of the findings. Additionally, the study does not explore potential underlying mechanisms linking SLE, sleep impairments, and HRQOL, which could provide further insights. Longitudinal studies with larger, more diverse populations are needed to confirm these findings and explore underlying mechanisms.

Zisapel (2018)	The research question is well-defined and relevant, focusing on the role of melatonin in circadian rhythms and its therapeutic potential in various disorders, particularly Alzheimer's disease.	The study employs a comprehensive review of placebo-controlled clinical trials and recent scientific findings, ensuring a robust evaluation of melatonin's effects and applications.	Data analysis appears thorough, integrating results from multiple studies to draw conclusions about melatonin's efficacy and its impact on sleep quality, blood pressure, and neurodegeneration.	Results consistently show that melatonin is effective in treating circadian rhythm-related disorders and improving sleep quality. The link between melatonin deficiency and early Alzheimer's is particularly notable.	The review acknowledges the need for further studies to understand the causal relationships fully and the long-term effects of melatonin. The variability in individual responses to melatonin is also a noted limitation.
